# Tvp complexes support formation of the VgrG-PAAR spike during Type VI secretion system assembly

**DOI:** 10.1038/s44318-026-00820-1

**Published:** 2026-05-29

**Authors:** Laura Monlezun, Christopher Earl, Sarah J Coulthurst

**Affiliations:** 1 School of Life Sciences, University of Dundee, Dow Street, Dundee DD1 5EH, UK

## Abstract

Type VI secretion systems (T6SSs) are nanomachineries used by bacteria to inject toxic effectors into neighbouring cells during interbacterial competition or infection. Distinct paralogues of the structural proteins VgrG and PAAR and specific accessory proteins allow the same T6SS to deliver a wide range of effectors. We describe a new set of accessory proteins required for assembly of T6SSs containing the VgrG1-Paar1 spike and delivery of a novel VgrG1-associated membrane-targeting effector in *Serratia marces-cens*. TvpAB, TvpB, TvpC and Paar1 form a pre-complex essential for T6SS assembly, with VgrG1 replacing TvpC in the final accessory complex. Phylogenetic analysis and structural modelling reveal that Tvp proteins are widely conserved, but Tvp-containing complexes vary in organisation and complexity. We define three classes of Tvp complex, all sharing a DUF2169-containing TvpA protein which stabilises a DUF4150/PAAR-containing protein. Class I, which includes only TvpA, functions without a pre-complex, whereas Classes II and III involve additional Tvp components and two-step assembly. Our findings highlight how modular effector recruitment strategies underlie the versatility of the T6SS and suggest how alternative Tvp systems are tailored to promote assembly of secretion-competent, effector-loaded VgrG-PAAR spikes.

## Introduction

The Type VI secretion system (T6SS) is a contractile nanomachine used by many Gram-negative bacteria to deliver toxic effectors into neighbouring cells. Although it can mediate intoxication of eukaryotic cells or promote the secretion of metal-scavenging proteins, its major role lies in intra- and inter-species competition between bacteria (Coulthurst, 2019). The T6SS apparatus comprises 14 core components in four sub-complexes: the baseplate, the membrane complex, the sheath, and an expelled puncturing structure. T6SS assembly starts with the insertion of the membrane complex (TssJ, TssL and TssM) into the cell envelope, followed by the recruitment of the baseplate (VgrG/PAAR, TssE, TssF, TssG and TssK) to the inner face of the cytoplasmic membrane. The trimeric VgrG spike protein, sharpened by a conical PAAR protein tip, forms the central hub of the baseplate and its base serves as the scaffold for the polymerisation of a tube of stacked hexameric Hcp rings. Extending into the cytoplasm, this spear-like puncturing structure, comprising the VgrG-PAAR spike topping the Hcp tube, is surrounded by the sheath, composed of TssB and TssC subunits. Upon contraction of the sheath, also known as ‘firing’, the puncturing structure, loaded with effector proteins, is propelled across the envelope of the secreting bacterium and perforates the target cell to deliver the effectors (Wang et al, 2019).

T6SS effectors can be categorised based on their mode of delivery. Cargo effectors interact non-covalently with VgrG, PAAR or Hcp proteins within the puncturing structure, whilst specialised effectors comprise effector domains covalently attached to the C-terminus of one of these proteins (Jurėnas and Journet, 2021). Certain VgrG proteins contain C-terminal extensions that are not toxins but are required for the recruitment of other effectors. VgrG from EAEC, for example, includes a DUF2345 domain and a TTR domain, which are both important for Tle1 delivery (Flaugnatti et al, 2020). Additionally, adaptor proteins can be required for the loading of T6SS effectors onto the machinery. Two well-characterised classes of adaptor have been described: DUF4123-family proteins, which interact with a VgrG C-terminal extension and their cognate cargo effector to facilitate loading (Colautti et al, 2024), and DUF1795-containing (‘Eag’) chaperone proteins, which load PAAR-containing specialised effectors onto their cognate VgrG protein whilst protecting their transmembrane domains (Ahmad et al, 2020). Several studies have indicated that DUF2169-containing proteins may also function as T6SS adaptor proteins. In *Agrobacterium tumefaciens*, Atu3641/Tap2 was shown to be required for VgrG2-dependent delivery of the PAAR-containing Tde2 effector (Bondage et al, 2016), with a subsequent study reporting that genetic loci containing *vgrG* and *tap2* genes always encode a protein containing a DUF4150 (PAAR-like) domain and usually also an effector domain (Wu et al, 2021). Recently, an experimental structure was determined for a DUF2169 protein, and it was shown to interact with a DUF4150 protein to facilitate the formation of a functional VgrG-PAAR complex in *Vibrio* (Sachar et al, 2025).

*Serratia marcescens* is an opportunistic bacterium found in various environmental niches and is also responsible for causing hospital-acquired infections (Mahlen, 2011). The model *S. marcescens* strain Db10 possesses a single T6SS, which displays anti-bacterial and anti-fungal activities (Murdoch et al, 2011; Trunk et al, 2018). Ten T6SS effectors have been identified so far in Db10, three encoded in the main T6SS gene cluster and the others elsewhere in the genome (Trunk et al, 2018). The *S. marcescens* T6SS is associated with two VgrG proteins, VgrG1, encoded at the 5’ end of the T6SS gene cluster, and VgrG2 at the 3’ end, and can form three distinct assemblies based on specific VgrG-PAAR combinations (Cianfanelli et al, 2016). VgrG1 functions with the DUF4150-type Paar1 protein, whereas VgrG2 can function with two different specialised PAAR effectors, Rhs1 or Rhs2. Both the VgrG1- and VgrG2-containing T6SS assemblies can deliver Hcp-dependent cargo effectors (Ssp1-Ssp6, Tfe2), however, VgrG2-based assemblies deliver cargo effectors more efficiently than VgrG1-Paar1. Interestingly, whilst another three effectors, Rhs1, Rhs2, and Slp, are exclusively delivered by VgrG2, VgrG1-specific effectors remain to be identified (Cianfanelli et al, 2016).

**Figure 1:**
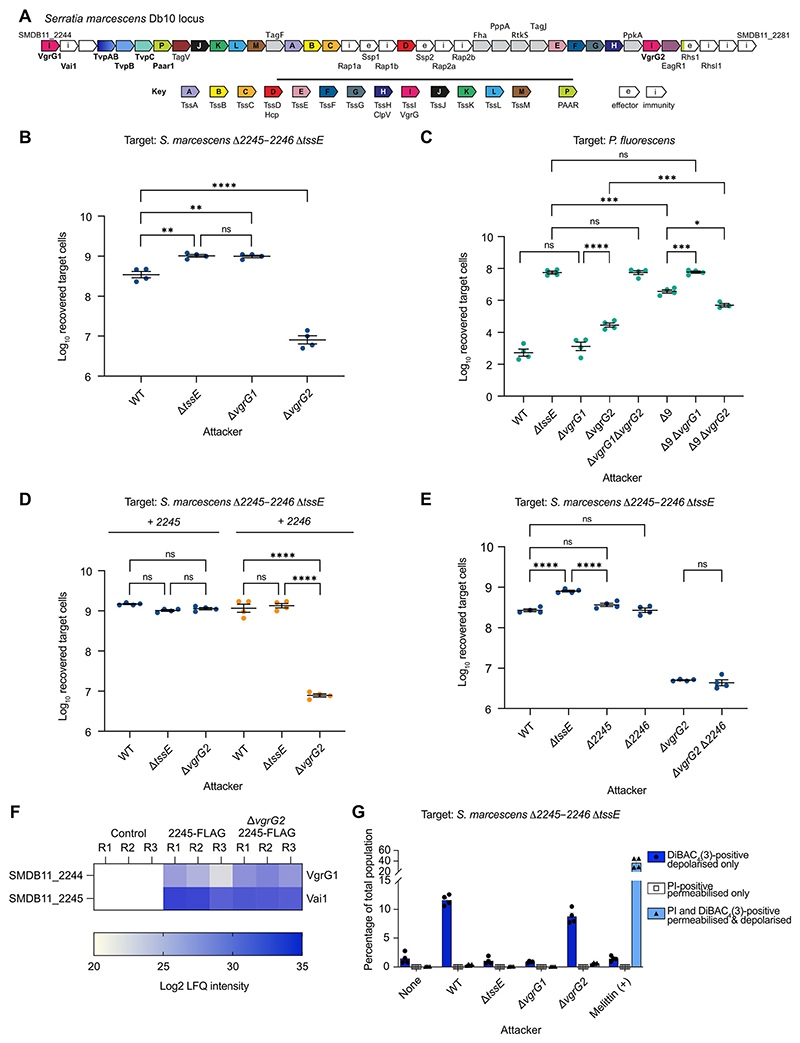
VgrG1 is responsible for a unique anti-bacterial activity that causes depolarisation of the target cell membrane and against which Vai1 provides immunity. **(A)** Schematic of the main T6SS gene cluster in *S. marcescens* Db10. Genes encoding core T6SS components are coloured and labelled with their corresponding Tss letters. **(B, E)** Recovery of *S. marcescens* Db10 Δ*2245*–*2246* Δ*tssE* target cells following co-culture with wild type (WT) and mutant strains of *S. marcescens* Db10 at an initial ratio of 1:1. **(C)** Recovery of *P. fluorescens* target cells following co-culture with WT and mutant strains of *S. marcescens* at an initial ratio of 1:1. (D) Recovery of the Δ*2245*–*2246* Δ*tssE* target complemented with either *SMDB11*_*2245* or *SMDB11*_*2246* expressed from the pSUPROM plasmid, following co-culture with WT and mutant strains of *S. marcescens* at an initial ratio of 1:1. **(F)** Heatmap of label-free proteomic quantification (LFQ) intensity values for proteins in the elution fractions of anti-FLAG immunoprecipitations from the control strain (WT Db10, untagged) or from strains expressing an N-terminal 3xFLAG fusion to SMDB11_2245 (Vai1) from the normal chromosomal location, in either the parental background (FLAG-2245) or Δ*vgrG2* (Δ*vgrG2* FLAG-2245). Only proteins exclusively detected or significantly enriched (log2 fold change >2, *p* < 0.05) in immunoprecipitation samples compared with the control samples are included. The experiment included three biological replicates of each strain (R1, R2, and R3). **(G)** The VgrG1-susceptible target (Δ*2245*–*2246* Δ*tssE*) was co-cultured with WT and mutant strains of *S. marcescens* and then membrane potential and membrane permeability of the mixed population was determined by staining with DiBAC_4_(3) and propidium iodide (PI) followed by flow cytometry analysis. The percentage of cells in the total co-culture population identified as being permeabilised only (positive for fluorescence from PI only), depolarised only (positive for fluorescence from DiBAC_4_(3) only), or simultaneously depolarised and permeabilised (positive for PI and DiBAC_4_(3)) is shown on the Y-axis. Bars show mean ± SEM, with individual data points overlaid (*n* = 4 independent experiments). **(B–E)** Data sets are displayed as mean ± SEM (*n* = 4 biological replicates) with individual data points overlaid. ****P < 0.0001, ns not significant; one-way ANOVA with Tukey’s test; for clarity, only selected comparisons are displayed. *P* values from left to right: **B)** *P* = 0.0018, *P* = 0.0022, *P* < 0.0001 and *P* = 0.9993. **(C)** *P* = 0.6240, *P* > 0.9999, *P* = 0,0003, *P* > 0.9999, *P* < 0.0001, *P* = 0.0005, *P* = 0.0003 and *P* = 0.0209. **(D)** *P* = 0.2982, *P* = 0.6353, *P* = 0.9886, *P* = 0.9519, *P* < 0.0001 and *P* < 0.0001. **(E)** *P* < 0.0001, *P* = 0.3063, *P* > 0.9999, *P* = 0.0004 and *P* = 0.9116. Source data are available online for this figure.

The 5’ end of the *S. marcescens* Db10 T6SS gene cluster contains five genes of unknown function (*SMDB11_2245-2249*) located between *vgrG1* and *paar1* (Fig. 1A), suggesting that they could be involved in the delivery of T6SS effectors by the VgrG1-Paar1 assembly. Here, we show that SMDB11_2247, SMDB11_2248, SMDB11_2249 and Paar1 form an accessory complex required for the VgrG1 pathway, which delivers a novel membrane-targeting anti-bacterial effector against which SMDB11_2245 provides immunity. Based on their newly identified functions, we name SMDB11_2247-2249 as TvpAB, TvpB and TvpC, for Tag protein chaperoning VgrG-PAAR complexes. Through phylogenetic analysis and structural modelling, we define three classes of Tvp system, all of which are proposed to have the common function of assembling a mature VgrG-PAAR spike. The Class I system is the simplest, represented by the VgrG-TvpA-PAAR complex of *A. tumefaciens*. Classes II and III require an intermediary TvpC-containing complex, which templates the formation of the final VgrG-containing accessory complex. Class II systems, represented by the VgrG1b-associated complex from *Pseudomonas aeruginosa*, are char-acterised by a specific family of TvpC proteins (DUF6484) and the presence of an additional component named TvpD. Class III, the most complex, is represented by the *S. marcescens* system studied experimentally here, with longer TvpC proteins (DUF3540) and pentapeptide repeat-containing TvpB proteins. Our findings reveal that assembly of a secretion-competent, effector-loaded T6SS spike and resulting effector delivery is more complex than previously appreciated and can involve multiple steps. We propose that Tvp accessory complexes play two roles in T6SS assembly: supporting interactions between specific VgrG and PAAR proteins and providing a means to occupy the cavity around the spike within the baseplate when not filled by effector domains.

## Results

### A novel VgrG1-dependent effector compromises the membrane potential of intoxicated bacterial cells and is neutralised by the immunity protein Vai1

**Figure EV1:**
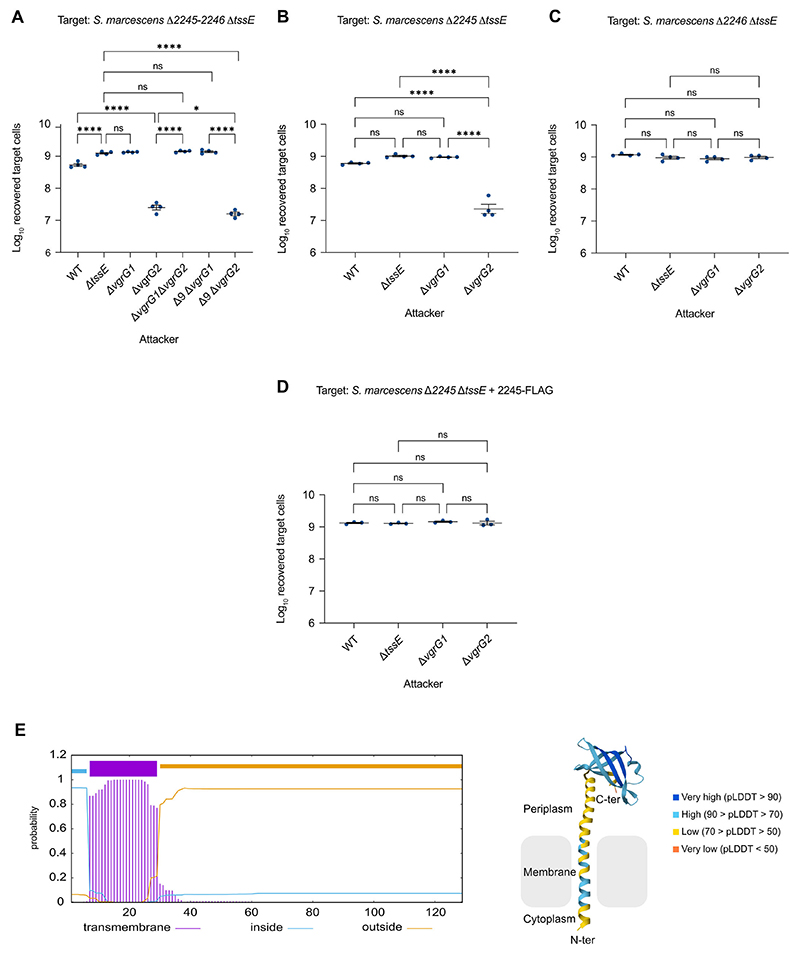
Vai1 is a new immunity protein conferring protection against the effector delivered by the VgrG1 pathway of *Serratia marcescens* Db10. **(A)** Recovery of *S. marcescens* Db10 Δ*2245*–*2246* Δ*tssE* target cells following co-culture with wild type (WT) and mutant strains of *S. marcescens* Db10. **(B)** Recovery of Db10 Δ*2245* Δ*tssE* target cells following co-culture with WT and mutant strains of Db10. **(C)** Recovery of Db10 Δ*2246* Δ*tssE* target cells following co-culture with WT and mutant strains of Db10. **(D)** Recovery of the Db10 Δ*2245*–*2246* Δ*tssE* target complemented with *2245*-FLAG expressed from plasmid pSUPROM, following co-culture with WT and mutant strains of Db10. In **(A–D)**, co-cultures were performed with an initial ratio of 1:1. **(E)** Left, Vai1 transmembrane helices prediction using TMHMM 2.0. The potential transmembrane helix in Vai1 (amino acids 7–29) is highlighted in purple. Right, Ribbon representation of the Vai1 structure predicted by AlphaFold2. The protein is coloured based on the reported confidence of the AlphaFold modelling, from orange (pLDDT <50, very low confidence) to dark blue (pLDDT >90, very high confidence). Vai1 insertion in the inner membrane is depicted based on the TMHMM prediction. **(A–D)** Data sets are displayed as mean ± SEM (*n* = 4 biological replicates in **(A–C)** and *n* = 3 biological replicates in **(D)**) with individual data points overlaid. ****P < 0.0001, ns not significant; one-way ANOVA with Tukey’s test; for clarity, only selected comparisons are displayed. *P* values from left to right: (A) *P* < 0.0001, *P* = 0.9977, *P* < 0.0001, *P* < 0.0001, *P* < 0.0001, *P* = 0.0376, *P* = 0.9592, *P* = 0.9864 and *P* < 0.0001. **(B)** *P* = 0.1750, *P* = 0.9850, *P* < 0.0001, *P* = 0.2922, *P* < 0.0001 and *P* < 0.0001. **(C)** *P* = 0.2949, *P* = 0.9139, *P* = 0.8451, *P* = 0.1090, *P* = 0.3699 and *P* = 0.9982. **(D)** *P* = 0.9893, *P* = 0.6671, *P* = 0.8060, *P* = 0.8286, *P* > 0.9999 and *P* = 0.9934. Source data are available online for this figure.

Since no T6SS effectors specific to VgrG1 had been identified in *S. marcescens* Db10, we considered whether two proteins of unknown function encoded directly downstream of *vgrG1*, SMDB11_2245, which contains a DUF3192 domain, and SMDB11_2246, represented a new effector-immunity pair (Fig. 1A). We generated a mutant lacking both genes in a T6SS-inactive background (Δ*tssE*), strain Δ*2245*–*2246* Δ*tssE*. If either SMDB11_2245 or SMDB11_2246 represented immunity proteins, this strain would be susceptible to T6SS-dependent intoxication by the wild type. In the case that either was an immunity protein but the other was not the cognate effector, a Δ*2245*–*2246* mutant would be subject to self-intoxication between sibling cells, resulting in lethality or a severe fitness defect. If the hypothetical effector acted from within the periplasm of intoxicated cells, as appeared likely due to the predicted localisation of SMDB11_2245 (see below), then inactivation of the T6SS (Δ*tssE*) would prevent any self-intoxication since the effector would never access the periplasm (English et al, 2012). We assessed the susceptibility of the Δ*2245*–*2246* Δ*tssE* mutant to T6SS-mediated anti-bacterial activity. Co-culture of this target with wild type Db10 as attacker resulted in reduced recovery of viable target cells compared to co-culture with a T6SS mutant attacker (Δ*tssE*) (Fig. 1B), indicating that the Δ*2245*–*2246* mutant lacks immunity against a T6SS-delivered effector. Deletion of *vgrG1* in the attacker cells abolished inhibition of the target, indicating that a VgrG1-dependent effector is responsible for this intoxication, while deletion of *vgrG2* greatly increased the anti-bacterial activity towards this target, consistent with our previous findings that secretion of VgrG1 is increased in the absence of VgrG2 (Cianfanelli et al, 2016). Moreover, the Δ9 Δ*vgrG2* mutant, lacking all known anti-bacterial effectors in Db10, was indistinguishable from Δ*vgrG2*, confirming that known effectors are not responsible for activity against Δ*2245*–*2246* (Fig. EV1A).

Using *Pseudomonas fluorescens* as a target, we next showed that the remaining anti-bacterial activity of the Δ9 strain, compared with a T6SS mutant, was dependent on VgrG1, being abolished in a Δ9 Δ*vgrG1* mutant. Recovery of *P. fluorescens* was 2-log lower using the Δ9 Δ*vgrG2* attacker strain compared with the T6SS mutant, also showing that the VgrG1-dependent effector can have considerable efficacy in an inter-species competition setting (Fig. 1C). Overall, these results show that *P. fluorescens* and the *S. marcescens* Δ*2245*–*2246* mutant are susceptible to a previously-unidentified VgrG1-dependent effector and that the delivery of this effector is increased in the absence of VgrG2, i.e., when there is no competition between the two VgrGs for assembly onto the T6SS machinery.

In order to determine which gene encodes the immunity protein, the intra-species co-culture experiment was repeated using a target with *SMDB11_2245* or *SMDB11_2246* deleted individually in a Δ*tssE* background. The Δ*2245* Δ*tssE* mutant remained sensitive to the anti-bacterial activity of the Δ*vgrG2* strain, whereas the Δ*2246* Δ*tssE* mutant was resistant (Fig. EV1B,C), indicating that SMDB11_2245 is the immunity protein conferring resistance to the effector delivered by the VgrG1 pathway. In addition, in trans complementation of the Δ*2245*–*2246* Δ*tssE* target strain confirmed that only SMDB11_2245 could provide protection against killing by the Δ*vgrG2* attacker strain (Fig. 1D). Importantly, the fact that the Δ*2245* Δ*tssE* mutant is viable and healthy indicates that the effector acts from within the periplasm. In an attempt to identify the cognate effector, Δ*2245* and Δ*2246* single mutants were generated in a wild type or Δ*vgrG2* background and tested as attackers against the Δ*2245*–*2246* Δ*tssE* target strain. Deletion of *SMDB11_2246* had no impact on activity against the Δ*2245*–Δ*2246* Δ*tssE* target (Fig. 1E), indicating that SMDB11_2246 is not the cognate effector, despite being encoded next to the immunity gene. Deletion of *SMDB11_2245* led to a slight reduction in anti-bacterial activity in the wild type background, and could not be achieved in the absence of suppressor mutants in the Δ*vgrG2* background, likely due to self-toxicity. The exclusion of SMDB11_2246 as a cognate effector of SMDB11_2245, was further confirmed by expressing SMDB11_2246 in *E. coli* MG1655 or in the *S. marcescens* susceptible mutant, Δ*2245*–*2246* Δ*tssE*, either in the cytoplasm or with an N-terminal OmpA signal peptide (OmpA_SP_) to direct it to the periplasm. In all cases, SMDB11_2246 expression did not show any sign of toxicity, whereas a known periplasmic effector, Ssp2, was toxic when expressed with OmpA_SP_ (Appendix Fig. S1A,B).

In order to identify the effector to which SMDB11_2245 provides immunity, a co-immunoprecipitation experiment was carried out using SMDB11_2245 with a C-terminal 3xFLAG tag (2245-FLAG) as bait, having first verified that the 3xFLAG tag did not impair the protective function of SMDB11_2245 (Fig. EV1D). The immunoprecipitation was conducted under the same conditions as the anti-bacterial co-culture assays and in a wild type and Δ*vgrG2* background. The only protein identified in the immuno-precipitation, besides 2245-FLAG, was VgrG1 (Fig. 1F). This suggested that the effector function may reside within VgrG1, i.e. that VgrG1 might be a specialised effector with an effector domain at its C-terminus. Consistent with this idea, no additional unannotated ORFs which could encode an effector protein were identified in the vicinity of *SMDB11_2245*, which closely follows the *vgrG1* gene. VgrG1 contains 786 amino acids, 144 more than VgrG2, thus this extension could represent an effector domain. We tested whether this C-terminal extension (VgrG1 CTD) is toxic in an heterologous expression system, although no recognisable effector domain could be identified in this region (see below). Initially, the C-terminal extension of VgrG1 (amino acids 598-786) and, to a lower extent, the full-length protein, appeared toxic when expressed in *E. coli* with OmpA_SP_ (Appendix Fig. S2A), However, this toxicity was non-specific since it could not be rescued by co-expressing SMDB11_2245 and was likely due to inhibition of the Sec machinery used for artificially translocating the protein to the periplasm, since expression of VgrG1 CTD with a signal peptide recognised by the TAT machinery (SufI_SP_) did not show any sign of toxicity and expression of OmpA_SP_-VgrG1 CTD was not toxic in *S. marcescens* lacking SMDB11_2245 (Appendix Fig. S2B). As expected, no toxicity was observed on cytoplasmic expression of full-length VgrG1 (Cianfanelli et al, 2016) or VgrG1 CTD only. Attempts to isolate the toxic activity of VgrG1 using shorter versions of the C-terminal domain in heterologous expression systems proved unsuccessful. Therefore, it is possible that the firing of an intact VgrG1 into a target cell by the T6SS is vital for the mechanism of toxicity.

To gain insight into the nature of the VgrG1-dependent effector, TMHMM and AlphaFold predictions for the immunity protein were generated in order to identify the cellular compartment in which the effector acts. SMDB11_2245 was predicted to have an N-terminal transmembrane helix (aa 7–29) in the inner membrane, with the body of the protein located in the periplasm and primarily composed of β-strands (Fig. EV1E). This localisation, together with the observation that the Δ*2245*–*2246* Δ*tssE* mutant does not intoxicate itself, suggests that the cognate toxin either acts within the periplasm or on the inner membrane from the periplasm. In order to investigate the impact of VgrG1-dependent toxicity on the inner membrane of intoxicated bacterial cells, we co-cultured attacker cells delivering the effector with target cells susceptible to the effector (Δ*2245*–*2246*), then stained the cells with propidium iodide (PI) and DiBAC_4_(3) and analysed the total population by flow cytometry. DiBAC_4_(3) stains cells which have lost their inner membrane potential (depolarised cells), for example, if ions can flow freely across the membrane, whilst PI staining occurs upon formation of large, non-specific pores or loss of membrane integrity (permeabilised cells). Target cells were also treated with melittin, a natural anti-bacterial peptide which forms pores in cell membranes(Lee et al, 2013), as a control (Fig. 1G; Appendix Fig. 3A). Use of wild type Db10 or Δ*vgrG2* as attackers induced membrane depolarisation, with around 10% of the total population stained with DiBAC_4_(3) only, but this effect was not observed with the Δ*tssE* and Δ*vgrG1* attackers (Fig. 1G; Appendix Fig. 3B). The absence of an increase in DiBAC_4_(3)-positive cells using Δ*vgrG2* as the attacker compared with wild type Db10 can be explained by a significant reduction in the number of recovered target cells in this condition, even with a reduced attacker:target ratio and incubation time compared with the standard co-culture setting (Appendix Fig. 3C). Altogether, these data reveal that the VgrG1 pathway delivers a further anti-bacterial activity, either within or interacting with VgrG1, which distinguishes it from the VgrG2 pathway. Toxicity is mediated via an effect on the inner membrane of target cells, likely the formation of small ion channels. Based on the newly identified immunity function of SMDB11_2245 against this VgrG1-specific anti-bacterial effector, we name SMDB11_2245 Vai1 (VgrG1-associated immunity protein).

### TvpAB, TvpB and TvpC accessory proteins are essential for a functional VgrG1-Paar1 assembly

**Figure 2:**
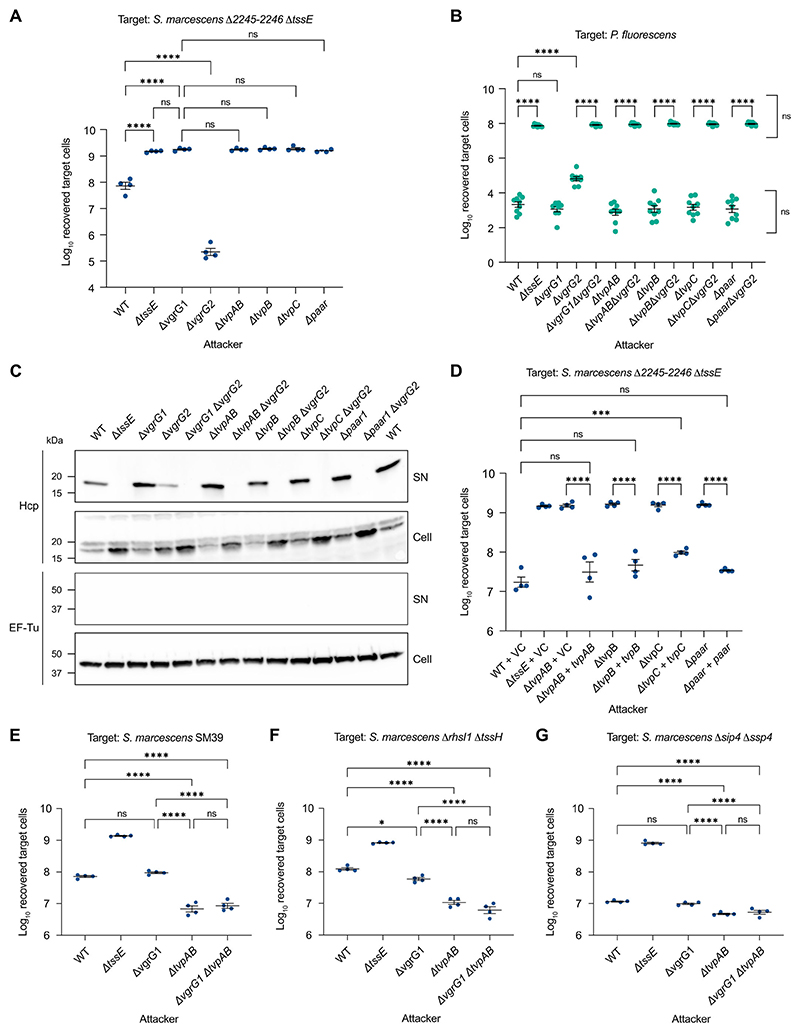
TvpAB, TvpB and TvpC are accessory proteins required for the VgrG1-Paar1 delivery pathway. **(A)** Recovery of *S. marcescens* Db10 Δ*2245*–*2246* Δ*tssE* target cells following co-culture with wild type (WT) and mutant strains of *S. marcescens* Db10 at an initial ratio of 1:1. **(B)** Recovery of *P. fluorescens* target cells following co-culture with WT and mutant strains of *S. marcescens* at an initial ratio of 1:1. **(C)** Levels of Hcp1 in total cellular (Cell) and secreted (SN) protein fractions from the indicated strains of *S. marcescens* as detected by immunoblot. The cytoplasmic protein, EF-Tu, is also detected as a control for loading and lysis. The results have been replicated in a second independent experiment. **(D)** Recovery of *S. marcescens* Δ*2245*–*2246* Δ*tssE* target cells following co-culture with WT and mutant strains of *S. marcescens* carrying either the vector control plasmid (pSUPROM, VC) or plasmids directing the expression of TvpAB, TvpB, TvpC or PAAR in trans, at an initial ratio of 1:1. **(E–G)** Recovery of *S. marcescens* SM39 (E), Db10 Δ*rhsI1* Δ*tssH* **(F)**, or Db10 Δ*sip4* Δ*ssp4* **(G)**, target cells following co-culture with WT and mutant strains of *S. marcescens* Db10 at an initial ratio of 1:1. Data sets are displayed as mean ± SEM (*n* = 4 biological replicates) with individual data points overlaid. ****P < 0.0001, ns not significant; one-way ANOVA with Tukey’s test; for clarity, only selected comparisons are displayed. *P* values from left to right: **(A)** *P* < 0.0001, *P* < 0.0001, *P* < 0.0001, *P* = 0.9963, *P* > 0.9999, *P* > 0.9999, *P* > 0.9999 and *P* = 0.9999. **(B)** *P* < 0.0001, *P* = 0.9510, *P* < 0.0001, *P* < 0.0001, *P* < 0.0001, *P* < 0.0001, *P* < 0.0001 and *P* < 0.0001 (grouped ns values against WT: *P* = 0.3799, *P* = 0.9684, *P* = 0.9996, *P* = 0.9645; grouped ns values against Δ*tssE* all *P* > 0.9999). **(D)** *P* = 0.7540, *P* = 0.1377, *P* = 0.0006, *P* = 0.5773, *P* < 0.0001, *P* < 0.0001, *P* < 0.0001 and *P* < 0.0001. **(E)** *P* = 0.5741, *P* < 0.0001, *P* < 0.0001, *P* < 0.0001, *P* = 0.7583 and *P* < 0.0001. **(F)** *P* = 0.0181, *P* < 0.0001, *P* < 0.0001, *P* < 0.0001, *P* = 0.0943 and *P* < 0.0001. **(G)** *P* = 0.5758, *P* < 0.0001, *P* < 0.0001, *P* < 0.0001, *P* = 0.8325 and *P* = 0.0005. Source data are available online for this figure.

The presence of genes encoding three other proteins, SMDB11_2247, SMDB11_2248 and SMDB11_2249, between *vgrG1* and *paar1* in *S. marcescens* Db10 (Fig. 1A) prompted us to investigate their possible role in the VgrG1 delivery pathway. Single in-frame deletion mutants of each gene were tested in a co-culture assay against the VgrG1-susceptible target, Δ*2245*–Δ*2246* Δ*tssE*. Loss of SMDB11_2247, SMDB11_2248, SMDB11_2249 or Paar1 abolished activity against this target strain in the same manner as deletion of *vgrG1* or *tssE* (Fig. 2A). In an inter-species competition, recovery of *P. fluorescens* was not affected by deletion of *SMDB11_2247, SMDB11_2248, SMDB11_2249* or *paar1* genes in a wild type background, since the more potent VgrG2 delivery pathway is still functional. However, anti-bacterial activity was fully abolished for these mutants in the absence of VgrG2, i.e. when only the VgrG1 pathway is present (Fig. 2B). The requirement of these genes for the functionality of the VgrG1 pathway was verified by examining Hcp secretion, a direct proxy of T6SS firing. Consistent with the existence of distinct functional T6SS assemblies in *S. marcescens* depending on the VgrG-PAAR pairing, neither the individual deletion of *vgrG1* or *vgrG2* abolished secretion of Hcp, although secreted Hcp levels were slightly lower in the Δ*vgrG2* strain and absent, as expected, in the Δ*vgrG1*Δ*vgrG2* mutant (Fig. 2C). In agreement with the results observed for T6SS-dependent anti-bacterial activity (Fig. 2B), deletion of *SMDB11_2247, SMDB11_2248, SMDB11_2249* or *paar1* genes prevented Hcp secretion in the absence of VgrG2 (Fig. 2C). Finally, complementation of these mutants by expression of the respective genes in trans restored T6SS activity against the *S. marcescens* Δ*2245*–*2246* Δ*tssE* target (Fig. 2D). Based on their essential accessory role in the VgrG1-Paar1 pathway, and an early study naming homologous genes *tagAB, tagB* and *tagC* (*tag, tss*-associated gene) (Shalom et al, 2007), SMDB11_2247, SMDB11_2248 and SMDB11_2249 were renamed TvpAB, TvpB and TvpC, respectively, for Tag protein chaperoning VgrG-PAAR complex.

We noticed that, in certain contexts, the Δ*tvpAB* mutant displayed increased anti-bacterial activity compared with the wild type strain. We explored this phenomenon against several targets, namely another strain of *S. marcescens* and two derivatives of Db10 sensitive to the Rhs1 and Ssp4 effectors, and observed that the Δ*tvpAB* mutant showed a clear increase in activity against these targets (Fig. 2E–G). Interestingly, such an increase was not observed with the Δ*vgrG1* mutant. The fact that only the Δ*tvpAB* mutant, but not the Δ*vgrG1* mutant, presents this phenotype indicates that the effect is not simply due to competition for the T6SS machinery between the VgrG1 pathway and the more efficient VgrG2 pathway. Moreover, enhanced activity was also observed for the Δ*tvpAB* Δ*vgrG1* mutant (Fig. 2E–G), confirming that the TvpAB repressive effect directly affects the VgrG2 pathway and is independent of the VgrG1 pathway. However, no protein–protein interactions between TvpAB and VgrG2 pathway components have been detected to explain this additional function of TvpAB (Cianfanelli et al, 2016) (and below). Nevertheless, these findings suggest that TvpAB mediates interference between the VgrG1 and VgrG2 pathways, although the underlying mechanism remains to be elucidated.

### The VgrG1 accessory complex requires the formation of a TvpC pre-complex

**Figure 3:**
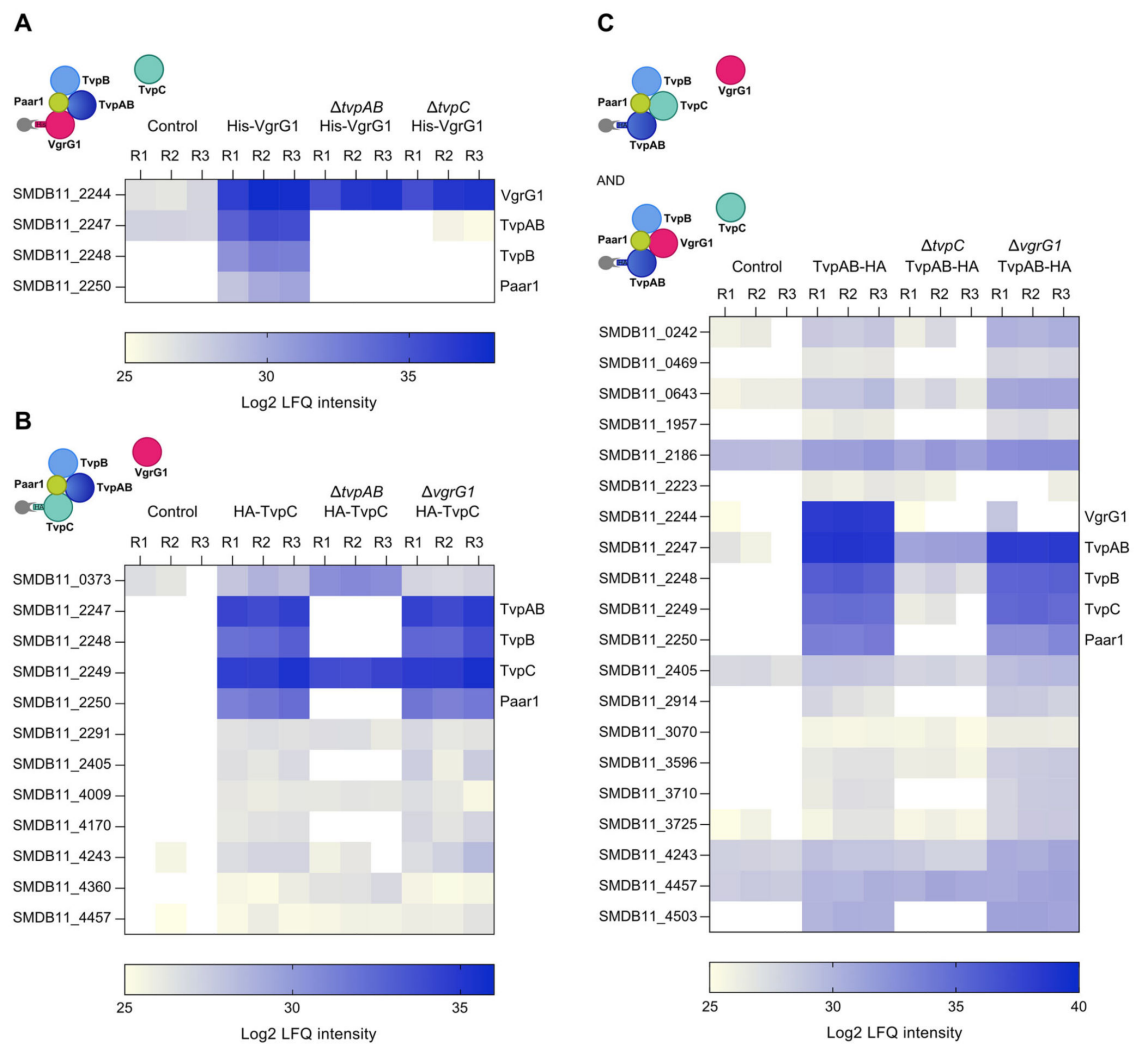
TvpAB, TvpB and Paar1 interact with TvpC and with VgrG1 but the presence of TvpC and VgrG1 in these complexes is mutually exclusive. **(A)** Heatmap of label-free proteomic quantification (LFQ) intensity values for proteins in the elution fractions of Ni-affinity purifications from the control strain (WT Db10, untagged) or from strains expressing a His_6_-VgrG1 fusion protein from the normal chromosomal location, in the parental (His_6_-VgrG1), Δ*tvpAB* (Δ*tvpAB* His_6_-VgrG1) or Δ*tvpC* (Δ*tvpC* His_6_-VgrG1) genetic backgrounds. **(B)** Heatmap of LFQ intensity values for proteins in the elution fractions of anti-HA immunoprecipitations from the control strain or from strains expressing an HA-TvpC fusion protein from the normal chromosomal location, in the parental (HA-TvpC), Δ*tvpAB* (Δ*tvpAB* HA-TvpC) or Δ*vgrG1* (Δ*vgrG1* HA-TvpC) genetic backgrounds. **(C)** Heatmap of LFQ intensity values for proteins in the elution fractions of anti-HA immunoprecipitations from the control strain or from strains expressing a TvpAB-HA fusion protein from the normal chromosomal location, in the parental (TvpAB-HA), Δ*tvpC* (Δ*tvpC* TvpAB-HA) or Δ*vgrG1* (Δ*vgrG1* TvpAB-HA) genetic backgrounds. In each case, only proteins exclusively detected or significantly enriched (log2 fold change >2, *P* < 0.05) in affinity purification or immunoprecipitation samples compared with the control samples are included in the heatmaps. Each experiment included three biological replicates of each strain (R1, R2 and R3). A schematic of the complexes identified in each pulldown/IP is depicted on the top left corner of the heatmaps. Full mass spectrometry data for each experiment are included in Dataset EV1.

The requirement for TvpAB, TvpB and TvpC in the VgrG1 delivery pathway suggested a potential interaction between those proteins and the T6SS spike. A strain encoding VgrG1 fused with an N-terminal His_6_-tag at the native chromosomal location was constructed, and functionality of the fusion protein was confirmed (Appendix Fig. S4A). His-VgrG1 was then affinity-purified from cell lysates, and co-purifying proteins were identified by quantitative mass spectrometry. Only three proteins were significantly enriched in the His-VgrG1 pulldown compared to the control, namely TvpAB, TvpB and Paar1 (Fig. 3A). Interestingly, although TvpC was essential for the functionality of the VgrG1 pathway (Fig. 2A–D), this protein was not detected among the VgrG1 interacting partners (Fig. 3A). To understand this discrepancy, we generated a strain encoding an N-terminally HA-tagged version of TvpC and confirmed its functionality (Appendix Fig. S4B). Identification of proteins significantly enriched in an HA-TvpC co-immunoprecipitation revealed TvpAB, TvpB, and Paar1 as the strongest hits, with no other T6SS proteins immunoprecipitated (Fig. 3B). Thus, once again, no interaction between TvpC and VgrG1 was detected. These results suggest the existence of two different complexes, a VgrG1 accessory complex and a TvpC alternative complex, which are mutually exclusive but share the same components, namely TvpAB, TvpB and Paar1. Consistent with this finding, when TvpAB with an HA tag at the C-terminus (Appendix Fig. S4C) was used as bait (Fig. 3C), all the components (Paar1, TvpB, TvpC and VgrG1) were co-purified.

Importantly, deletion of either TvpAB or TvpC prevented the formation of the VgrG1 accessory complex (Fig. 3A), while deletion of VgrG1 did not impact the formation of the alternative TvpC complex, since TvpAB, TvpB, TvpC, and Paar1 were still co-purified in Δ*vgrG1* TvpAB-HA and Δ*vgrG1* HA-TvpC immuno-precipitations (Fig. 3B,C). This indicates that formation of the TvpC alternative complex is a prerequisite for the formation of the VgrG1 accessory complex. Moreover, deletion of TvpAB also abolished the formation of the TvpC alternative complex (Fig. 3B), while deletion of TvpC considerably reduced the amount of bait recovered from the TvpAB-HA immunoprecipitation (Fig. 3C). Overall, our interaction studies identify a TvpC-containing pre-complex (TvpC-TvpAB-TvpB-Paar1) which is required for the subsequent formation of the VgrG1-containing accessory complex (VgrG1-TvpAB-TvpB-Paar1), with all of these components being essential for assembly and firing of T6SS machineries containing VgrG1-Paar1 as the spike complex.

### TvpC shares structural homology with VgrG proteins

To understand why the VgrG1 accessory complex and TvpC alternative complex are mutually exclusive while sharing the same partners, a structural prediction of TvpC was generated using AlphaFold2 and compared with predictions of VgrG1 and VgrG2.

**Figure EV2:**
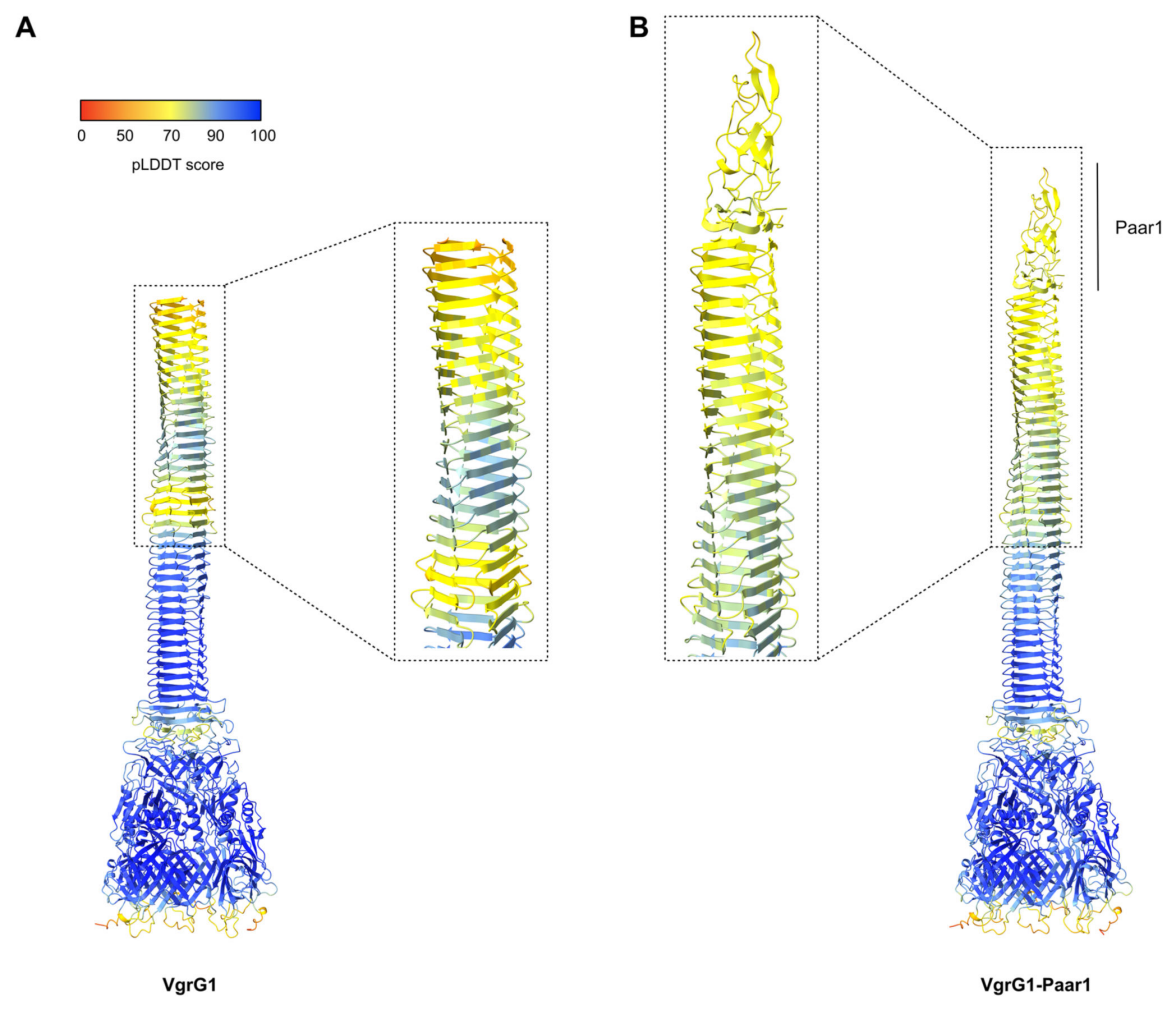
AlphaFold predictions of VgrG1 alone or in complex with Paar1. Ribbon representation of predicted structures of VgrG1 from S. marcescens Db10 **(A)** alone (as in Fig. 4) and **(B)** in complex with Paar1. A zoom on the VgrG1 tip is provided to show how it could be stabilised by Paar1 protein. Predicted structures were generated by AlphaFold2 and coloured according to the pLDDT score, from red (pLDDT <50, lowest confidence) to blue (pLDDT >90, highest confidence).

**Figure 4:**
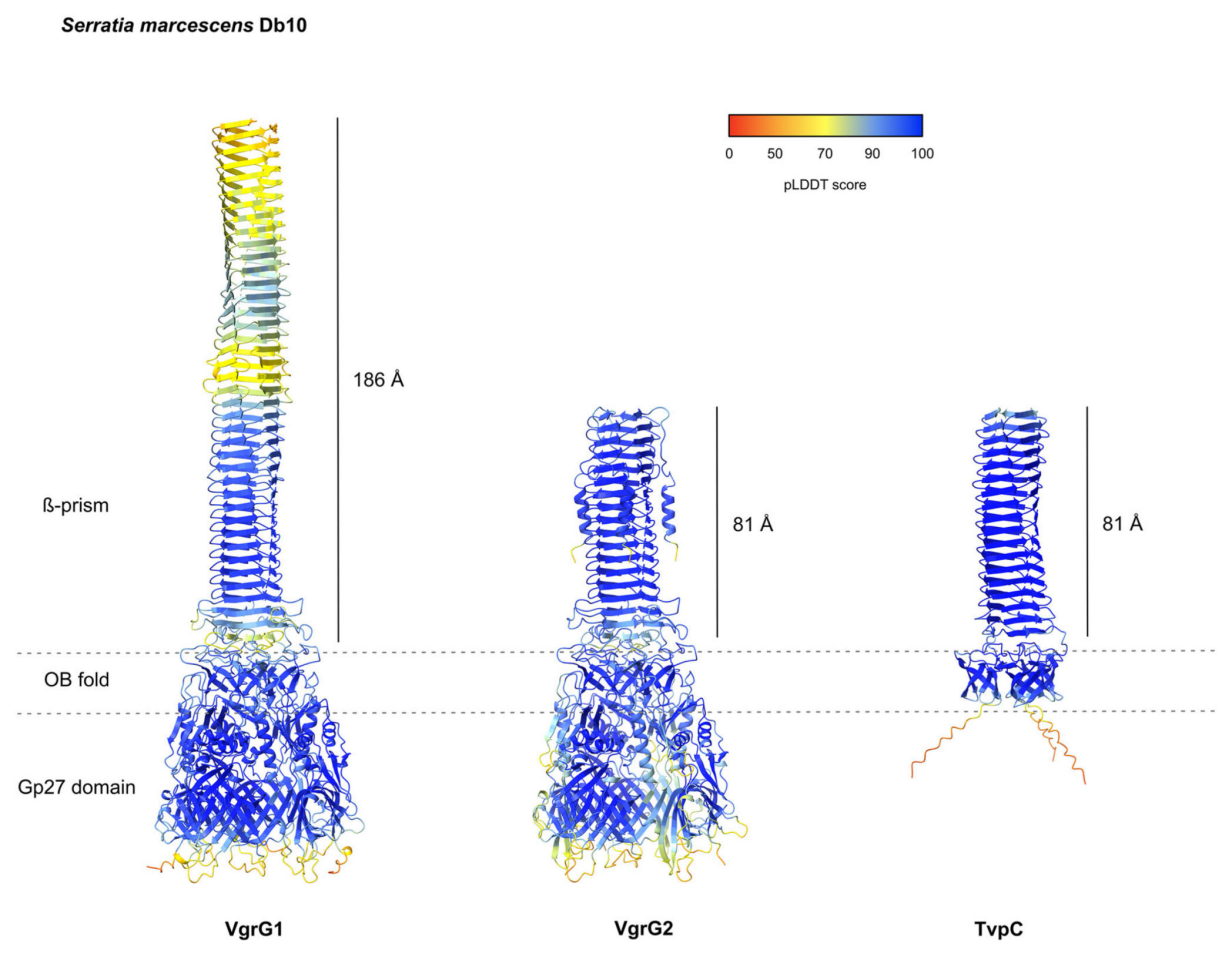
TvpC shares structural homology with VgrG proteins. Predicted structures of VgrG1, VgrG2 and TvpC from *S. marcescens* Db10. Ribbon representations are coloured according to the reported confidence of the AlphaFold2 modelling, from red (pLDDT <50, lowest confidence) to blue (pLDDT >90, highest confidence). The length of the β-prism is indicated next to each structure prediction. Source data are available online for this figure.

The TvpC protein is predicted to form a trimer and is composed of two domains, both folded with a high confidence score (pLDDT >90) (Fig. 4). These domains, an OB-fold and a β-prism, are also found in VgrG proteins. The main difference compared with VgrG proteins is the absence, in TvpC, of the Gp27 domain which interacts with the Hcp tube, and its replacement by a 16 amino acid unstructured tail at the N-terminus. This further suggests that the TvpC complex is an intermediate during the assembly of the T6SS machinery. Another striking feature of the structural comparison between the three proteins is the length of the β-prism. While TvpC and VgrG2 both have a β-prism 81 Å in length, VgrG1 has a very long β-prism of 186 Å (Fig. 4). Although the confidence score is somewhat lower for the second half of this long β-prism (pLDDT >70), it appears that the entire C-terminus of VgrG1 is part of this domain. Therefore, the VgrG1 C-terminus does not form a specific effector domain as is found in classical specialised VgrGs and which could readily explain the VgrG1-associated toxicity char-acterised above. Moreover, an AlphaFold prediction of the VgrG1-Paar1 complex confirms the presence of the long β-prism, whose tip seems to be further stabilised by the Paar1 protein, as evidenced by a higher pLDDT score for the VgrG1 extremity when complexed with Paar1 than when alone (Fig. EV2).

### Tvp accessory proteins are conserved in other species with a functional T6SS

**Figure 5:**
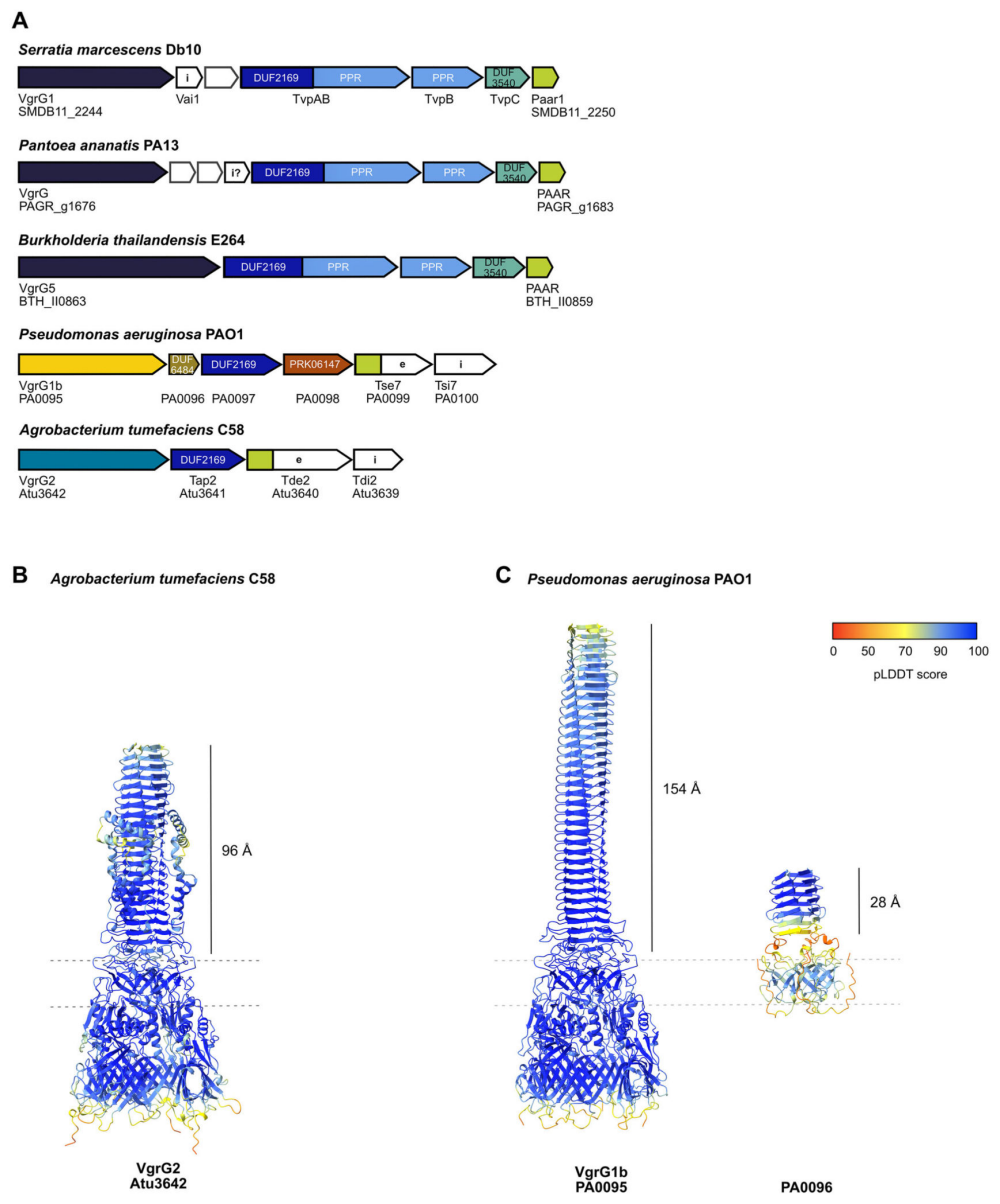
Tvp accessory proteins are conserved in many organisms with a functional T6SS. **(A)** Genetic context of Tvp and other VgrG accessory proteins in *Pantoea ananatis* PA13 (accession: CP003085.1), *Burkholderia thailandensis* E264 (accession: CP000085.1 and CP000086.1), *Pseudomonas aeruginosa* PAO1 (accession: AE004091.2) and *Agrobacterium tumefaciens* C58, now known as *A. fabrum* C58 (accession: AE007869.2). A larger view of the conservation of Tvp accessory proteins in other organisms is provided in Appendix Fig. S5. **(B)** Predicted structure of VgrG2 from *A. tumefaciens* C58. (C) Predicted structures of VgrG1b and PA0096 from *P. aeruginosa* PAO1. **(B, C)** Predicted structures were generated by AlphaFold2, and ribbon representations are coloured according to pLDDT score, from red (pLDDT <50, lowest confidence) to blue (pLDDT >90, highest confidence). The length of the β-prism is indicated next to each structure prediction. Source data are available online for this figure.

The remarkable length of the VgrG1 β-prism prompted us to investigate whether such a characteristic could explain the requirement for the Tvp accessory proteins. We looked for VgrG-associated accessory genes in other bacterial genomes, focusing on bacteria with an experimentally validated T6SS and whose genomes contain a gene encoding a protein with a DUF2169 domain. We discovered that a number of other organisms, including *Pantoea ananatis, Cronobacter sakazakii*, and *Burkholderia thailandensis* or *pseudomallei*, possess the same genetic organisation of *tvp-paar* accessory genes downstream of their *vgrG* genes as *S. marcescens* (Fig. 5A; Appendix Fig. S5). In addition, other organisms, like *A. tumefaciens*, harbour only a gene encoding a protein with a DUF2169 domain followed by a specialised PAAR effector gene. Others, like *P. aeruginosa* or *Proteus mirabilis*, possess two other putative accessory genes: a gene encoding a protein with a DUF6484 domain located downstream of *vgrG* and a gene encoding a protein with a PRK06147 domain located between the DUF2169 gene and a PAAR-containing effector gene (Fig. 5A; Appendix Fig. S5). In these two alternative configurations, the DUF2169-containing gene encodes a shorter homologue of TvpAB, which lacks the PPR domain and therefore can be described as a TvpA protein, whilst no homologue of TvpB could be identified. In all of these *tvp*-like clusters, the *vgrG* genes encode VgrG proteins which are one of at least two VgrGs associated with that T6SS and which are usually 700–800 amino acids long, except for VgrG5 from *Burkholderia* which has a characterised C-terminal effector domain (Schwarz et al, 2014). Therefore, the presence of Tvp proteins seems to be associated with alternative and rather long VgrG proteins, and their role in all these organisms is likely to be to chaperone alternative VgrG-PAAR assemblies.

Based on the existence of these three distinct organisations of *tvp* accessory genes associated with *vgrG*, we used AlphaFold2 to predict the structure of the *tvp*-associated VgrG proteins from *A. tumefaciens* and *P. aeruginosa* as well as the structures of their respective Tvp-like accessory proteins. VgrG2 from *A. tumefaciens* (Atu3642) has a 96 Å β-prism (Fig. 5B) while VgrG1b from the H1-T6SS of *P. aeruginosa* (PA0095) has a 154 Å β-prism (Fig. 5C, left), making them intermediate in their length between VgrG1 and VgrG2 from *S. marcescens*. Both of their TvpA-like accessory proteins, namely Tap2 for *A. tumefaciens* (Atu3641) and PA0097 for *P. aeruginosa*, are predicted to adopt the same fold as the DUF2169 domain of TvpAB (Appendix Fig. S6B–D). Strikingly, PA0096 from *P. aeruginosa*, which contains the DUF6484 domain, adopts a fold similar to TvpC of *S. marcescens*, although its β-prism is only 28 Å long (Fig. 5C, right). This implies that the Tvp-like accessory proteins from *P. aeruginosa* likely also form an alternative pre-complex before interacting with VgrG1b.

### Predicted structures of Tvp accessory pre-complexes

**Figure EV3:**
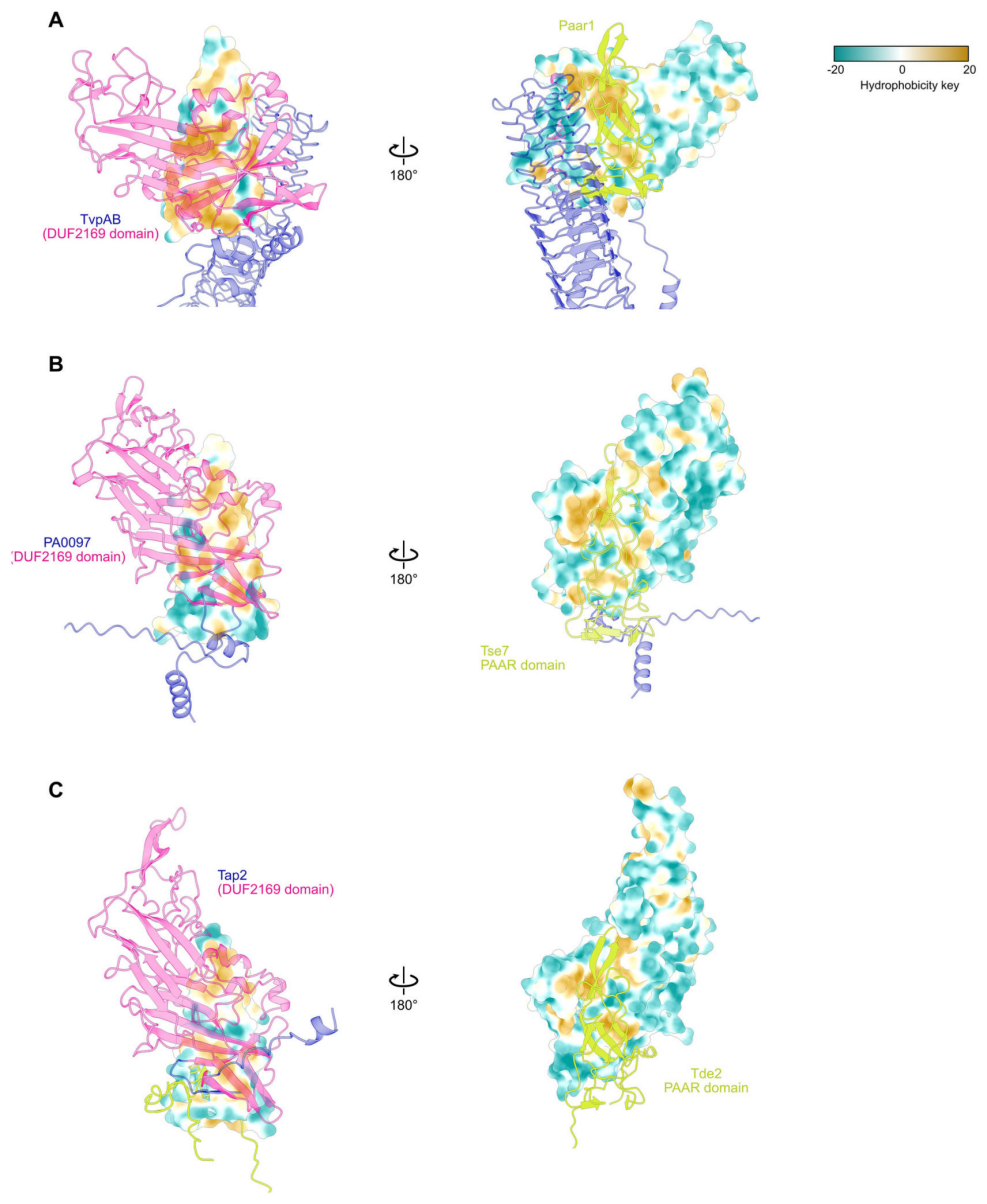
The interaction between the DUF2169 domain of TvpA/TvpAB proteins and the PAAR domain involves hydrophobic patches on both proteins. **(A)** Interaction between TvpAB and Paar1 from *S. marcescens* Db10. **(B)** Interaction between PA0097 and the PAAR domain of Tse7 from *P. aeruginosa* PA01. **(C)** Interaction between Tap2 and the PAAR domain of Tde2 from *A. tumefaciens* C58. **(A–C)** Left: The hydrophobic surface of PAAR domains is highlighted and TvpA/TvpAB proteins are represented as a ribbon with their DUF2169 domain coloured in deep pink. Right: The hydrophobic surface of the DUF2169 domains of TvpA/TvpAB is highlighted, whereas the PAAR domains are represented as a ribbon and coloured in light green. Hydrophobic surfaces are coloured from dark cyan (most hydrophilic) to white to dark golden (most lipophilic) as in the key.

**Figure 6:**
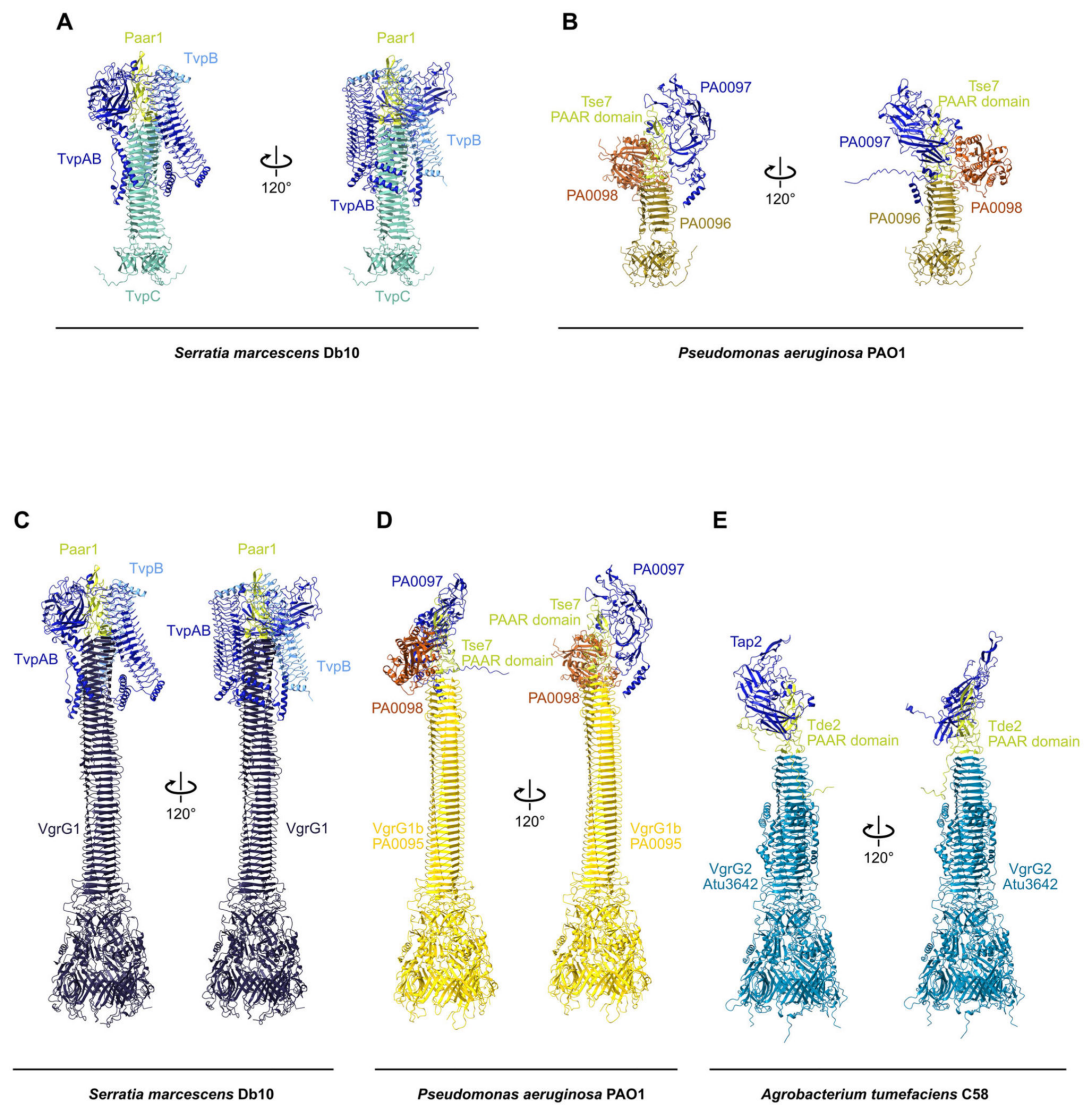
Predicted structures of accessory pre-complexes and VgrG-associated complexes of *Serratia marcescens, Pseudomonas aeruginosa* and *Agrobacterium tumefaciens*. **(A)** Ribbon representation of the predicted structure of the *S. marcescens* Tvp accessory pre-complex. TvpAB is coloured in medium blue, TvpB in light blue, TvpC in turquoise and Paar1 in light green. (B) Ribbon representation of the predicted structure of the *P. aeruginosa* accessory pre-complex. PA0096 is coloured in ginger, PA0097 in medium blue, PA0098 in rust and the PAAR domain of Tse7 effector in light green. **(C)** Ribbon representation of the predicted structure of the *S. marcescens* full VgrG1-associated accessory complex. VgrG1 is coloured in dark blue, and accessory proteins are coloured as in **(A). (D)** Ribbon representation of the predicted structure of *P. aeruginosa* full VgrG1b-associated accessory complex. VgrG1b is coloured in gold, and accessory proteins are coloured as in **(B). (E)** Ribbon representation of the predicted structure of *A. tumefaciens* full VgrG2-associated accessory complex. VgrG2 is coloured in sea blue, Tap2 in medium blue and the PAAR domain of Tde2 effector in light green. **(A, B, E)** Additional representation and colouring according to the pLDDT score are provided in Appendix Figs. S7 and S9. Source data are available online for this figure.

To gain insight into how the Tvp accessory proteins may chaperone VgrG1-Paar1 assembly, we first used AlphaFold2 to predict the structure of the TvpC pre-complex. This pre-complex was successfully modelled with a high confidence score (pLDDT >90, ipTM = 0.903) (Appendix Fig. S7A; Appendix Table S4). The predicted structure shows that Paar1 interacts with the extremity of the TvpC β-prism (Fig. 6a), similar to its interaction with VgrG. TvpAB then wraps around the TvpC-Paar1 into its concave groove, while TvpB acts as a “seat belt” to fasten the pre-complex (Fig. 6a; Appendix Fig. S7B). Thus, the tip of the TvpC-Paar1 structure is completely enfolded by the TvpAB-TvpB tandem. In this pre-complex, the DUF2169 domain of TvpAB interacts with Paar1, with surface analysis indicating that the interaction is mainly driven by hydrophobic regions of the two partners (Fig. EV3A). Other hydrophobic patches of Paar1 are masked by hydrophobic parts of the TvpB PPR domain. As a comparison, we also obtained an AlphaFold prediction of the *P. aeruginosa* pre-complex formed by PA0096 (DUF6484 and TvpC-like), PA0097 (TvpA), PA0098 (PRK06147) and the PAAR domain of Tse7. The confidence score of this pre-complex is slightly lower compared to *S. marcescens* (pLDDT >80, ipTM = 0.753), but the uncertainty is mainly concentrated on the extremities of PA0096 and PA0097 (Appendix Fig. S7C). In this predicted structure, the PAAR domain of Tse7 sits on the top of PA0096 β-prism, as in *S. marcescens*, while PA0097 and PA0098 form a concave groove protecting the PAAR domain of Tse7 (Fig. 6B; Appendix Fig. S7D). However, this chaperoning tandem is more open than in *S. marcescens*. The PA0097 DUF2169 domain shields the hydrophobic surface of the PAAR domain of Tse7, while the Tse7 hydrophilic face remains unprotected (Fig. EV3B). A structural prediction with the full sequence of Tse7 indicates that the effector domain does not close the belt around the PAAR domain but rather protrudes away from the pre-complex, outside of the PA0097-PA0098 face (Appendix Fig. S8). In addition, in the *P. aeruginosa* pre-complex, PA0096, the OB-fold/TvpC protein, barely interacts with PA0097, containing the DUF2169 domain, or with PA0098, since both of these proteins are shorter than their homologues in *S. marcescens* and devoid of a PPR domain.

**Figure EV4:**
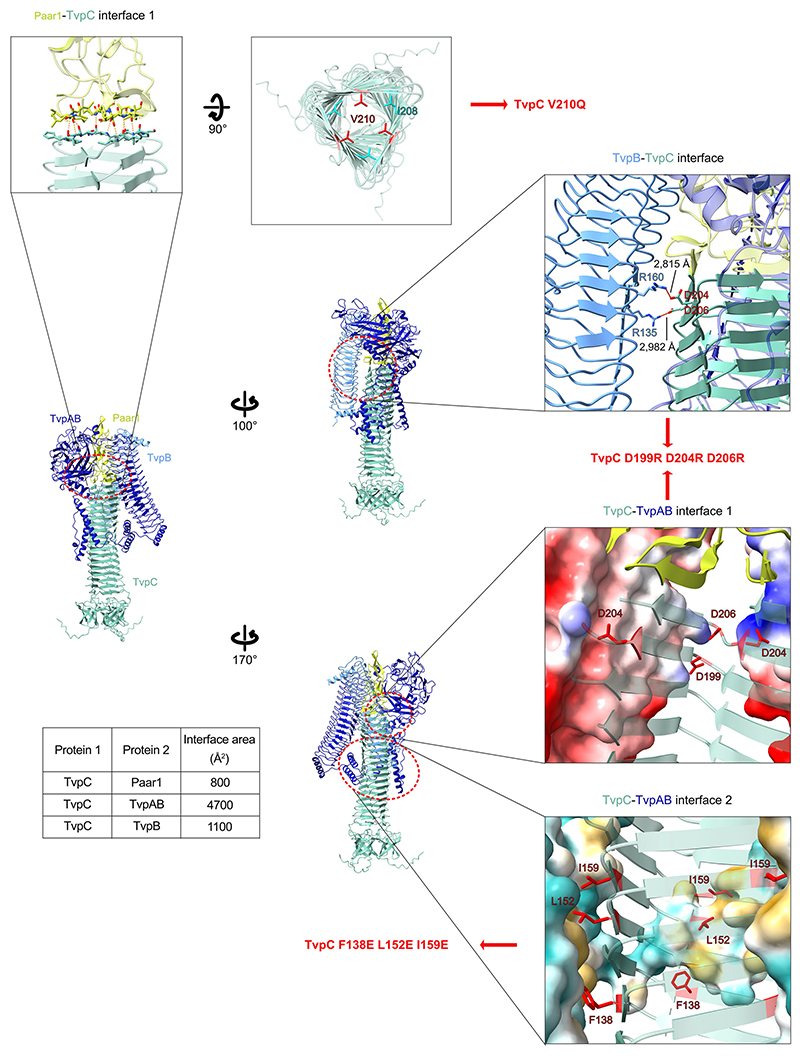
Structural view of TvpC amino acids selected for point mutations. Ribbon representation of *S. marcescens* pre-complex in different orientations with enlarged views of TvpC interfaces with TvpAB, TvpB and Paar1 within the pre-complex. Important residues at the interfaces are represented as sticks, and amino acids selected for substitution are coloured red. TvpAB electrostatic surfaces and hydrophobic surfaces are depicted for TvpC-TvpAB interfaces 1 and 2, respectively. A table summarising the interface areas between TvpC and its partner is provided.

To validate the pre-complex model, we designed point mutations at different protein–protein interfaces. We chose to mutate residues on TvpC since it is the central protein of the pre-complex, and its trimeric nature means that a single point mutation can affect three different faces within the complex. We focused on two main regions: one at the top of the TvpC-Paar1 interface, which is wrapped by TvpAB and TvpB, and one in the middle of TvpC, which is surrounded by TvpAB α-helices. The TvpC-Paar1 interface relies on a large network of hydrogen bonds with buried hydrophobic residues, especially TvpC V210 and TvpC I208 (Fig. EV4). Substituting TvpC V210 with a charged residue (V210Q) should disrupt this hydrophobic surface. The TvpC-TvpB interaction interface is relatively weak but appears to be stabilised, in particular, by two strong salt-bridges: TvpC D204-TvpB R160 and TvpC D206-TvpB R135 (Fig. EV4). Moreover, on other faces, TvpC D204 and TvpC D206, together with TvpC D199, are in close proximity to positively charged residues on TvpAB (Fig. EV4). Therefore, simultaneous substitution of these three aspartates with arginines is expected to destabilise interactions with both TvpB and TvpAB. Finally, the central part of the TvpC β-prism contains a few hydrophobic residues (TvpC I159, TvpC L152 and TvpC F138) deeply buried within the surface formed by the TvpAB α-helices (Fig. EV4). Substituting these residues with bulky, hydrophilic and charged amino acids, such as glutamate, should prevent these hydrophobic interactions.

**Figure 7:**
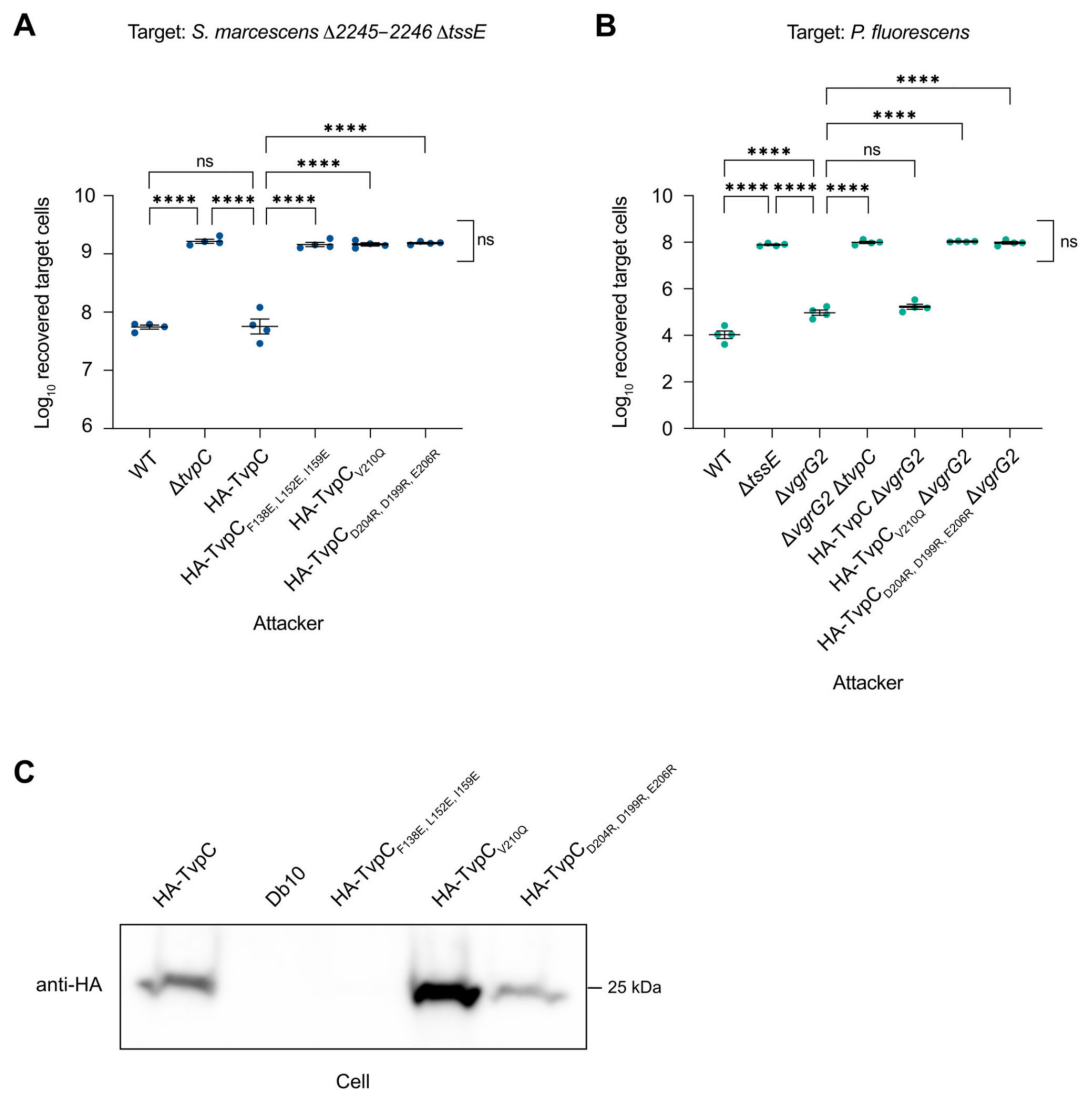
Mutations in TvpC predicted to disrupt its interaction with components of the Tvp pre-complex abolish function. **(A)** Recovery of *S. marcescens* Db10 Δ*2245*–*2246* Δ*tssE* target cells following co-culture with wild type (WT) and mutant strains of *S. marcescens* Db10 at an initial ratio of 1:1. Data were displayed as mean ± SEM (*n* = 4 biological replicates) with individual data points overlaid. ****P < 0.0001, ns not significant; one-way ANOVA with Tukey’s test; for clarity, only selected comparisons are displayed. *P* values from left to right: *P* < 0.0001, *P* < 0.0001, *P* < 0.0001, *P* > 0.9999, *P* < 0.0001, *P* < 0.0001, grouped ns values against Δ*tssE*: *P* = 0.9851, *P* = 0.9871 and *P* = 0.9989. **(B)** Levels of HA-TvpC protein in total cellular protein fractions from the indicated strains of *S. marcescens* as detected by immunoblot. The results have been replicated in a second independent experiment. **(C)** Recovery of *P. fluorescens* target cells following co-culture with WT and mutant strains of *S. marcescens* Db10 at an initial ratio of 1:1. Data were displayed as mean ± SEM (*n* = 4 biological replicates) with individual data points overlaid. ****P < 0.0001, ns not significant; one-way ANOVA with Tukey’s test; for clarity, only selected comparisons are displayed. *P* values from left to right: *P* < 0.0001, *P* < 0.0001, *P* < 0.0001, *P* < 0.0001, *P* = 0.4755, *P* < 0.0001, *P* < 0.0001, grouped ns values against Δ*tssE*: *P* = 0.9829, *P* = 0.9313 and *P* = 0.9932. Source data are available online for this figure.

We generated mutants of *S. marcescens* Db10 encoding HA-tagged versions of these three TvpC variants, namely TvpC_V210Q_, TvpC_D199R, D204R D206R_ and TvpC_F138E, L152E, I159E_, at the normal chromosomal location and tested the functionality of the VgrG1-dependent pathway in each case. Each variant resulted in a complete loss of activity against the VgrG1-susceptible target, Δ*2245*–Δ*2246* Δ*tssE* (Fig. 7A). As point mutations can compromise protein folding and stability, we assessed the presence of these three TvpC protein variants in *S. marcescens* cells by anti-HA immunoblotting. We observed that two of the three variants, TvpC_D199R, D204R, D206R_ and TvpC_V210Q_, are present at similar or higher levels compared with the wild type protein (Fig. 7b). In contrast, TvpC_F138E, L152E, I159E_ is almost undetectable, indicating a significant stability defect. Finally, we determined the impact of these variants on overall T6SS activity by performing a competition assay against *P. fluorescens*. This confirmed that both TvpC_D199R, D204R, D206R_ and TvpC_V210Q_, in a Δ*vgrG2* background, resulted in a complete loss of T6SS-dependent anti-bacterial activity (Fig. 7C). Thus, for at least two TvpC mutants, disrupting interactions with TvpAB, TvpB and Paar1 partners results in a loss of VgrG1-dependent T6SS activity without affecting TvpC protein level, validating the predicted structure of the pre-complex obtained using AlphaFold.

### Predicted structures of VgrG-containing full accessory complexes

We next attempted to model the full VgrG1-associated complex from *S. marcescens* using AlphaFold2, but the programme was unable to generate a prediction. In contrast, AlphaFold3 successfully predicted a shorter version of this complex using only the tip of VgrG1 (aa 542-786), with a pLDDT score >75. We used this prediction as a basis to manually dock the accessory proteins onto our predicted structure of the full VgrG1 (Fig. 4), allowing us to visualise how the full VgrG1 accessory complex could assemble (Fig. 6C). Strikingly, the overall organisation of the Tvp proteins is conserved between the TvpC pre-complex (TvpC-TvpAB-TvpB-Paar1) and the complex of TvpAB-TvpB-Paar1 with the tip of VgrG1, whilst the binding of the accessory proteins does not significantly modify the structure of the tip of VgrG1. This implies that the tip of VgrG1 will simply be further stabilised/rigidified by the Tvp proteins compared to the protein alone (Fig. 4) or in complex with only Paar1 (Fig. EV2B).

Using the same approach of combining an AlphaFold3 intermediate prediction with manual docking, we were also able to reconstitute the full VgrG1b-associated accessory complex of *P. aeruginosa* (Fig. 6D). In this case, AlphaFold3 produced a high-confidence model of VgrG1b in complex with the PAAR domain of Tse7 (pLDDT >85, ipTM = 0.776) that served as a basis to dock PA0097 (TvpA) and PA0098 using the PAAR domain of Tse7 for the fitting. The presence of the PAAR domain of Tse7 on the VgrG1b tip does not change the overall structure of VgrG1b. Furthermore, in this full VgrG1b-associated accessory complex, as in the pre-complex, neither PA0097 nor PA0098 directly interact with the β-prism of VgrG1b. Finally, we also obtained a structural prediction of VgrG2 from *A. tumefaciens* in complex with Tap2 and the PAAR domain of Tde2 using AlphaFold2 (pLDDT >85, ipTM = 0.778) (Fig. 6E; Appendix Fig. S9A,B). In this complex, the DUF2169 of Tap2 wraps the tip of the PAAR domain of Tde2. Again, this interaction relies on hydrophobic regions of both Tap2 and the PAAR domain of Tde2, whilst Tap2 does not make any contact with VgrG2 (Fig. EV3C; Appendix Fig. S9B). Overall, these structural predictions suggest that TvpA proteins containing only a DUF2169 domain do not directly interact with the VgrG tip. However, they can either act alone (*A. tumefaciens*) or in concert with an additional protein (*P. aeruginosa*) to chaperone the PAAR domain of effectors during the assembly of the T6SS machinery. In line with our Tvp nomenclature, we proposed to name the additional chaperoning protein in *P. aeruginosa* (PA0098, PRK06147) as TvpD.

### Classification of Tvp accessory systems

**Figure 8:**
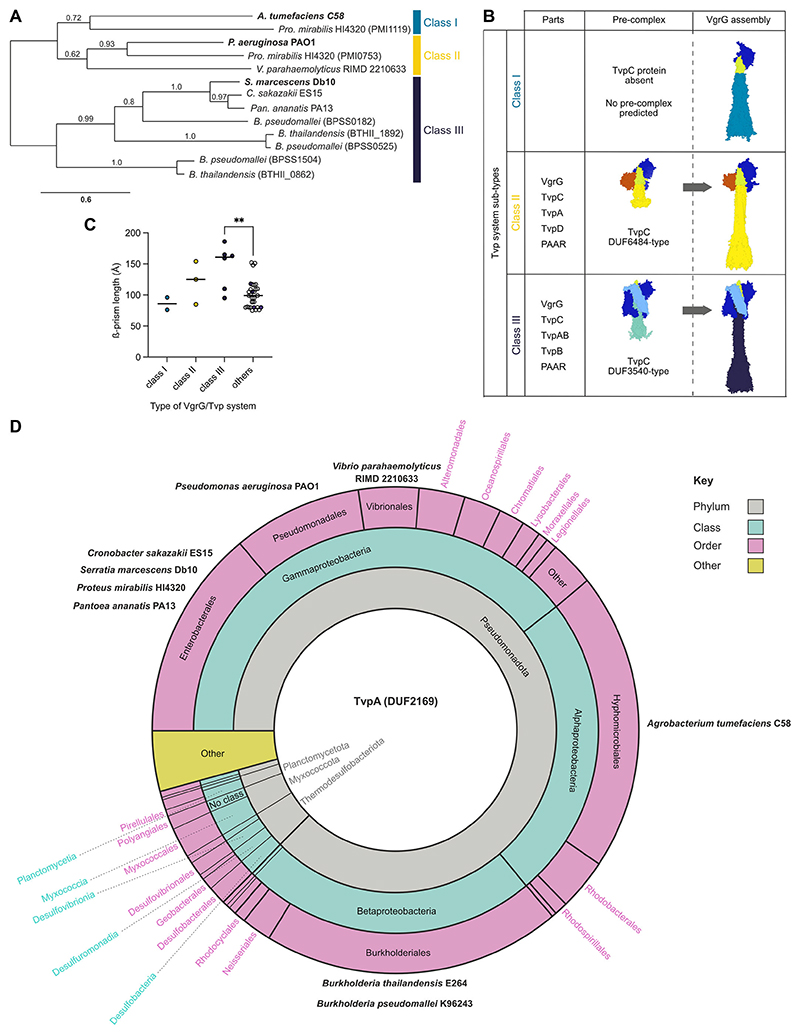
Classification of the Tvp system into Classes I, II and III based on structural, functional and phylogenetic comparisons. **(A)** Phylogenetic tree based on PAAR and TvpA as core components of the Tvp systems, from selected bacteria with an experimentally validated T6SS. Where strains contain multiple Tvp systems, the accession number for the TvpA protein is enclosed in brackets to distinguish between them. The tree was constructed from conserved PAAR-TvpA sequences which were concatenated and aligned using MUSCLE (Edgar, 2004; Mistry et al, 2021) (Appendix Fig. S10). **(B)** Graphic table summarising the three different classes of Tvp systems. The table illustrates the structure and function relationships between the Tvp systems of Class I (light blue), Class II (yellow) and Class III (dark blue), represented by *A. tumefaciens* C58, *P. aeruginosa* PAO1 and *S. marcescens* Db10, respectively. **(C)** Length of the β-prism of VgrG proteins in the set of eight representative bacteria (*S. marcescens* Db10, *A. tumefaciens* C58, *P. ananatis* PA13, *C. sakazakii* ES15, *P. mirabilis* HI4320, *P. aeruginosa* PAO1, *B. pseudomallei* K96243 and *B. thailandensis* E264) and in *V. parahaemolyticus* RIMD 2210633. Structural models of all the VgrG proteins were generated using AlphaFold3. Each model was visualised with PyMol to measure the length of the β-prism when possible. pLDDT and β-prism length values are summarised in Appendix Table S5. **(D)** Taxonomic distribution of TvpA (DUF2169) genes in available sequenced bacterial genomes. 4056 proteins with the InterPro entry IPR018683 (Pfam PF09937) (Mistry et al, 2021; Blum et al, 2025) are represented. Segments are weighted by the number of species. Source data are available online for this figure.

The diversity of Tvp systems means that phylogenetic classification is not straightforward. There are a number of components that are found in some systems but not in others, for example, TvpD or TvpB. Additionally, although a core conserved unit of VgrG-TvpA-Paar1 is a commonality, these individual proteins often contain divergent additional features and domains. VgrG sequences were found to be too variable to generate a useful phylogenetic classification, likely due to independent adaptations to facilitate species-specific interactions with baseplate components or effectors. For this reason, we compared the sequences and predicted structures of a number of PAAR and TvpA domains. A conserved sequence region corresponding to a shared structural arrangement was chosen for each protein (Appendix Fig. S10), and a phylogenetic tree was constructed using these TvpA-PAAR sequences concatenated (Fig. 8). The analysis included proteins originating from bacterial species with an experimentally validated T6SS, including the *V. parahaemolyticus* system in which the DUF2169 (TvpA) protein was very recently characterised (Sachar et al, 2025). This tree further emphasises the division of the different Tvp systems into three groups, with the *A. tumefaciens, P. aeruginosa* and *S. marcescens* systems serving as model representatives of each class (Fig. 8A), consistent with the three structural assemblies described above. Also consistently, the *V. parahaemolyticus* system, whose DUF2169 gene (VP1398) is flanked by genes including one encoding a protein with a DUF6484 domain (VP1395), falls into the same group as *P. aeruginosa*, which also possesses a DUF6484-type TvpC protein.

By combining our structural predictions of three representative assemblies and our phylogenetic analysis of *paar*-*tvpA*, we can define a functional classification for the Tvp accessory complexes (Fig. 8B): The simplest system, consisting of VgrG, PAAR and TvpA, is described as Class I and is represented by *A. tumefaciens* C58 (Bondage et al, 2016). Class II, represented by *P. aeruginosa* PAO1, is distinguished by a number of additional proteins which we predict, based on our observations in *S. marcescens*, will form a pre-complex using a short TvpC trimer protein (DUF6484), which is a partial structural mimic of VgrG with an OB-fold and very short β-prism. Class II systems have the unique property of having an additional predicted PAAR chaperone, which we call TvpD. Class III systems, represented by our *S. marcescens* Db10 system, have a longer TvpC protein (DUF3540) and contain an additional unique component, the pentapeptide-repeat domain (PPR) containing protein TvpB, which is included as a C-terminal extension of TvpA and an independent protein.

As noted above, VgrG1 in *S. marcescens* possesses an exceptionally long β-prism (Fig. 4A). Therefore, we performed a comparison with VgrG proteins from Tvp-containing, experimentally validated T6SS clusters, as well as other VgrG proteins encoded elsewhere within the corresponding genomes, to assess whether the length of the β-prism correlates with the number of accessory proteins. To this end, all VgrG structures were predicted using AlphaFold3 and the length of the β-prism was measured manually whenever possible (Appendix Table S5). Among the 45 VgrG predictions obtained, we excluded VgrG4a and VgrG4b from both *Burkholderia* species from our analysis due to inaccurate folding predictions of the β-prism tip. Despite the small sample size of Class I and Class II VgrG/Tvp systems, this measurement shows that, overall, VgrG proteins belonging to the Tvp Class III systems have a longer β-prism than other VgrG proteins, and this difference is significant (*P* value <0.05), at least compared to VgrG proteins which do not function with Tvp accessory proteins (Fig. 8C). However, given the distribution of β-prism lengths within each class, this is likely not the sole parameter driving the diversification of Tvp proteins.

Since TvpA is the common and defining feature of the three Tvp classes, we analysed the taxonomic distribution of Tvp systems by screening available sequenced bacterial genomes for the presence of *tvpA* (DUF2169) (Fig. 8D). This analysis showed that TvpA, which is classified as IPR018683 on the InterPro database and represented by 4056 protein sequences, is widespread and found in a number of clinically and environmentally important bacterial species. Most sequences belong to the phylum *Pseudomonadota*, but there are a number of other phyla represented (*Acidobacteriota, Myxococcota, Planctomycetota, Thermodesulfobacteriota*). Moreover, to validate our classification, we carried out an additional phylogenetic analysis using a more diverse set of concatenated sequences from the other bacterial phyla (Appendix Fig. S11). This showed that these diverse sequences accommodate perfectly into the same three functional and structural Tvp classes we defined, strengthening our classification (Appendix Fig. S12A,B).

## Discussion

In this study, we have defined a broad family of T6SS accessory complexes which all involve DUF2169-containing proteins and are subdivided into three classes, two of which mediate a previously undescribed two-step assembly process. Detailed characterisation of the Class III Tvp system of *S. marcescens* revealed that a TvpC-containing pre-complex precedes the formation of the full VgrG1-containing complex, which supports overall T6SS function and allows delivery of an unusual VgrG1-associated membrane-depolarising effector.

The T6SS is widely used by Gram-negative bacteria to deliver toxic anti-bacterial effector proteins into neighbouring rivals. Such effectors are encoded next to a partner gene encoding the cognate immunity protein, which provides the producing cell with protection against the effector, whether self-produced or delivered by a neighbouring cell. Here, we characterised Vai1, a new immunity protein in *S. marcescens*, which provides protection against an effector delivered through the VgrG1 pathway. Intriguingly, we were unable to formally identify its cognate effector using standard approaches, although an interaction with VgrG1 was observed by native co-purification. Since this interaction was observed only when using Vai1 as bait under conditions where T6SS-mediated protein delivery between cells can take place, it is likely to occur exclusively in target cells and not during the assembly of the machinery, where the VgrG1 tip will be shielded by accessory proteins. Vai1 is encoded immediately downstream of the *vgrG1* gene, the first gene of the T6SS cluster, suggesting that VgrG1 itself is likely to be the cognate effector. This idea is supported by the fact that the gene downstream of *vai1* does not encode an effector, the absence of effector candidates alongside Vai1 homologues in other genomes, and the lack of involvement of the other known T6SS effectors. However, despite having a longer C-terminus than VgrG2, no classical toxin domain could be identified in VgrG1, and this extension appears to form an elongation of the structural β-prism, making it unclear how VgrG1 exerts toxicity. An attractive hypothesis is that the C-terminus contains a cryptic toxic peptide, which would be toxic only when cleaved off from the full structural protein. Indeed, the last 26 residues at the C-terminus are largely hydrophobic, and an antimicrobial peptide prediction calculator (Wang et al, 2016) indicates that this region could exist as an α-helix with ten hydrophobic amino acids on the same face, when not part of the full VgrG1 structure. In this model, short amphipathic α-helices, released from intact VgrG1 proteins, would interact with the lipids of the target cell membrane and cause the membrane depolarisation we observed in intoxicated cells. The mechanism by which this putative antimicrobial peptide would be released from the VgrG1 protein is yet to be determined, although the Tvp accessory complex is likely to prevent it from occurring prior to secretion, and Vai1 may prevent it from occurring in resistant cells. Another, more unlikely, possibility is that the VgrG1-Paar1 spike forms a “mechanical toxin” similar to R-type tailocins from *P. aeruginosa*, whose physical action on the inner membrane induces depolarisation (Uratani & Hoshino, 1984). Resistance to this type of toxin is usually mediated through modification of the lipopolysaccharide receptor, rather than through the presence of a cognate immunity protein (Köhler et al, 2010; Mei et al, 2023). Thus, if VgrG1-Paar1 forms such a mechanical toxin, it remains unclear how Vai1 could intercept the incoming spike and prevent its toxic activity.

Loading of effectors into the T6SS machinery often involves accessory proteins, which can act as chaperones or adaptors to stabilise or guide them to the core machinery. DUF2169 proteins represent examples of such accessory proteins, and have been shown to be required for the delivery of Tde2 in *A. tumefaciens* (Bondage et al, 2016), and a PAAR-like DUF4150-containing effector in *V. parahaemolyticus* (Sachar et al, 2025). Here, we show that TvpAB, a DUF2169-containing protein, acts in concert with TvpB and TvpC to load the DUF4150-containing Paar1 protein of *S. marcescens* into the T6SS machinery, apparently by forming a pre-complex, where TvpC is subsequently replaced by VgrG1 in the full T6SS assembly. As reported for other chaperone/adaptor proteins (Jurėnas and Journet, 2021), we have never detected secretion of TvpAB or TvpB (Fritsch et al, 2013; Cianfanelli et al, 2016), therefore we predict that they are released from VgrG1 prior to its exit from the cell. Similar and also simpler versions of these Tvp accessory proteins exist in a large number of bacteria, and all of these accessory complexes share the presence of a DUF2169-containing protein that we call TvpA. Indeed, based on extensive structural predictions reinforced by phylogenetic analysis, we propose a classification of Tvp systems into three distinct classes, built upon a progressively increasing complexity in terms of both accessory components and assembly steps. Class I, exemplified by *A. tumefaciens*, is the simplest version, with a single accessory protein, TvpA, which stabilises the PAAR domain of Tde2 directly onto VgrG2. Class II, represented by *P. aeruginosa*, also includes a TvpA protein, which we predict will form a pre-complex with a TvpC-like protein and interact with the PAAR domain of the Tse7 effector before loading it onto the tip of VgrG1b. This class also contains another protein that we call TvpD, which could be classified as a co-chaperone for the PAAR domain. This aligns with a bioinformatic paper which speculates that PRK06147 may represent a novel T6SS adaptor (Liu et al, 2020). In both Class I and Class II systems, the accessory proteins are in close interaction with the PAAR domains of the effectors while barely interacting with VgrG proteins. Finally, Class III is the most sophisticated assembly and is represented by the *S. marcescens* Tvp system. Overall, this classification aligns with the three synteny categories described by Sachar et al (Sachar et al 2025), although we provide additional detail on the different groups, especially in terms of assembly. Our structural prediction for the TvpA domain of *S. marcescens* Db10 is very similar to the crystal structure of the *V. xiamenensis* DUF2169 protein, aligning with an RMSD of 1.076 Å (Appendix Fig. S13), confirming the accuracy of the prediction. Both *V. xiamenensis* and *V. parahaemolyticus* encode a DUF6484 protein, a hallmark of the Class II Tvp system, thus we can predict that T6SS assembly in these organisms will require the formation of a pre-complex similar to the one we modelled for *P. aeruginosa* (Fig. 6B). In addition, VP1399 in *V. parahaemolyticus*, which was not functionally characterised by Sachar et al (Sachar et al, 2025), likely encodes a TvpD protein chaperoning the PAAR domain of the VP1415 toxin, in association with the TvpA protein, VP1398.

Our analysis indicates that the DUF2169 domains of TvpA (*A. tumefaciens/P. aeruginosa*) and TvpAB proteins (*S. marcescens*) strongly interact with the DUF4150-type PAAR domains in all three classes, making this interaction a common structural feature of the different Tvp systems. This interaction seems to serve mainly to protect hydrophobic regions in PAAR domains, confirming that the DUF2169 domain is a PAAR chaperone. However, the extent of the hydrophobic regions in the PAAR proteins differs between the three systems. This results in a different orientation of the PAAR-TvpA tandem within the pre-complexes (when existing) or within the full complexes with VgrG proteins. Indeed, when structural models are superimposed by their PAAR domain (also referred to as a PIPY domain by Sachar et al), a moderate rotation of the DUF2169 is observed between Class I and Class II (~10°) while this rotation reaches almost 45° for Class III (Appendix Fig. S14A). When models are superimposed based on the short α-helix in a protruding loop of the DUF2169 domain, which is important for the interaction with the hydrophobic patch of PAAR (Sachar et al, 2025), again the β-sheets of the DUF2169 domain move by ~10° in Class II and by ~25° in Class III compared to Class I (Appendix Fig. S14B). This highlights the ability of the DUF2169 domain to adapt its chaperoning effect to the biophysical properties of its cognate PAAR domain.

Our findings that TvpC and VgrG1 form two mutually exclusive complexes (Fig. 3) provide new insights into the assembly in the T6SS machinery but also open new questions. Why might some T6SSs require the formation of pre-complexes (Tvp systems Classes II and III), while others do not (Tvp system Class I, and VgrGs with non-Tvp accessory proteins)? We propose a model whereby, in addition to their stabilising effect on PAAR protein, Tvp proteins interact with the T6SS baseplate and participate in its docking to the membrane complex, given that the VgrG trimer serves as the central hub of this baseplate and both VgrG and PAAR are required for the proper localisation of the baseplate during T6SS assembly (Cherrak et al, 2018; Beauvois et al, 2023). Two key characteristics of *S. marcescens* Paar1 and VgrG1 may necessitate the pre-complex for recruitment of baseplate components. First, Paar1 is a small protein, with a high degree of hydrophobicity and devoid of any other domains. Stability and interaction possibilities for Paar1 during T6SS assembly would be limited without the help of accessory proteins. Secondly, VgrG1 has a remarkably long β-prism. Such length means that the VgrG1-Paar1 tip (estimated at 242 Å) will penetrate deeper into the cavity of the membrane complex than for a typical VgrG and might disturb the closed resting state of the membrane complex, which is required for assembly of the T6SS machinery (Rapisarda et al, 2019). Thus, having a pre-complex with a VgrG-like protein, namely TvpC, harbouring a smaller, more typical β-prism would ensure the correct recruitment of the baseplate without compromising the integrity of the membrane complex. The fact that we cannot detect a VgrG1-TvpAB-TvpB-Paar1 complex in the absence of TvpC indicates that the TvpAB-TvpB-Paar1 ‘cap’ cannot assemble de novo on VgrG1, perhaps again because of the longer β-prism, but must first form on the shorter TvpC β-prism. We propose that this intact cap, within the baseplate, can then accept VgrG1. Whilst we believe that step-wise progression from a TvpC-containing pre-complex to a VgrG1-containing full-complex is the only way to explain our observations, the precise nature and mechanism of this inferred transition remain to be determined. For example, once the pre-complex is fitted within the baseplate cavity, new interactions between TvpAB-TvpB and baseplate components might loosen the interaction with TvpC so that the protein can exit the complex.

During initial studies, we looked for any interaction between Tvp accessory proteins and baseplate components using bacterial two-hybrid assays. We only observed an interaction between TssK and TvpC (Appendix Fig. S15A,B,D). Moreover, TssK also interacts with VgrG2, but not with VgrG1 (Appendix Fig. S15C,D). These data suggest that TvpC and VgrG2 could participate in the recruitment of baseplate components during the assembly of the T6SS apparatus, consistent with our model above. Several cryo-EM structures of baseplates from systems lacking such Tvp complexes instead highlight an interaction between VgrG and TssF wedges (Cherrak et al, 2018; Nazarov et al, 2018; Park et al, 2018). However, the absence of a Gp27 domain in TvpC and the high flexibility of TssK (Park et al, 2018) could result in variations in the overall shape and assembly of the T6SS baseplate between systems (Nazarov et al, 2018). The Gp27 domain of VgrG is required to promote Hcp tube polymerisation (Renault et al, 2018). Therefore, as TvpC does not possess a Gp27 domain, the T6SS machinery could not fully assemble on the pre-complex, meaning that TvpC has to be replaced by VgrG1 to allow Hcp polymerisation, concomitant sheath assembly and, ultimately, firing. The mechanism of this sequential assembly and proposed replacement of TvpC with VgrG1 remains to be fully elucidated in future studies.

The cryo-EM reconstruction of the *V. cholerae* baseplate highlighted the presence of a cavity formed by the baseplate periphery (TssK oligomers) around the central spike. Such a cavity was proposed to accommodate effectors associated with VgrG or PAAR (Nazarov et al, 2018). Moreover, Liang et al, demonstrated that some T6SSs have an “onboard checking” mechanism by which the presence of effectors loaded on the machinery is a prerequisite for T6SS secretion (Liang et al, 2019). In the absence of specific effector proteins or domains delivered by VgrG1 (Cianfanelli et al, 2016), this cavity could instead be filled with the Tvp accessory proteins in order to allow stable baseplate assembly and/or fulfil such an assembly checkpoint prior to firing. This hypothesis could explain why the VgrG1 pathway, without any effectors decorating the spike, is still able to assemble, fire and deliver Hcp-dependent effectors (Cianfanelli et al, 2016). In contrast, VgrG2 utilises large specialised PAAR proteins, Rhs1 and Rhs2. More generally, from our set of representative strains, it seems that Class III Tvp systems are found with small PAAR proteins without effector domains, whereas Class I and II systems are found with PAAR proteins containing specialised effector domains. Thus, the requirement for TvpAB-TvpB proteins could be linked to small PAAR proteins and a resulting need to occupy the baseplate cavity. In the *P. aeruginosa* Tvp system, which also forms a pre-complex, the PAAR domain is part of the Tse7 effector, thus the toxic moiety could partially fill the cavity, explaining why smaller Tvp proteins (TvpA and TvpD) would be required.

Collectively, our findings identify a new set of T6SS accessory proteins, named Tvp, which are associated with DUF4150-type PAAR proteins across many organisms and are required for the assembly and delivery of the corresponding VgrG-PAAR-containing puncturing structure with its payload of effectors. We have defined three classes of the Tvp system, which display increasing complexity in their composition and assembly. This variation likely reflects specific requirements for T6SS assembly, such as a need to accommodate the length of the VgrG β-prism and occupy space in the baseplate cavity not filled by effector domains. Our findings also suggest the existence of a new type of VgrG effector, which is able to induce loss of membrane potential in target cells in the absence of a separate effector domain. It is clear that the basic mechanism of the T6SS is highly versatile, with accessory proteins and complexes able to fine-tune and adapt its function.

## Methods

### Reagents and tools table

**Table:** 

Reagent/resource	Reference or source	Identifier or catalogue number
**Experimental models**		
Bacterial strains	This study	Appendix Table S1
**Recombinant DNA**		
Plasmids	This study	Appendix Table S2
**Antibodies**		
Custom rabbit anti-Hcp1 antibody	(Murdoch et al, 2011)	n/a
Anti-EF-Tu mouse monoclonal antibody	Hycult	HM6010
Anti-HA mouse monoclonal antibody	https://sls-reagents.co.uk/antibodies/ha	Clone 12CA5
HRP-conjugated anti-rabbit secondary antibody	Bio-Rad	170-6515
HRP-conjugated anti-mouse secondary antibody	Bio-Rad	170-6516
**Oligonucleotides and other sequence-based reagents**		
Oligonucleotide primers and synthetic gene fragments	This study	Appendix Table S3
**Chemicals, enzymes and other reagents**		
Tryptone	Thermo Fisher Scientific	LP0042
Yeast extract	Thermo Fisher Scientific	LP0021
Select agar	Thermo Fisher Scientific	30391023
Difco MacConkey Agar Base	Becton Dickinson (BD)	281810
Ampicillin	Formedium	AMP25
Kanamycin	Formedium	KAN0025
Chloramphenicol	Sigma-Aldrich	C0378
Streptomycin	Scientific Laboratory Supplies	S6501
L-arabinose	Thermo Fisher Scientific	A11921-30
D-glucose	Sigma-Aldrich	G7021
Maltose	Sigma-Aldrich	M5885
Toluene	VWR	28676.297
Glycine	VWR	10119CU
Invitrogen™ DiBAC_4_(3) (Bis-(1,3-dibutylbarbituric acid)Trimethine Oxonol)	Thermo Fisher Scientific	B438
Propidium iodide (PI)	Thermo Fisher Scientific	P3566
Melittin	Sigma-Aldrich	M2272
Phosphate-buffered saline (PBS)	Fisher UK	12559069
Tris (hydroxymethyl) aminomethane	Formedium	TRIS01
Sodium dodecyl (lauryl) sulfate (SDS)	Formedium	SDS0500
Ethylenediaminetetraacetic acid (EDTA)	Sigma-Aldrich	E6758
Glycerol	VWR	24388320
β-mercaptoethanol	Sigma-Aldrich	M6250
Bromophenol Blue	Sigma-Aldrich	114391
Triton X-100	Sigma-Aldrich	T8787
cOmplete™, EDTA-free Protease Inhibitor Cocktail	Roche	11873580001
Rabbit mAb bead conjugate	New England Biolabs	11846S
M2 magnetic beads	Merck	M8823
Ni-NTA magnetic agarose beads	Qiagen	36113
Protein LoBind Microcentrifuge Tube	Eppendorf	022431081
S-Trap™ micro spin columns	Protifi	C002- MICRO
PepMap™ nanoViper C18 column	Thermo Fisher Scientific	164750
PepMap™ RSLC C18 separation column	Thermo Fisher Scientific	ES903
**Software**		
FlowJo v10.4.2	Becton Dickinson	
MaxQuant, Perseus	https://maxquant.org/	
AlphaFold2	ColabFold hosted on the University of Dundee HPC cluster	
AlphaFold2 multimer v.3	ColabFold hosted on the University of Dundee HPC cluster	
AlphaFold3	hosted on https://alphafoldserver.com	
TMHMM 2.0	https://services.healthtech.dtu.dk/services/TMHMM-2.0/	
MUSCLE	https://www.ebi.ac.uk/jdispatcher/msa/muscle	
ClustalX	ClustalX version 2.0 accessed with Jalview	
UCSF ChimeraX version 1.9	https://www.rbvi.ucsf.edu/chimerax	
The PyMOL Molecular Graphics System, Version 3.0	Schrödinger	
Jalview	https://www.jalview.org/	
TreeDyn	https://www.phylogeny.fr/one_task.cgi?task_type=treedyn	
GraphPad Prism version 9.5.1	https://www.graphpad.com/	
**Other**		
LSRFortessa Cell Analyzer	Becton Dickinson	
QSonica Q800R Sonicator	QSonica	
Magnetic separation rack (MagnaRack™)	Invitrogen	CS15000
Q-Exactive™ Plus mass spectrometer	Thermo Fisher Scientific	
Dionex UltiMate3000 UHPLC system	Thermo Fisher Scientific	

### Bacterial strains, plasmids and culture conditions

The strains and plasmids used in this study are listed in Appendix Tables S1 and 2. Genetic variants of *S. marcescens* Db10 were generated by allelic exchange using the suicide plasmid pKNG101 as previously described (Murdoch et al, 2011). Streptomycin-resistant derivatives of Db10 strains were generated by introducing a SNP in *rpsL*, which resulted in asparagine being encoded at amino acid position 43. This SNP was either introduced by phage ΦIF3-mediated transduction of the resistance allele from *S. marcescens* Db11 or by recombineering using a modified version of the pORTMAGE plasmid (pSC3048) in conjunction with oligonucleo-tide CE75 carrying the desired SNP (Wannier et al, 2020). Plasmid-based constitutive expression of genes for complementation of gene deletions was achieved using the pSUPROM plasmid. Oligonucleo-tide primers and details of plasmid construction are given in Appendix Table S3.

Unless otherwise stated, bacterial cultures were grown in LB (10 g.L^−1^ tryptone, 5 g.L^−1^ yeast extract, 10 g.L^−1^ NaCl, with 1.8 g.L^−1^ agar for solid media) at 37 °C for *E. coli* and 30 °C for *S. marcescens* and *P. fluorescens*. When required, media were supplemented with antibiotics: carbenicillin (Ap) 100 μg.mL^−1^, chloramphenicol (Cm) 25 μg.mL^−1^, kanamycin (Kn) 100 μg.mL^−1^ and streptomycin (Sm) 100 μg.mL^−1^. For measurement of Hcp1 secretion by immunoblot, *S. marcescens* Db10 was grown in low salt liquid LB at 30 °C (10 g.L^−1^ tryptone, 5 g.L^−1^ yeast extract, 5 g.L^−1^ NaCl).

### Anti-bacterial co-culture assay to assess T6SS-mediated activity

We utilised our standard anti-bacterial co-culture assay (Murdoch et al, 2011). Attacker and Sm-resistant target strains of *S. marcescens* Db10 and its derivatives, *S. marcescens* SM39, or *P. fluorescens* 55 (Sm-resistant), were patched from a single colony and grown overnight on LB agar. Bacterial cells were recovered from the patches with a sterile loop and suspended in 1 mL of LB broth. Cell suspensions were normalised to an OD_600nm_ of 0.5, and attackers and targets were mixed at a 1:1 ratio. About 25 μL of the mixture was spotted onto solid LB and grown for 4 h (for inter-species assays) or 7.5 h (for intra-species assays). Following the co-culture, cells were recovered in 1 mL of LB broth, and the number of surviving target cells was enumerated by serial dilution, plating onto LB agar supplemented with Sm and determining viable counts.

### Plate toxicity assays

To test the impact of heterologous expression of proteins of interest from pBAD18-Kn-derived plasmids, fresh transformants of *E. coli* MG1655 or *S. marcescens* KT13 were resuspended in media, adjusted to an OD_600 nm_ of 1, and subjected to serial tenfold dilutions. About 5 μl of each dilution was spotted onto LB, M9 or minimal medium agar containing either 0.2% D-glucose or L-arabinose at 0.02 or 0.2%, based on procedures reported in (English et al, 2012).

### Membrane potential and membrane permeability analysis

A 25 μL mixture of attacker and target strains of *S. marcescens* Db10 were co-cultured at an initial ratio of 1:2 (attacker:target) on solid LB media for 4 h at 30 °C, then the cells were recovered and suspended in 1× phosphate-buffered saline (PBS) at 10^6^ cells/mL. Both DiBAC_4_(3) (Bis-[1,3-Dibutylbarbituric Acid] Trimethine Oxonol; Thermo) at 10 μM final concentration and propidium iodide at 1.5 μM final concentration were added simultaneously to each cell suspension, followed by incubation in the dark for 30 min. As a control, a cell suspension, derived from growth of a single colony on solid media, of the target strain was used to inoculate a 5 mL culture of LB low salt broth at a starting OD_600nm_ of 0.05 and cells were grown for 2.5 h at 30 °C, to reach an OD_600nm_ of ~0.5 (mid-log growth phase). Cells equivalent to 1 mL of cells at an OD_600nm_ of 0.1 were collected by centrifugation. The cell pellet was resuspended in 1 mL of PBS supplemented with melittin at 0.02 mg.mL^−1^ (final concentration of 6.32 μM) and incubated with agitation at 30 °C for 2 h, prior to staining. After staining with DiBAC_4_(3) and propidium iodide, cells were directly analysed in a FACS LRS Fortessa equipped with 488 and 561 nm lasers (Becton Dickinson), using thresholds on side and forward scatter to exclude electronic noise. Channels used were Alexa 488 (Ex 488 nm, Em 530/30 nm) for DiBAC_4_(3) and Alexa 568 (Ex 561 nm, Em 610/ 20 nm) for propidium iodide. All bacterial suspensions were normalised to ~10^6^ cells/mL prior to analysis. Analysis was performed using FlowJo v10.4.2 (Becton Dickinson).

### Immunodetection of cellular and secreted proteins

*S. marcescens* Db10, and derivatives were grown, aerated, for 5 h at 30 °C in 50 mL of low salt LB broth. Hcp1 detection was carried out on cellular and secreted fractions. Cellular protein samples were obtained by collecting 100 μL of culture from which cells were recovered by centrifugation. The cell pellet was resuspended in 100 μL of 2 x SDS sample buffer (100 mM Tris-HCl pH 6.8, 3.2% SDS, 3.2 mM EDTA, 16% glycerol, 0.2 mg.mL^−1^ bromophenol blue, 2.5% β-mercaptoethanol). Secreted protein samples were generated by combining 100 μL of culture supernatant with 100 μL 2 x SDS sample buffer. An amount equivalent to 1 mL of cells at an OD_600nm_ of 0.015 was loaded for the cellular fraction, and an amount equivalent to supernatant generated from 1 mL of culture at an OD_600nm_ of 0.015 was loaded for the secreted fraction. Hcp1 was detected using anti-Hcp1 rabbit primary antibody at a dilution of 1:6000 and horseradish peroxidase (HRP)-conjugated anti-rabbit secondary antibody (Bio-Rad #170-6515) used at a dilution of 1:10,000. EF-Tu and HA epitope tag was detected using mouse anti-EF-Tu antibody (Hycult #HM6010) at a dilution of 1:20,000, or anti-HA antibody (clone 12CA5) at a dilution of 1:10,000, respectively, and HRP-conjugated anti-mouse secondary antibody (Bio-Rad #170-6516) at a dilution of 1:10,000.

### Immunoprecipitation of epitope-tagged proteins with magnetic beads

Derivatives of *S. marcescens* Db10 encoding epitope-tagged versions of each protein of interest at the normal chromosomal location were generated by replacement of the wild-type allele of the gene. Bacterial cultures were inoculated at a starting OD_600nm_ of 0.025 and grown for 5 h in 50 mL of LB low salt broth to a final OD_600nm_ of ~2–2.5. Cells were recovered by centrifugation at 48,400 × *g* for 20 min, 4 °C. The cell pellet, equivalent to 50 mL of culture, was resuspended in 1 mL of ice-cold lysis buffer (Tris, pH 7.5, 20 mM, NaCl 150 mM, EDTA 0.5 mM, Triton X-100 0.1% + Roche complete EDTA-free protease inhibitor cocktail). Lysis was carried out on ice using sonication with a 1.6 mm microtip probe delivering 30% amplitude for 8 × 15 s with 30 s off between each pulse. Cell debris was removed by centrifugation at 20,000 × *g*, and the clarified lysate was incubated with 30 μL of magnetic anti-HA (Rabbit mAb bead conjugate from New England Biolabs, Ref. 11846S) or anti-FLAG (M2 magnetic beads from Merck, Ref. M8823) beads for 1–2h at 4 °C, 40 rpm. Beads were washed with 1 mL of lysis buffer three times, with a further wash carried out with lysis buffer which lacked Triton X-100. For elution, the beads were incubated with an SDS-based buffer (5% SDS, 50 mM Tris, pH 7.5 and 150 mM NaCl) for 5 min at 70 °C; the beads were then removed using a magnetic separation rack.

### Small-scale affinity purification of His-tagged proteins with magnetic NTA-beads

Strain construction, growth conditions and protein isolation procedures were the same for small-scale affinity purification (pull-down) as for the immunoprecipitation of epitope-tagged proteins, with the exception of the lysis and wash buffer chemistry. Lysis was carried out using a Tris-HCl 50 mM, NaCl 100 mM, Imidazole 20 mM + Triton X-100 0.5% lysis buffer with the sonication settings described above. The clarified lysate was incubated with 30 μL of Ni-NTA magnetic agarose beads (Qiagen #36113) for 1–2 h at 4 °C, 40 rpm. The wash buffer used was the same as the lysis buffer, but with the inclusion of 50 mM imidazole. Elution was performed using the same SDS-based elution buffer as above.

### Immunoprecipitation from cells grown on solid media

To determine interaction partners of the immunity protein Vai1 (SMDB11_2245), strains were grown on solid media, similar to the anti-bacterial co-culture assay, to permit T6SS delivery of effectors between cells. In this case, the strain was self-delivering the VgrG1-dependent effector. A single colony of each strain was patched and grown overnight on LB agar. Bacterial cells were recovered from the patches with a sterile loop and suspended in 1 mL of LB broth. Suspensions were normalised to 500 μL of OD_600nm_ = 2. The suspension was spotted as 10 × 50 µL spots on a single LB agar plate and grown for 5 h at 30 °C, and the spots were harvested in 10 mL of LB from the plate, which gave an OD_600nm_ of ~2. From this point onwards, the protocol was exactly the same as for immunoprecipitation with anti-FLAG M2 magnetic beads above.

### Mass spectrometry and label-free quantitation

Eluted protein samples in SDS elution buffer (5% SDS, 50 mM Tris, pH 7.5, 150 mM NaCl) were analysed by the ‘FingerPrints’ Proteomics Facility, University of Dundee. Protein samples were subjected to on-column tryptic digestion and clean-up using S-Trap^TM^ micro columns (Protifi). Proteins were reduced, alkylated and digested overnight with 1.9 µg trypsin per sample, followed by a second digest the next day.

Digested peptides were run on a Q-Exactive^TM^ Plus mass spectrometer (Thermo Scientific) coupled to a Dionex Ulti-Mate3000 UHPLC system (Thermo Scientific). LC buffers comprised buffer A (0.1% formic acid) and buffer B (80% acetonitrile, 0.1% formic acid) and were used to create a gradient lasting 156 min, with peptides trapped on a PepMap^TM^ nanoViper C18 column (Thermo Fisher Scientific Ref. 164750) before being eluted from a PepMap^TM^ RSLC C18 separation column (Thermo Fisher Scientific Ref. ES903). Peptides were eluted at a flow rate of 300 nl/ min (gradient increasing from 2 to 5% B (5 min), 5 to 35% B (125 min), 35 to 98% B (2 min), hold at 98% B (20 min), then decreasing to 35% B). The mass spectrometer was operated in positive ionisation mode, using an EasySpray source, with source voltage set to 2.85 or 3.00 kV (HA-TvpC) and ion transfer tube temperature 250 °C. Raw data were acquired in data-dependent mode. A scan cycle compromised of a full MS (MS1) scan followed by MS/MS (MS2) scans. For the MS1 scan, data were collected in profile mode with a resolution of 70,000, an Automatic Gain Control (AGC) target of 1E + 06, maximum Injection Time (IT) of 20 ms, microscan of 1 and a mass range of 350–1600 m/z. MS2 analysis was performed in centroid mode with a resolution of 17,500, an isolation window of 1.4 m/z, HCD collision energy of 27%, AGC target of 2E + 05, maximum IT of 100 ms, microscan of 1, scan range of 200–2000 m/z and a fixed first mass of 100 m/z. Dynamic exclusion was set to 45 s. RAW files were analysed in MaxQuant v1.6.6.0 (2245-FLAG), v2.1.0.0 (HA-TvpC), v1.6.2.10 (His-Vgr1 and TvpAB-HA), searching against a database of *Serratia marcescens* Db11 proteins, with variable modifications Oxidation (M), Acetyl (N-Term), Dioxidation (MW), Deamidation (NQ), Gln-pyroGlu (NQ), fixed modification Cardamidomethyl (C) and a false discovery rate (FDR) of 1%.

All samples were analysed in biological triplicate. Label-free quantitation was performed using MaxQuant (Tyanova et al, 2016a) as above, and comparative data analysis was performed using Perseus (Tyanova et al, 2016b) v1.6.21.0. Proteins were considered to be significantly enriched in abundance in test (epitope-tagged) samples compared with control samples if LFQ intensity was significantly increased according to the criteria: log2 fold change test/control >2, *p* < 0.05. Proteins were considered to be present in test and absent in control samples if detected in all three replicates of the test (with log2 LFQ intensity >25) and either not detected in any replicate of the control or in only one replicate of the control (when that single replicate gives log2FC test/control >3).

### Bacterial two-hybrid assay

Bacterial two-hybrid analyses were performed following established protocols (Karimova et al, 1998, 2000). *E. coli* MG1655 Δ*cyaA* was co-transformed with combinations of a pUT18-based and a pT25-based plasmid, and the colour of the resulting transformants was scored on MacConkey media with Ap, Cm and 0.2% maltose (positive result being red). For quantitative measurement of the interaction, β-galactosidase assays were performed as described (Murdoch et al, 2011) on double-transformed MG1655 Δ*cyaA* grown at 30 °C in LB and permeabilised with toluene. Replicate assays were performed on three independent transformants.

### Structural prediction and analysis

Protein and protein complex structural predictions were generated using AlphaFold2 and AlphaFold2 multimer v. 3 (hosted on the University of Dundee HPC cluster and implemented with ColabFold), or using AlphaFold3 (hosted on https://alphafoldserver.com) (Jumper et al, 2021; Evans et al, 2021; Abramson et al, 2024). Figures depicting AlphaFold predictions were prepared with ChimeraX (Meng et al, 2023). Structural predictions were superimposed using the Match-maker tool of ChimeraX, with the chain-pairing method when required, and with default fitting parameters. Structural comparisons were made using the structural alignment function in PyMol (The PyMOL Molecular Graphics System, Version 3.0, Schrödinger, LLC). The transmembrane domain of Vai1 was identified using TMHMM 2.0 (Sonnhammer et al, 1998).

### Phylogeny and taxonomy of Tvp systems

Multiple sequence alignments of concatenated TvpA and DUF4150 (PAAR) amino acid sequences were produced using the MUSCLE algorithm (Edgar, 2004), and coloured with ClustalX (Larkin et al, 2007). Alignment figures were prepared in the Jalview platform (Waterhouse et al, 2009; Troshin et al, 2011). The MUSCLE alignment was used for the generation of a phylogenetic tree. This was computed using phylogeny.fr (Dereeper et al, 2008) with PhyML (Guindon and Gascuel, 2003) using a WAG substitution model (Whelan and Goldman, 2001) and 500 bootstrap replicates. The output tree was visualised using TreeDyn (Chevenet et al, 2006). The taxonomic distribution of the TvpA protein was adapted from the InterPro (Blum et al, 2025) taxonomy graphic available for the DUF2169 classification entry (IPR018683).

### Statistical analysis and experimental design

Statistical analysis of proteomics data is described above. Otherwise, statistical analysis was performed using GraphPad Prism 9.

For competition assays, significant differences between independent biological replicates were determined based on Log_10_ transformed CFU values, using one-way ANOVA with Tukey’s post-test; data were tested for normal distribution using Shapiro Wilks test. Sample sizes (number of biological replicates) were chosen according to normal practice in the field and experimental feasibility. No blinding was performed.

## Expanded View Figures

## Supplementary Material

Appendix (supplementary data)

## Data Availability

The mass spectrometry proteomics data have been deposited to the ProteomeXchange Consortium via the PRIDE partner repository (https://www.ebi.ac.uk/pride/) with accession number PXD076681. All other data supporting the findings of this study are available within the paper and its supplementary information files. The source data of this paper are collected in the following database record: biostudies:S-SCDT-10_1038-S44318-026-00820-1. Expanded view data, supplementary information, appendices are available for this paper at https://doi.org/10.1038/s44318-026-00820-1.
