# Supplementary material for: Tvp complexes support formation of the VgrG-PAAR spike during Type VI secretion system assembly
Source: EMBO J. Author manuscript; Available in PMC 2026 Jul 21. (PMC13373197; doi:10.1038/s44318-026-00820-1)
Supplement: Appendix (supplementary data) [file EMS214941-supplement-Appendix__supplementary_data_.pdf]

Appendix

A family of widespread and modular accessory protein complexes support formation of the VgrG-PAAR spike of the Type VI secretion system

Table of Contents:

Appendix Figure S1 SMDB11_2246 is not toxic upon heterologous expression. **p.3**

Appendix Figure S2 The VgrG1 C-terminal domain does not display specific toxicity upon heterologous expression. **p.4**

Appendix Figure S3 Example of flow cytometry experiments from the analysis presented in Figure 1G. **p.5**

Appendix Figure S4 Epitope-tagged proteins used as bait in affinity purification and immunoprecipitation experiments retain function. **p.7**

Appendix Figure S5 Conservation of Tvp accessory proteins in representative Gram-negative species. **p.8**

Appendix Figure S6 Predicted structures of TvpAB, Tap2 and PA0097 proteins. **p.10**

Appendix Figure S7 Predicted structures of *Serratia marcescens* and *Pseudomonas aeruginosa* accessory pre-complexes. **p.11**

Appendix Figure S8 Predicted structure of the *Pseudomonas aeruginosa* accessory pre-complex with the full Tse7 effector. **p.13**

Appendix Figure S9 Predicted structure of the *Agrobacterium tumefaciens* VgrG2-associated accessory complex. **p.14**

Appendix Figure S10 Identification of conserved regions of the TvpA and PAAR DUF4150 proteins for phylogenetic analysis of the Tvp system. **p.15**

Appendix Figure S11 Alignment of an extended selection of PAAR, TvpA sequences for phylogenetic analysis. **p.17**

Appendix Figure S12 Extended phylogeny and taxonomy. **p.18**

Appendix Figure S13 Comparison of the Alphafold3 predicted structure of TvpAB from *Serratia marcescens* Db10 with the experimentally-determined structure of TvpA of *Vibrio xiamenensis*. **p.19**

Appendix Figure S14 Comparison of the orientation of the DUF2169 domain in the TvpA-PAAR complex between the different classes of Tvp systems. **p.20**

Appendix Figure S15 Interaction between TssK and TvpC by bacterial-two-hybrid assay. **p.21**

Appendix Table S1 List of strains used in this study. **p.22**

Appendix Table S2 List of plasmids used in this study. **p.24**

Appendix Table S3 Oligonucleotide primers and synthetic gene fragments used for plasmid construction. **p.26**

Appendix Table S4 Summary of the confidence scores of the Alphafold predictions presented in Figures 4 and 5. **p.31**

Appendix Table S5 Summary Table of VgrG β-prism length measurements. **p.32**

References References for Appendix. **p.34**

Appendix Figure S1

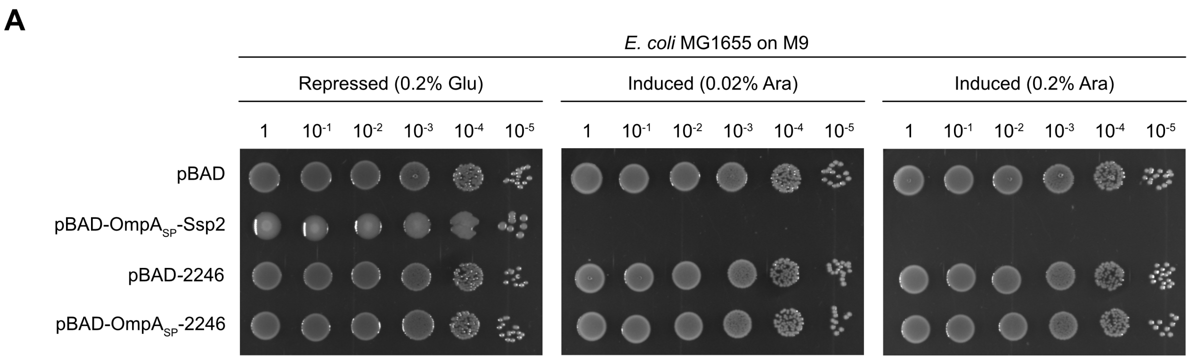

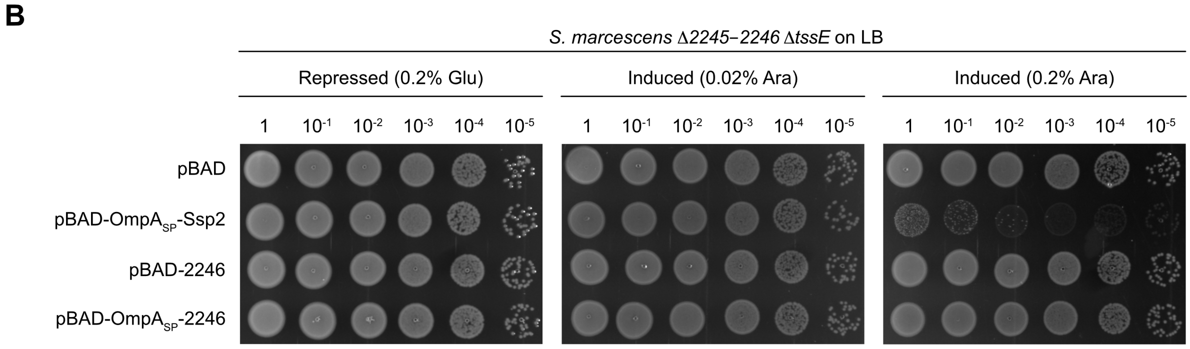

Appendix Figure S1. SMDB11_2246 is not toxic upon heterologous expression. (A) Growth on M9 minimal medium of *E. coli* MG1655 carrying empty vector control (pBAD) or plasmids directing the expression of native SMDB11_2246 (pBAD-2246) or SMDB11_2246 fused with an N-terminal OmpA signal peptide (pBAD-OmpA$_{SP}$-2246). (**B)** Growth on LB medium of *S. marcescens Δ2245-2246 ΔtssE* carrying the same constructs as in a). **(A-B)** Gene expression is repressed by the addition of 0.2% D-glucose or induced by the addition of 0.02% or 0.2% L-arabinose. pBAD-OmpA$_{SP}$-Ssp2 is used as a positive control for T6SS effector toxicity in the periplasm.

Appendix Figure S2

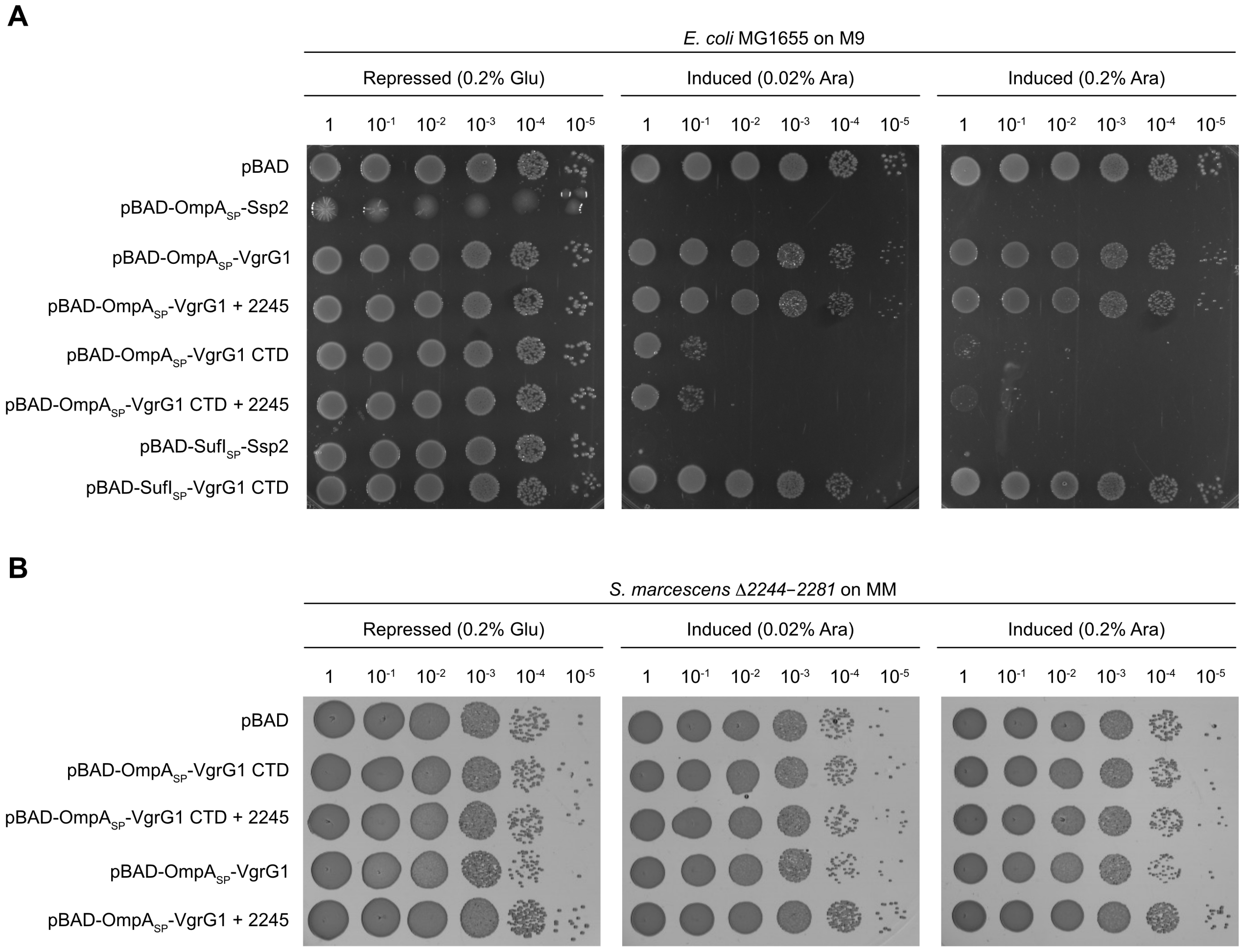

Appendix Figure S2. The VgrG1 C-terminal domain does not display specific toxicity upon heterologous expression. (A) Growth on M9 minimal medium of *E. coli* MG1655 carrying empty vector control (pBAD) or plasmids directing the expression of VgrG1 C-terminal domain (VgrG1 CTD, amino acids 598-786) or the full-length VgrG1, both fused with an N-terminal OmpA signal peptide (pBAD-OmpA$_{SP}$-VgrG1 CTD and pBAD-OmpA$_{SP}$-VgrG1 respectively). Co-expression of SMDB11_2245 is also tested for both VgrG1 constructs (pBAD-OmpA$_{SP}$-VgrG1 CTD + 2445 and pBAD-OmpA$_{SP}$-VgrG1 + 2245, respectively) and VgrG1 CTD expression is also tested with a N-terminal SufI signal peptide (pBAD-SufI$_{SP}$-VgrG1 CTD). pBAD-OmpA$_{SP}$-Ssp2 and pBAD-SufI$_{SP}$-Ssp2 are used as a positive control for T6SS effector toxicity in the periplasm. **(B)** Growth on minimal medium (MM) of *S. marcescens Δ2245-2281* carrying constructs as in A). **(A-B)** Gene expression is repressed by the addition of 0.2% D-glucose or induced by the addition of 0.02% or 0.2% L-arabinose.

Appendix Figure S3

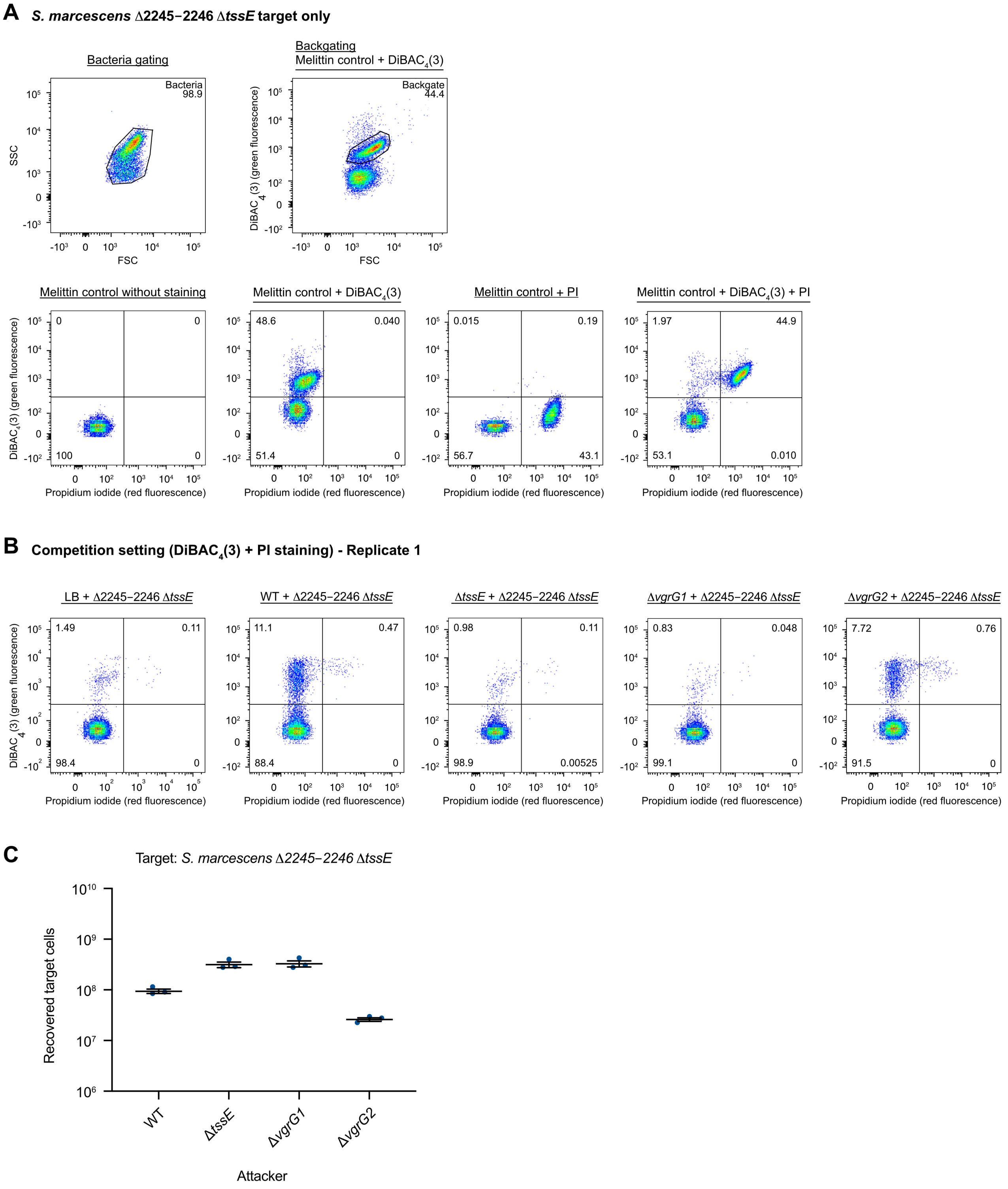

Appendix Figure S3. Example of flow cytometry experiments from the analysis presented in Figure 1G. (A) Analysis of *S. marcescens* Db10 *Δ2245-2246 ΔtssE* target only. Top panels illustrate the gating strategy. Left: Bacterial cells were selected using side scatter (SSC) vs. forward scatter (FSC). Right: Bacterial cells were treated with melittin, stained with DiBAC$_4$(3) (green fluorescence) and were selected using DiBAC$_4$(3) fluorescence vs. FSC. Bottom panels illustrate the quadrant strategy. Cells subjected to melittin treatment and stained with DiBAC$_4$(3) only define the depolarised cells quadrant (green fluorescence) while those treated with propidium iodide only were used to define the permeabilized cells quadrant (red fluorescence). Cells treated with melittin and stained with both DiBAC$_4$(3) and propidium iodide were used to establish the quadrant for cells that are simultaneously depolarised and permeabilized (green and red fluorescence). **B)** Analysis of bacteria following co-culture of *S. marcescens Δ2245-2246 ΔtssE* target with wild type (WT) or

mutant strains of Db10 for four hours at an initial attacker:target ratio of 1:2. After four hours of co-culture, cells were treated with both DiBAC$_4$(3) and propidium iodide. Data shown correspond to one of the four replicates presented in Figure 1G. **(C)** Recovery of the *S. marcescens* Δ*2245-2246* Δ*tssE* target following co-culture with WT or mutant strains under the conditions described in **(B)**. Data sets are displayed as mean +/- SEM (n=3) with individual data points overlaid.

Appendix Figure S4

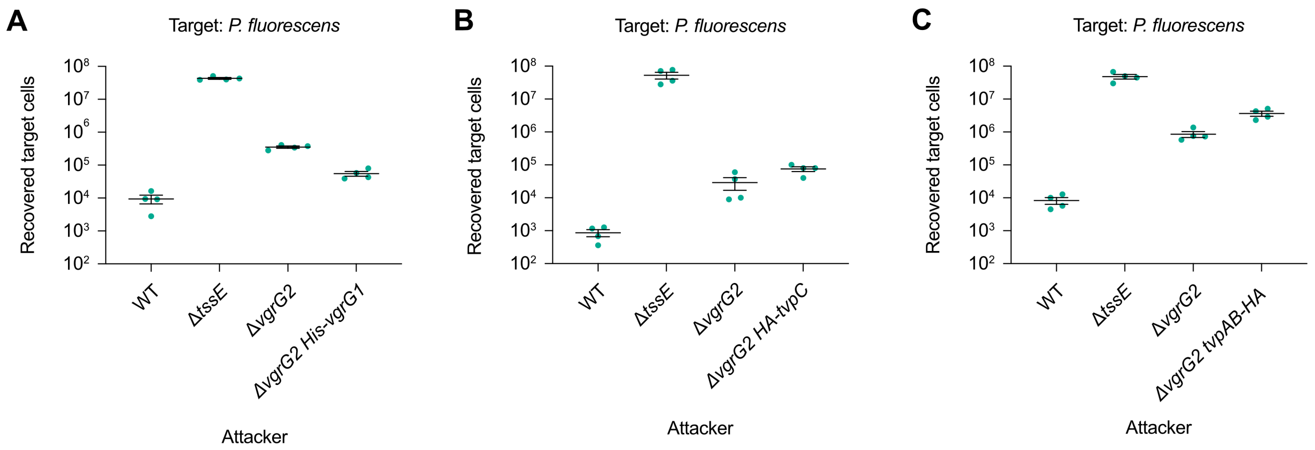

Appendix Figure S4. Epitope-tagged proteins used as bait in affinity purification and immunoprecipitation experiments retain function. (A-C) Recovery of *Pseudomonas fluorescens* target cells following co-culture with wild type (WT) and mutant strains of *S. marcescens* Db10 at an initial ratio of 1:1 for four hours. The functionality of the tagged proteins, namely His-VgrG1 **(A)**, HA-TvpC **(B)**, and TvpAB-HA **(C)**, was assessed in a Δ*vgrG2* background where loss of function in the VgrG1 pathway results in a complete loss of T6SS activity. Data sets are displayed as mean +/- SEM (n=4) with individual data points overlaid.

Appendix Figure S5

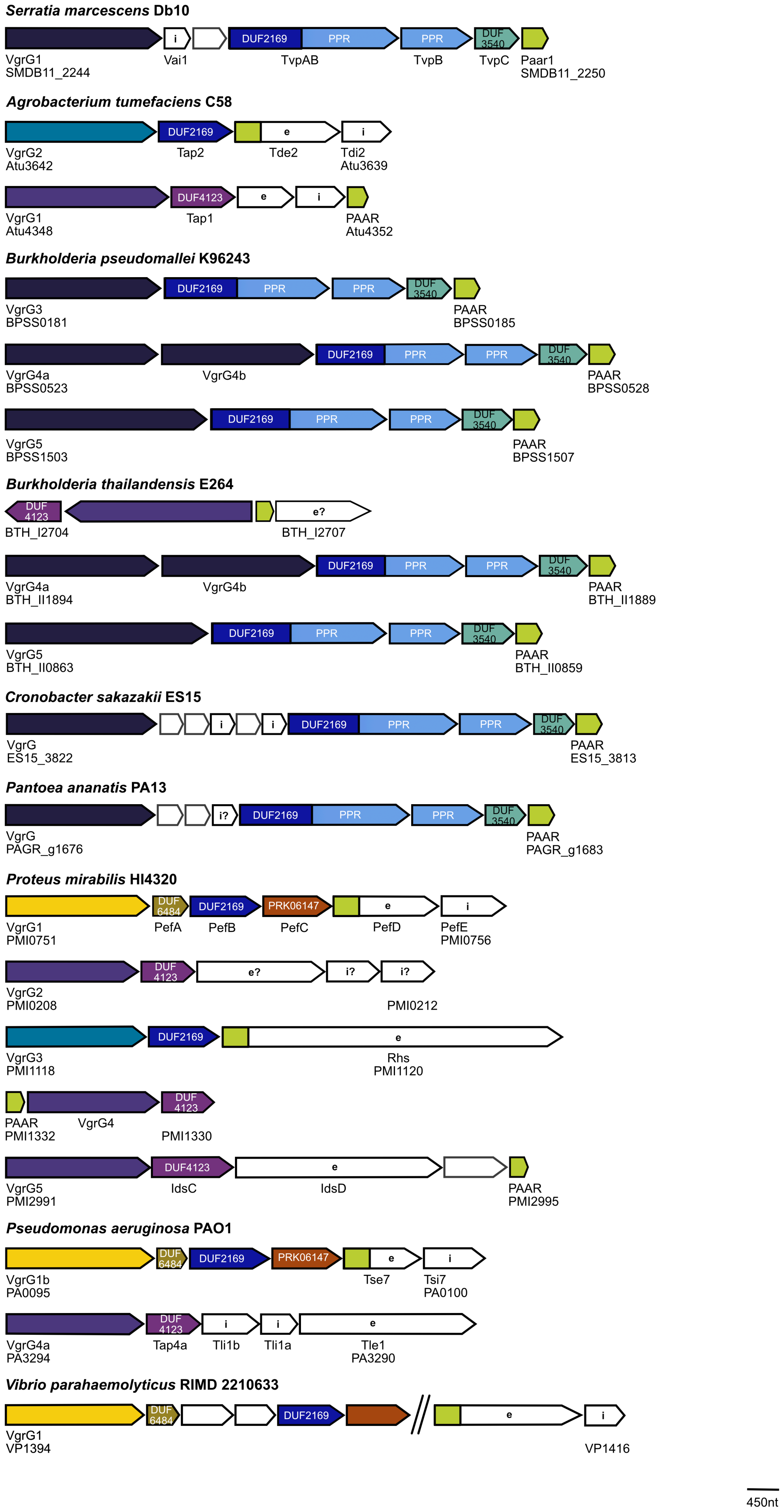

Appendix Figure S5

Appendix Figure S5. **Conservation of Tvp accessory proteins in representative Gram-negative species.** The genetic context of T6SS-associated DUF2169-containing accessory proteins in *Serratia marcescens* Db10 (accession: HG326223), *Agrobacterium tumefaciens* C58 (accession: AE007869.2), *Pantoea ananatis* PA13 (accession: CP003085.1), *Cronobacter sakazakii* ES15 (accession: AFK01427), *Proteus mirabilis* HI4320 (accession: NC_010554.1), *Pseudomonas aeruginosa* PAO1 (accession: AE004091.2), *Burkholderia pseudomallei* K96243 (accession: NC_006350.1 and NC_006351.1) and *B. thailandensis* E264 (accession: CP000085.1 and CP000086.1). If present in these genomes, the genetic context of DUF4123-containing accessory proteins is also depicted. Each open reading frame (ORF) is represented by an arrow whose size is proportional to its gene length. The name and/or the genomic identifier of the ORFs at each end is indicated below each set of genes. When identified, conserved domains (PPR for pentapeptide repeat, DUF3540, DUF6484, PRK06147) are also highlighted inside the ORFs as well as effector (e) and immunity (i) encoding genes.

Appendix Figure S6

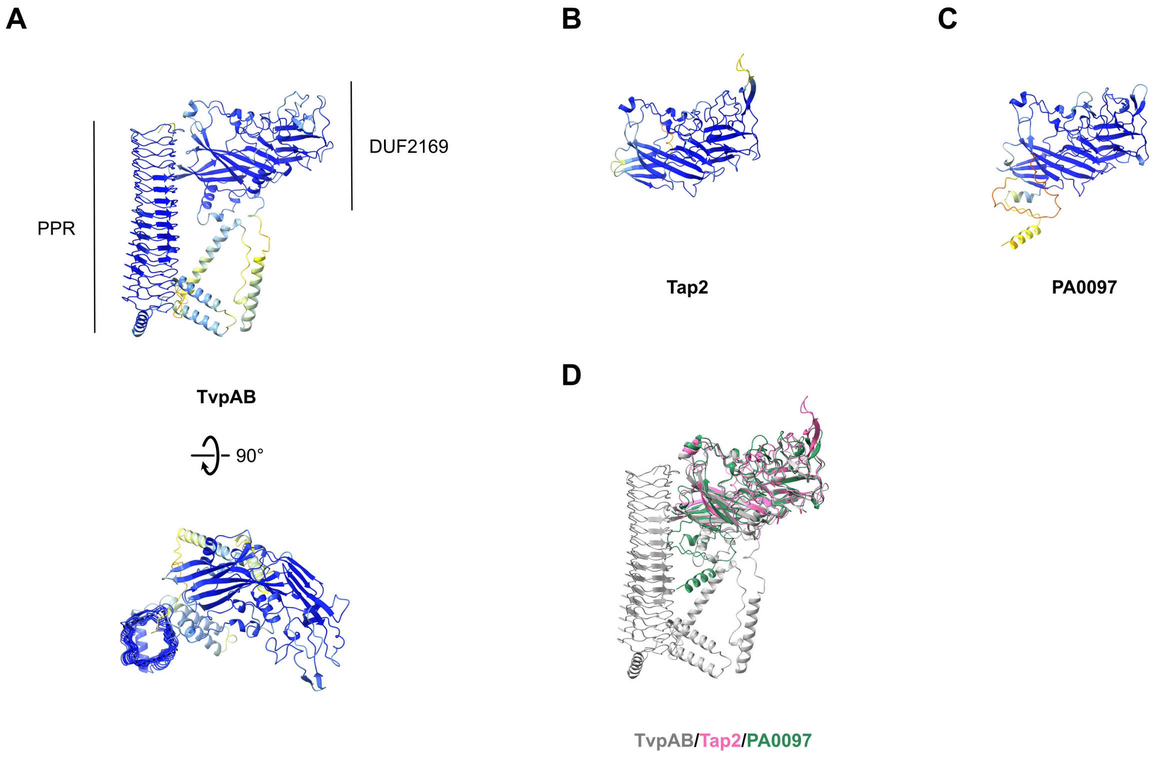

Appendix Figure S6. Predicted structures of TvpAB, Tap2 and PA0097 proteins. (A) Ribbon representation of the predicted structure of TvpAB from *S. marcescens* Db10. The DUF2169 and pentapeptide repeat (PPR) domains are highlighted. A 90° rotated view is provided to show the quadrilateral shape of the PPR β-helix. **(B)** Ribbon representation of the predicted structure of the *Agrobacterium tumefaciens* DUF2169-containing accessory protein Tap2. **(C)** Ribbon representation of the predicted structure of the *Pseudomonas aeruginosa* DUF2169-containing accessory protein PA0097. **(D)** Superposition of TvpAB (grey), Tap2 (pink) and PA0097 (green) predicted structures. **(A-C)** Predicted structures were generated by AlphaFold2 and coloured according to the pLDDT score, from red (pLDDT <50, lowest confidence) to blue (pLDDT >90, highest confidence).

Appendix Figure S7

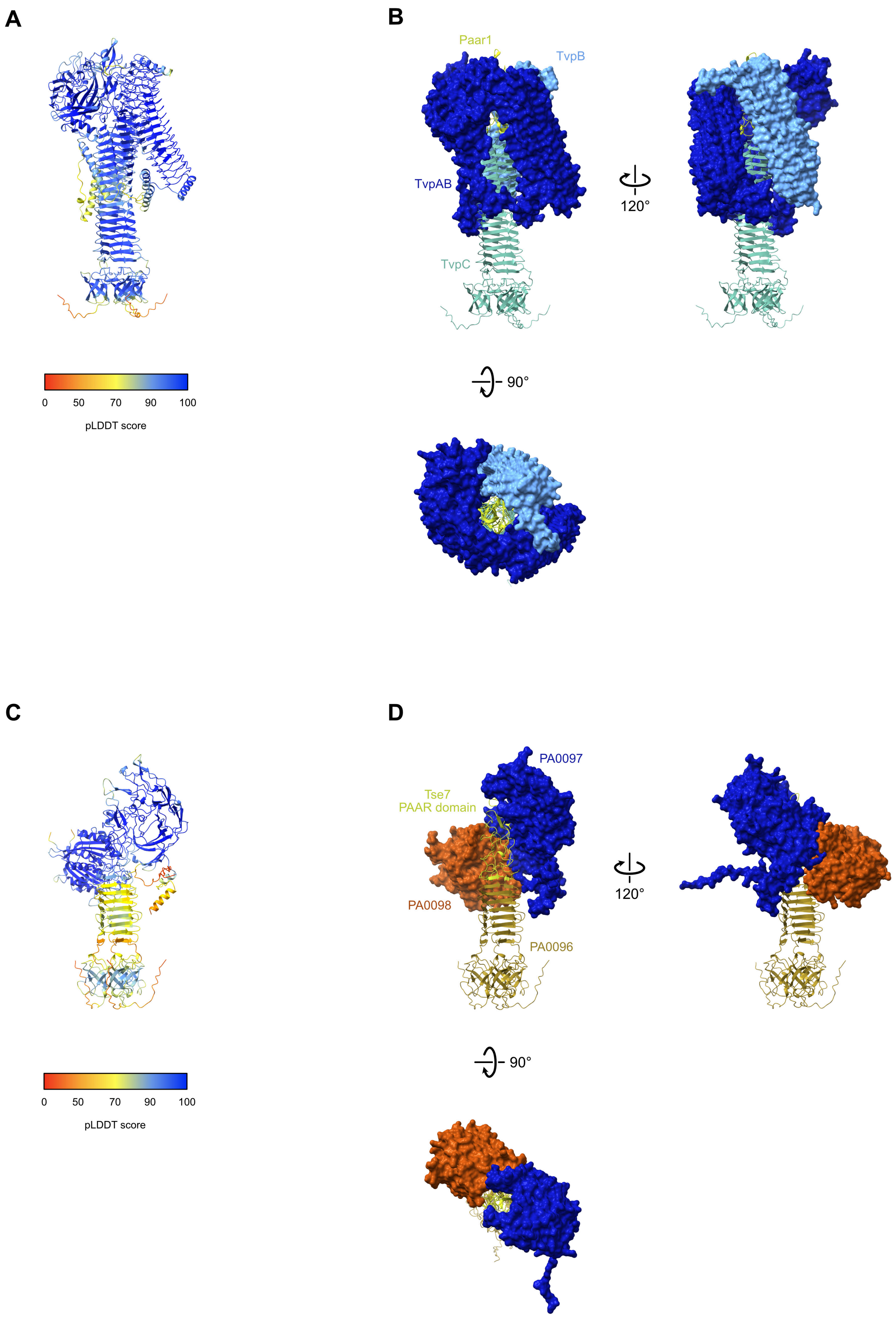

Appendix Figure S7. **Predicted structures of *Serratia marcescens* and *Pseudomonas aeruginosa* accessory pre-complexes. (A)** Ribbon representation of the *Serratia marcescens* Tvp accessory pre-complex coloured according to the pLDDT score from AlphaFold2 modelling, from red (pLDDT <50, lowest confidence) to blue (pLDDT >90, highest confidence). **(B)** The *S. marcescens* Tvp accessory pre-complex, as in Figure 6a, with the surface of TvpAB and TvpB highlighted to show how the proteins wrap the tip of the pre-complex. **(C)** Ribbon

Appendix Figure S7

representation of the *Pseudomonas aeruginosa* accessory pre-complex coloured according to the pLDDT score from AlphaFold2 modelling, from red (pLDDT <50, lowest confidence) to blue (pLDDT >90, highest confidence). **(D)** The *P. aeruginosa* accessory pre-complex, as in Figure 6B, with the surface of PA0097 and PA0098 highlighted to show how the proteins wrap the tip of the pre-complex.

Appendix Figure S8

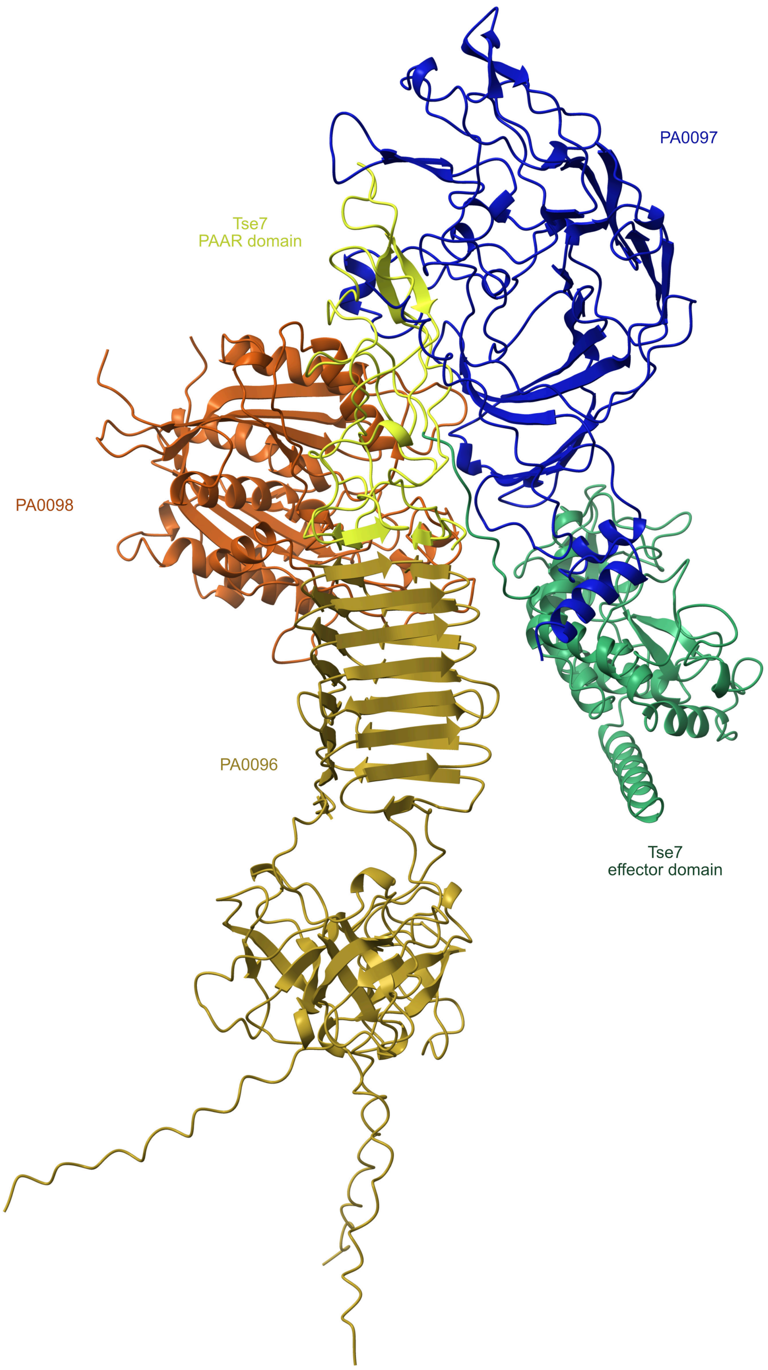

Appendix Figure S8. Predicted structure of the *Pseudomonas aeruginosa* accessory pre-complex with the full Tse7 effector. Ribbon representation of the predicted structure of the *P. aeruginosa* accessory pre-complex generated with AlphaFold2. PA0096 is colored in ginger, PA0097 in medium blue, PA0098 in rust, the PAAR domain of Tse7 effector in light green and the effector domain of Tse7 in sea green

Appendix Figure S9

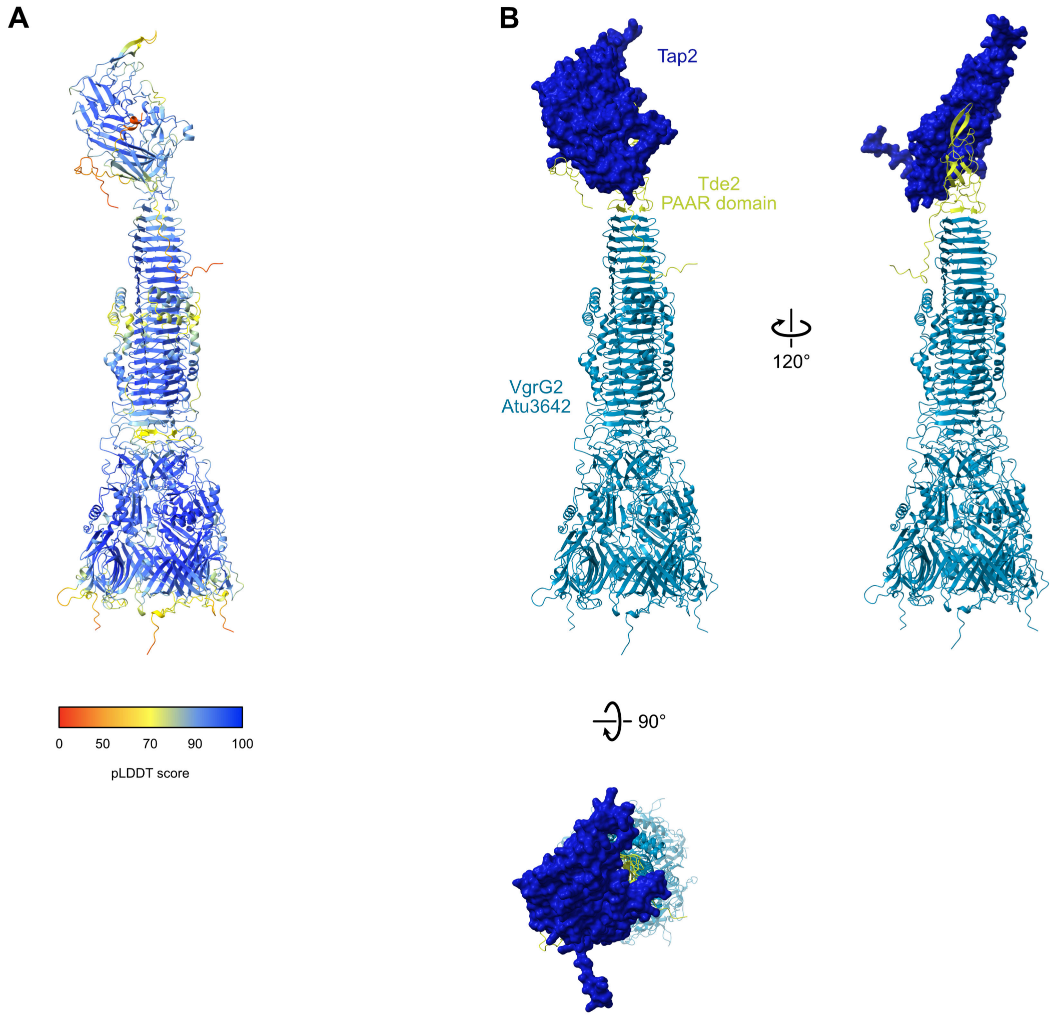

Appendix Figure S9. Predicted structure of the *Agrobacterium tumefaciens* VgrG2-associated accessory complex. (A) Ribbon representation of the *Agrobacterium tumefaciens* VgrG2-associated accessory complex coloured according to the pLDDT score from AlphaFold2 modelling, from red (pLDDT <50, lowest confidence) to blue (pLDDT >90, highest confidence). **(B)** The *A. tumefaciens* VgrG2-associated accessory complex, as in Figure 6E, with the surface of Tap2 highlighted to show how the protein wraps the tip of the complex.

Appendix Figure S10

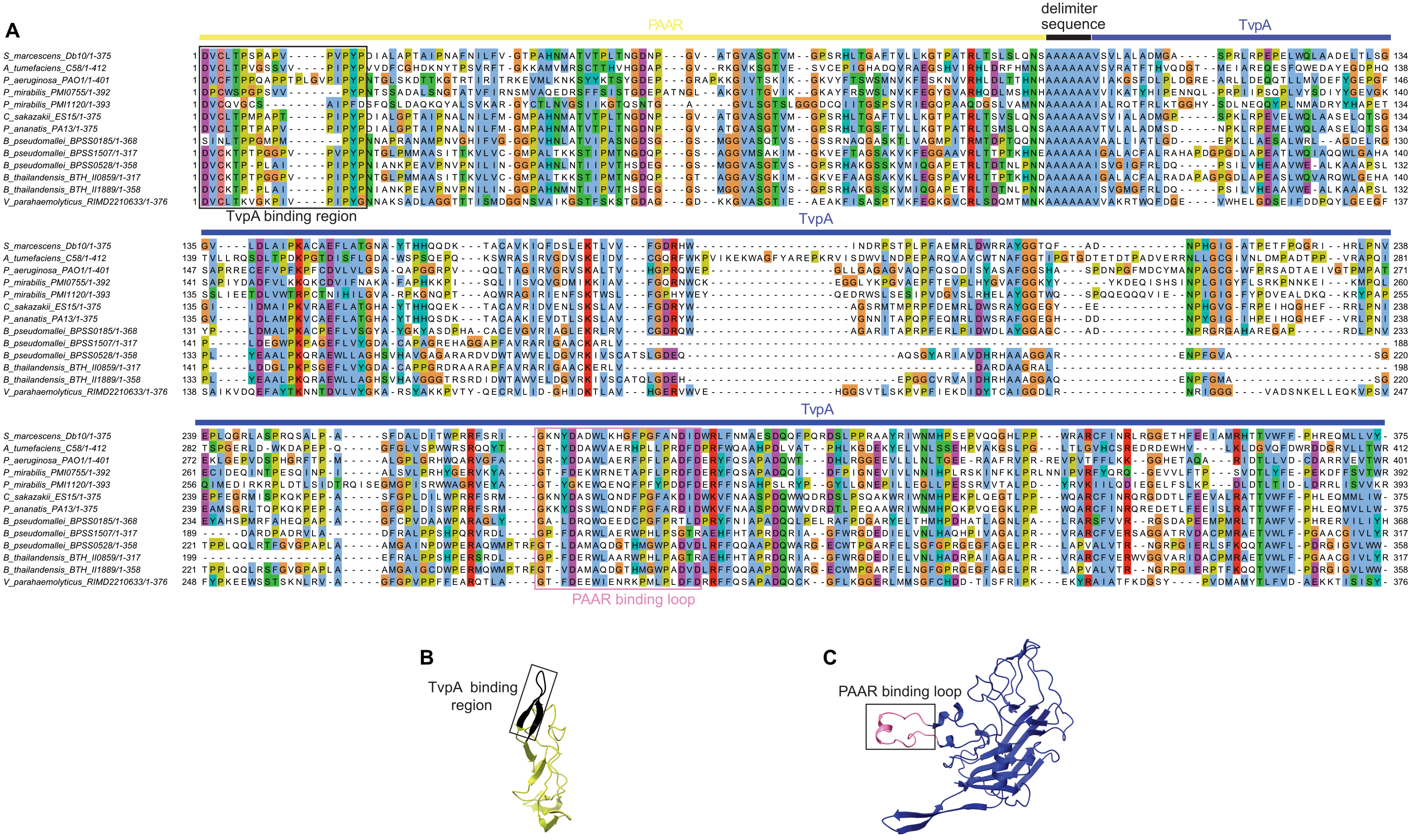

Appendix Figure S10

Appendix Figure S10. Identification of conserved regions of the TvpA and PAAR DUF4150 proteins for phylogenetic analysis of the Tvp system. (A) A concatenated PAAR-TvpA sequence was used to generate a multiple sequence alignment using the MUSCLE algorithm (Edgar, 2004) and coloured using ClustalX (Thompson, 1997). The alignment was also used for the purpose of phylogenetic tree construction. The chosen sequences were validated against structural comparisons between the corresponding AlphaFold3 predicted structures and each represents a conserved structural region. The conserved region is represented by **(B)** PAAR amino acids 16-110 (shown in green-yellow) and **(C)** TvpA amino acids 26-299 (shown in medium-blue) both from *S. marcescens* Db10. Highlighted within these regions is the conserved TvpA binding interface of PAAR (black box in A, black ribbon in B) and the corresponding PAAR binding loop from TvpA (pink box in A, pink ribbon in B). Ribbon representations of structures in B) and C) were generated in ChimeraX (Meng *et al*, 2023).

Appendix Figure S11

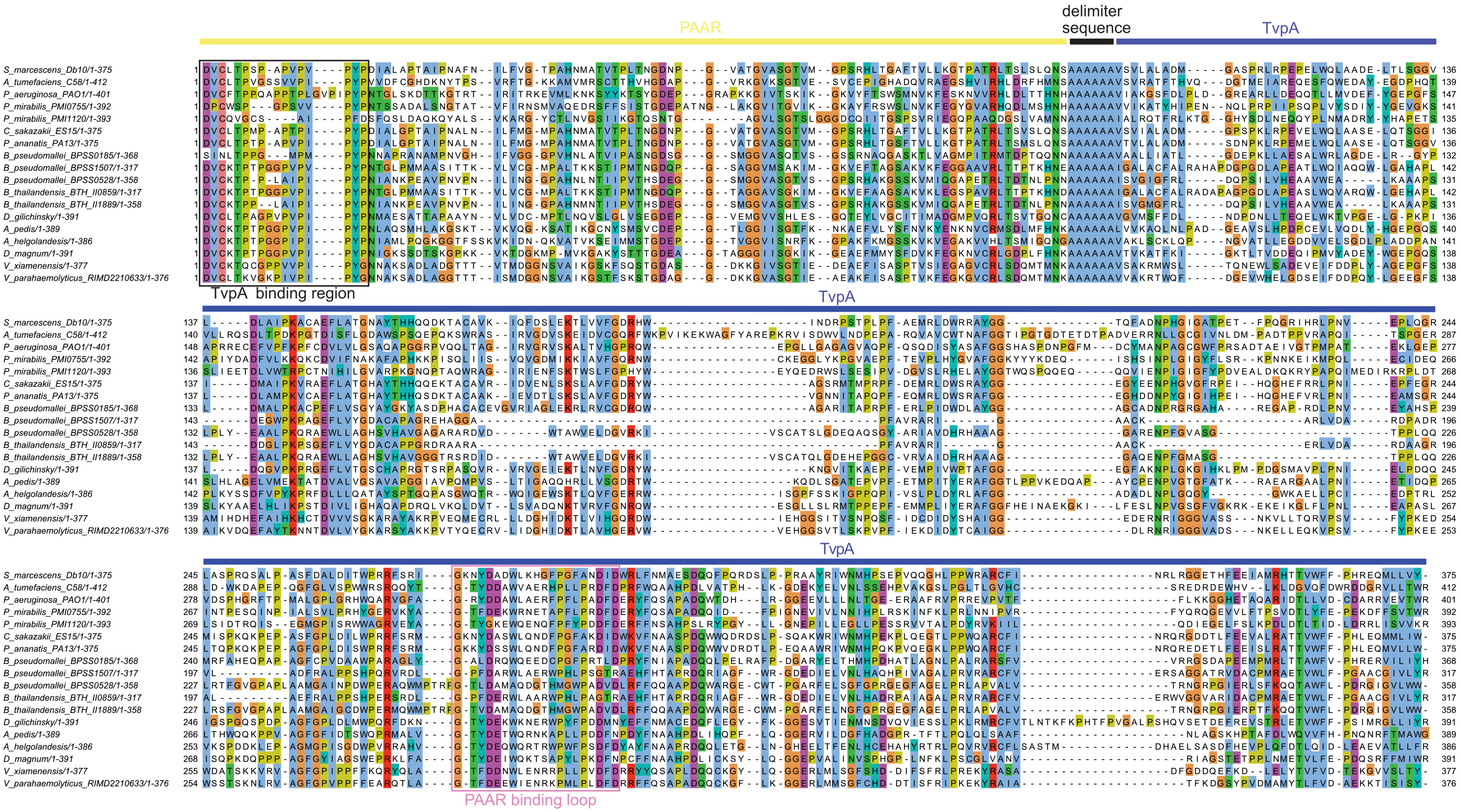

Appendix Figure S11. Alignment of an extended selection of PAAR, TvpA sequences for phylogenetic analysis. As for Appendix Figure S10, except that an expanded set of concatenated PAAR-TvpA sequences was selected to include bacterial species from additional phyla.

Appendix Figure S12

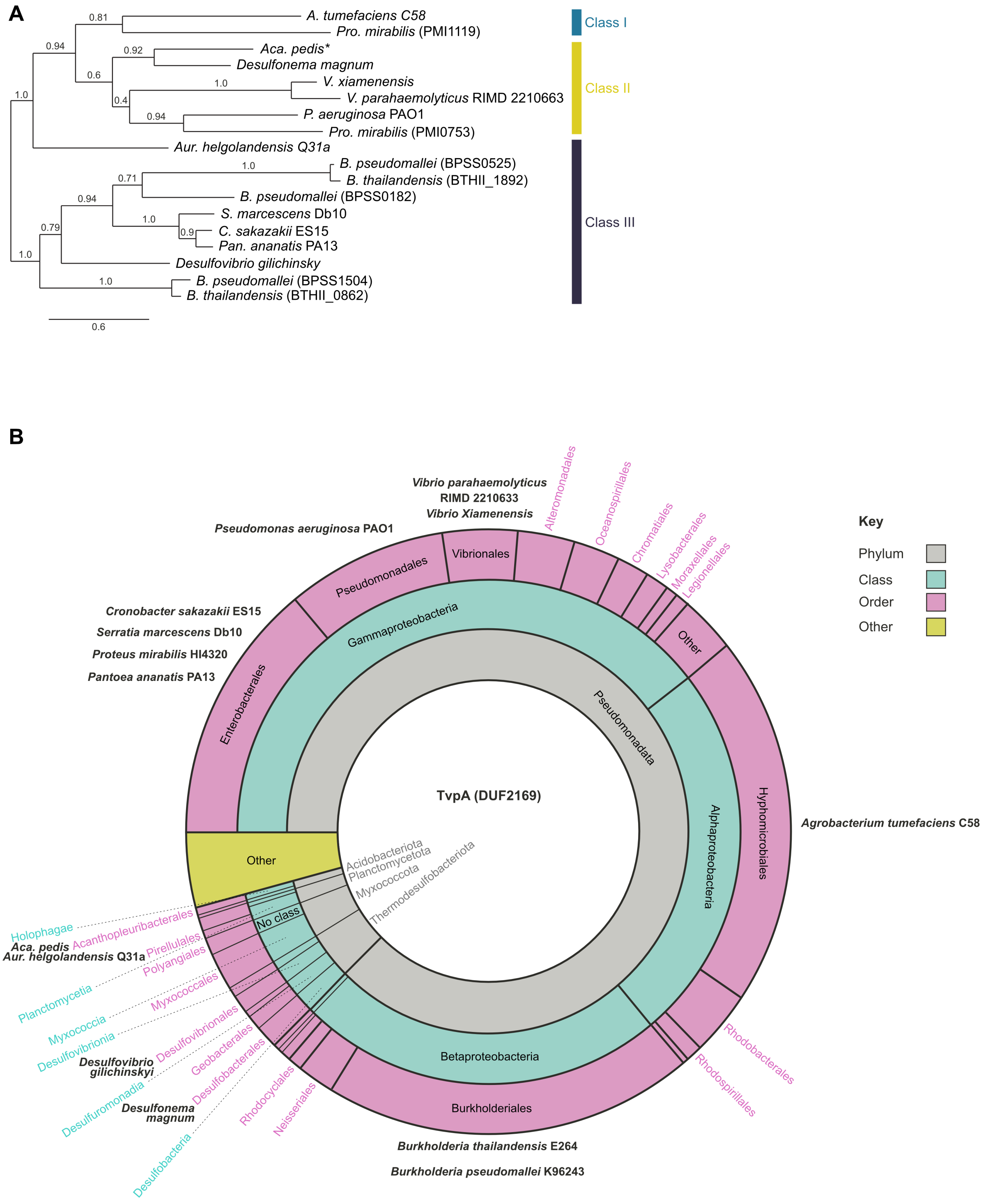

Appendix Figure S12. Extended phylogeny and taxonomy. **(A)** An extended phylogenetic tree, similar to that in Figure 8A, was constructed from the expanded set of sequences in the extended sequence alignment in Appendix Figure S11. This represents an extension from the *Pseudomonodata* phylum to include representatives from additional phyla (Dereeper *et al*, 2008). Where strains contain multiple Tvp systems, the accession number for the TvpA protein is enclosed in brackets to distinguish between them. *No syntenous TvpC protein. **(B)** Taxonomic distribution of TvpA (DUF2169) genes (InterPro entry: IPR018683; Pfam: PF09937) (Mistry *et al*, 2021; Blum *et al*, 2025) as in Figure 8D with the positions of *Acanthopleuribacter pedis (Acidobacteriota)*, *Aureliella helgolandensis (Planctomycetota)*, *Desulfonema magnum* and *Desulfovibrio gilichinskyi (Thermodesulfobacteriota)* indicated.

Appendix Figure S13

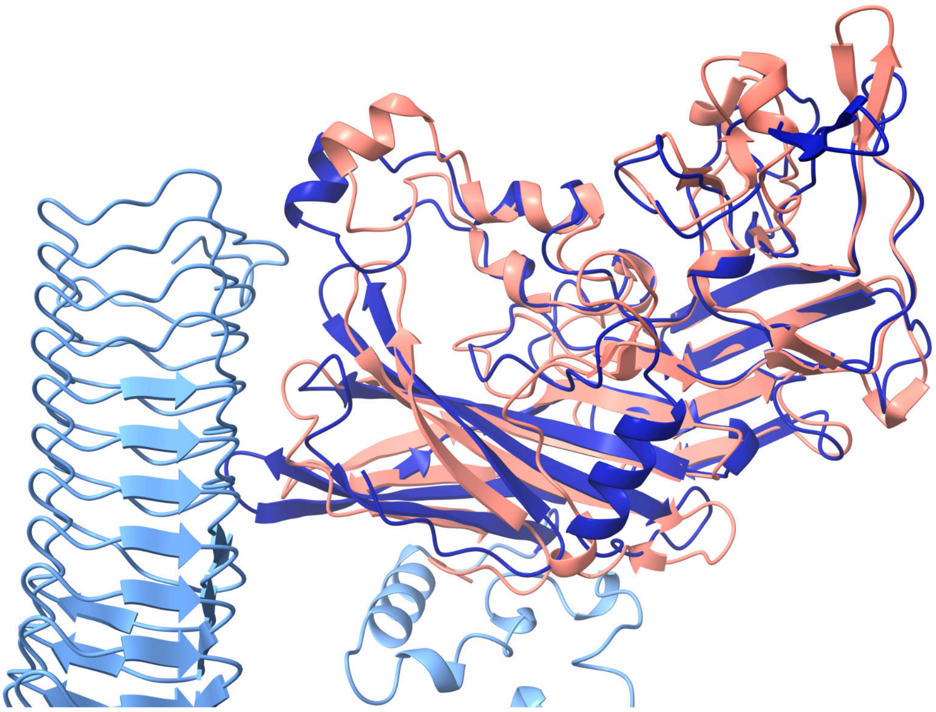

Appendix Figure S13. **Comparison of the Alphafold3 predicted structure of TvpAB from *Serratia marcescens* Db10 with the experimentally-determined structure of TvpA of *Vibrio xiamenensis*.** Ribbon representations of the AlphaFold3 model of TvpAB from *S. marcescens* and the crystal structure of *V. xiamenensis* TvpA (PDB: 8VTH). Superimposition of the structures was performed using ChimeraX (Meng *et al*, 2023) (RMSD = 1.076 Å between 140 pruned atom pairs) and the figure focuses on the DUF2169 domain. The structure of the TvpA domain of Db10 (amino acids 1-323) is shown in dark blue while the rest of the structure is shown in light blue. The *V. xiamenensis* TvpA protein is shown in salmon.

Appendix Figure S14

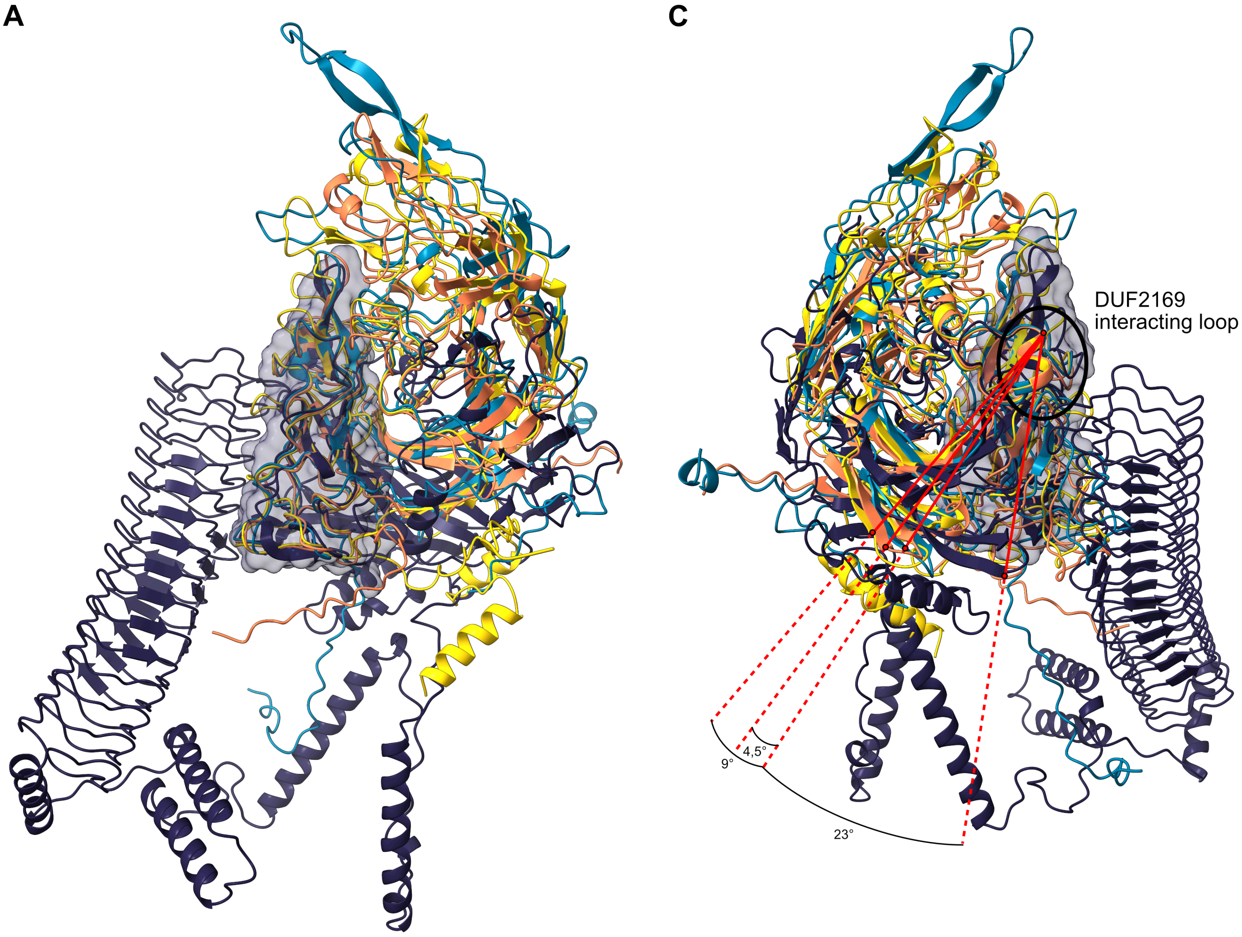

B

Orientation compared to class I *A. tumefaciens* TvpA-PAAR complex			
		Rotation	Shift
Class II	*P. aeruginosa* PAO1	3.6°	0.1 Å
	V. parahaemolyticus RMID 2210633	11.7°	1.4 Å
Class III	*S. marcescens* Db10	43.6°	-0.2 Å

Appendix Figure S14. Comparison of the orientation of the DUF2169 domain in the TvpA-PAAR complex between the different classes of Tvp systems. (A) AlphaFold3 structural predictions of TvpAB-Paar1 from *S. marcescens* Db10 (dark blue), Tap2-PAAR domain of Tde2 from *A. tumefaciens* C58 (cyan), PA0097-PAAR domain of Tse7 from *P. aeruginosa* PAO1 (yellow), and VP1398-PAAR domain of VP1415 from *V. parahaemolyticus* RIMD 2210633 (salmon) are represented as cartoons and superimposed by their PAAR domain. The surface of Db10 Paar1 is highlighted. **(B)** Table summarizing the rotation and shift required to realign the DUF2169 domain of all the TvpA proteins to the DUF2169 domain of Tap2 from *A. tumefaciens* starting from the superimposition presented in A). **(C)** Same structural predictions as in A) but superimposed by the short α-helix located in the interacting loop defined by Sachar *et al*. (Sachar *et al*, 2025). An axis was drawn between the top of this short α-helix and the end of the same β-strand for all the structures (red line). Flexibility of the DUF2169 domain among the different classes is estimated by the rotation angle formed between the axis of *A. tumefaciens* (Class I) and those of Class II (*P. aeruginosa* and *V. parahaemolyticus*) or Class III (*S. marcescens*)

Appendix Figure S15

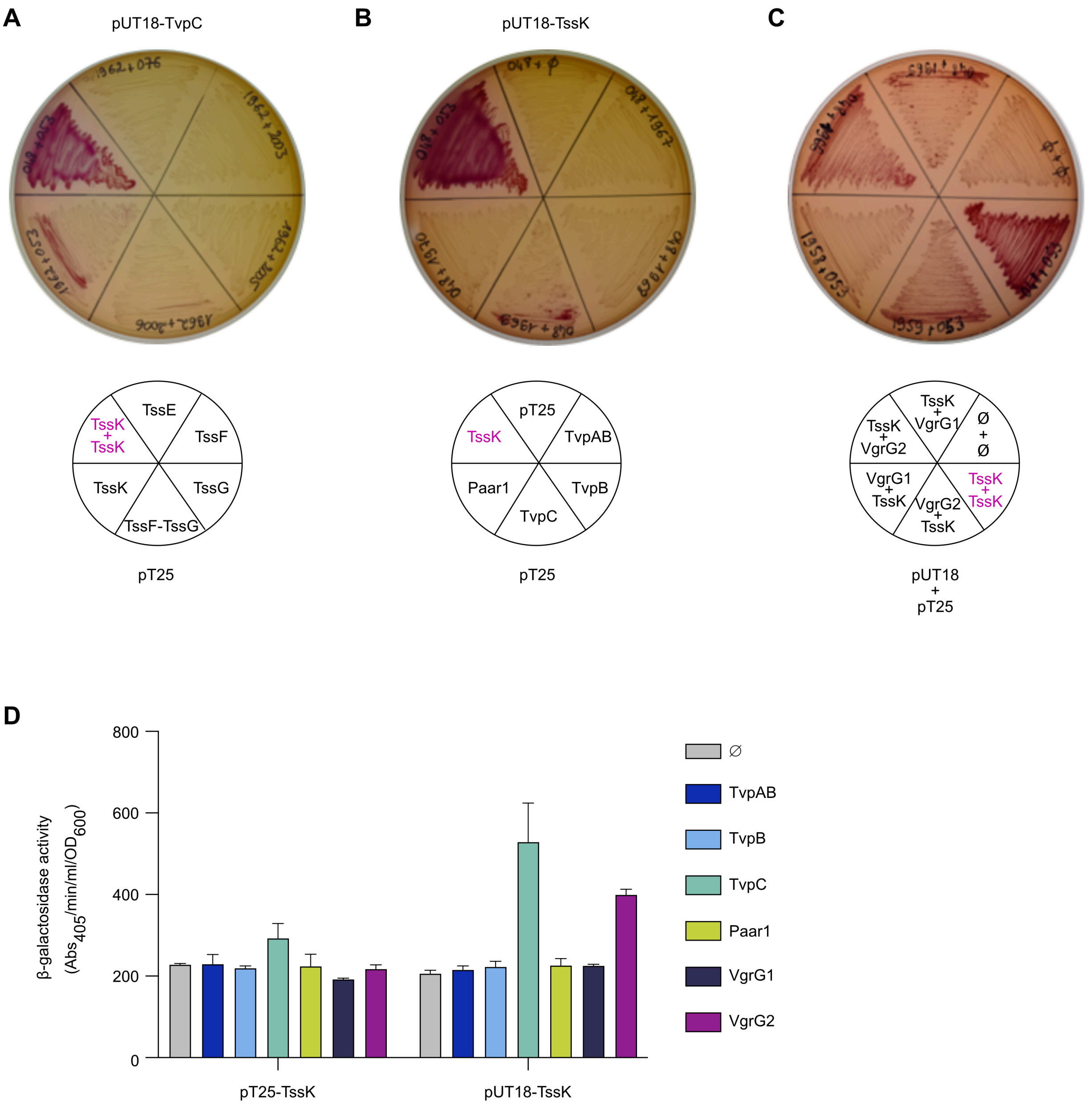

Appendix Figure S15. **Interaction between TssK and TvpC by bacterial-two-hybrid assay.**
A-C) Detection of interactions between T6SS components by observing growth of *E. coli* Δ*cya* carrying derivatives of the pUT18 and pT25 vectors directing the expression of T18 and T25 fusions with proteins of interest on MacConkey maltose plates. A red colour indicates positive interaction between proteins in the two-hybrid system. As a positive control, the interaction between TssK monomers was assayed, the negative control was empty pUT18 and pUT25 plasmids (Ø + Ø). **(A)** Testing the interaction between TvpC (in pUT18) and baseplate components (in pT25) from *S. marcescens* Db10 when expressed in *E. coli* Δ*cya*. **(B)** Testing the interaction between TssK (in pUT18) and Tvp accessory proteins (in pT25) from *S. marcescens* Db10 when expressed in *E. coli* Δ*cya*. **(C)** Testing the interaction between TssK and VgrG proteins from *S. marcescens* Db10 (either in pUT18 or pT25) when expressed in *E. coli* Δ*cya*. **(D)** Measurement of β-galactosidase activity to quantify the interactions between TssK and Tvp accessory proteins or VgrG proteins presented in A-C). Measurement of the absorbance at 405 nm was performed following incubation of toluene-permeabilized cells for 30 minutes at 37°C with ONPG substrate.

Appendix Table S1

Name	Description/Genotype	Source/reference
**Strains**		
Serratia marcescens Db10		
Db10	Wild-type	(Flyg *et al*, 1980)
SJC11	Δ*tssE* (*SMDB11_2271*)	(Murdoch *et al*, 2011)
FRA01	Δ*vgrG2* (*SMDB11_2276*)	(Cianfanelli *et al*, 2016)
FRA02	Δ*vgrG1* (*SMDB11_2244*)	(Cianfanelli *et al*, 2016)
FRA03	Δ*vgrG1* Δ*vgrG2*	(Cianfanelli *et al*, 2016)
DWL05	Δ*vai1* (*SMDB11_2245)*	This study
DP10	Δ*SMDB11_2246*	This study
DP09	Δ*vai1* Δ*tssE*	This study
DP15	Δ*vai1* Δ*tssE* Sm-resistant derivative	This study
DP13	Δ*SMDB11_2246* Δ*tssE*	This study
DP16	Δ*SMDB11_2246* Δ*tssE* Sm-resistant derivative	This study
DWL03	Δ*vai1Δ2246* Δ*tssE*	This study
DWL08	Δ*vai1Δ2246* Δ*tssE* Sm-resistant derivative	This study
KT13	Δ*SMDB11_2244-2281*	(English *et al*, 2012)
LM32	Δ*vgrG2* Δ*SMDB11_2246*	This study
KK1	Δ*tvpAB* (*SMDB11_2247*)	This study
DP11	Δ*tvpB* (*SMDB11_2248*)	This study
DWL04	Δ*tvpC* (*SMDB11_2249*)	This study
FRC32	Δ*paar1* (*SMDB11_2250*)	(Cianfanelli *et al*, 2016)
DP02	Δ*vgrG2* Δ*tvpAB*	This study
CE76	Δ*vgrG2* Δ*tvpB*	This study
DP03	Δ*vgrG2* Δ*tvpC*	This study
FRC26	Δ*vgrG2* Δ*paar1*	(Cianfanelli *et al*, 2016)
LM33	Δ*vgrG1* Δ*tvpAB*	This study
YL37	Db10 Δ9 [Δ*ssp1* (Δ*SMDB11_2261*), Δ*ssp2* (Δ*SMDB11_2264*), Δ*ssp3/tfe1* (Δ*SMDB11_1112*), Δ*ssp4* (Δ*SMDB11_3980*), Δ*ssp5* (Δ*SMDB11_4628*), Δ*ssp6* (Δ*SMDB11_4673*), Δ*rhs1* (Δ*SMDB11_2278*), *rhs2*$_{H1369A}$ (*SMDB11_1610*$_{H1369A}$), Δ*slp* (Δ*SMDB11_0927*)]	(Reglinski *et al*, 2025)
GM131	Δ9 Δ*vgrG1*	This study
CE030	Δ9 Δ*vgrG2*	This study
CE036	*vai1*-3xFlag (encodes *vai1* with a C-terminal 3xFLAG tag at native *vai1* locus in a wild-type background)	This study
CE043	*vai1*-3xFlag Sm-resistant derivative	This study
CE049	Δ*vgrG2* SMDB11_2245-3xFlag (encodes *vai1* with a C-terminal 3xFLAG tag at native *vai1* locus in a Δ*vgrG2* background)	This study
JAD09	Δ*rhsI1* Δ*tssH* Sm-resistant derivative	(Alcoforado Diniz & Coulthurst, 2015)
AO01/JAD06	Δ*sip4* Δ*ssp4* Sm-resistant derivative	(Cianfanelli *et al*, 2016)
LM26	His-VgrG1 (encodes VgrG1 with an N-terminal His$_6$ tag in a wild-type background)	This study
LM66	His-VgrG1 Δ*tvpAB* (encodes VgrG1 with an N-terminal His$_6$ tag in a Δ*tvpAB* background)	This study
CE56	His-VgrG1 Δ*tvpC* (encodes VgrG1 with an N-terminal His$_6$ tag in a Δ*tvpC* background)	This study
CE31	TvpAB-HA (encodes TvpAB with a C-terminal HA tag in a wild-type background)	This study

CE68	TvpAB-HA Δ*tvpC* (encodes TvpAB with a C-terminal HA tag in a Δ*tvpC* background)	This study
CE59	HA-TvpC (encodes TvpC with an N-terminal HA tag in a wild-type background)	This study
CE60	HA-TvpC Δ*vgrG2* (encodes TvpC with an N-terminal HA tag in a Δ*vgrG2* background)	This study
CE75	HA-TvpC Δ*vgrG1* (encodes TvpC with an N-terminal HA tag in a Δ*vgrG1* background)	This study
CE70	HA-TvpC Δ*tvpAB* (encodes TvpC with an N-terminal HA tag in a Δ*tvpAB* background)	This study
CE136	HA-TvpC$_{F138E,\ L152E,\ I159E}$ (encodes TvpC with an N-terminal HA tag and amino acid substitutions F138E, L152E, I159E)	This study
CE137	HA-TvpC $_{V210Q}$ (encodes TvpC with an N-terminal HA tag and amino acid substitution V210Q)	This study
CE138	HA-TvpC $_{D204R,\ D199R,\ E206R}$ (encodes TvpC with an N-terminal HA tag and amino acid substitutions D204R, D199R, E206R)	This study
CE141	HA-TvpC$_{D204R,\ D199R,\ E206R}$ Δ*vgrG2* (encodes TvpC with an N-terminal HA tag and amino acid substitutions D204R, D199R, E206R, in a Δ*vgrG2* background)	This study
CE142	HA-TvpC$_{V210Q}$ Δ*vgrG2* (encodes TvpC with an N-terminal HA tag and amino acid substitution V210Q, in a Δ*vgrG2* background)	This study
Pseudomonas fluorescens		
KT02	Sm-resistant derivative of *P. fluorescens 55*	(Murdoch *et al*, 2011)
Serratia marcescens SM39 (intrinsically Sm-resistant)		(Iguchi *et al*, 2014)
E. coli		
CC118λpir	Cloning host and donor strain for pKNG101-derived allelic exchange plasmids (λ*pir*)	(Herrero *et al*, 1990)
MG1655	Wild type (model K12 strain), used for heterologous toxicity assay	(Blattner *et al*, 1997)
MG1655 Δ*cyaA::apr*	Strain used for bacterial two hybrid assay	F. Sargent

Appendix Table S1. **List of strains used in this study**

Appendix Table S2

Name	Description/Genotype	Source/reference
pKNG101	Suicide vector for allelic exchange (Sm^R *sacBR mobRK2 oriR6K*)	(Kaniga *et al*, 1991)
pSC3048	Modified version of the recombineering, λ Red recombinase-encoding pORTMAGE plasmid. The dominant negative mutant *mutL* gene has been removed to prevent off-target effects due to suppression of DNA repair during integration of oligonucleotide CE75. The origin of replication from pBAD Kan-18 was introduced in place of the conjugation and replication machinery to allow for the stable maintenance of the plasmid in *S. marcescens* Db10 (Kn^R)	This study
pBAD18-Kn	Arabinose-inducible expression vector (Kn^R)	(Guzman *et al*, 1995)
pSUPROM	Vector for constitutive expression of cloned genes under the control of the *E. coli tat* promoter (Kn^R)	(Jack *et al*, 2004)
pUT18	Bacterial Two Hybrid plasmid (for fusion of protein of interest with C-terminal T18 fragment of CyaA; ApR)	(Karimova *et al*, 2000)
pT25	Bacterial Two Hybrid plasmid (for fusion of protein of interest with N-terminal T25 fragment of CyaA; CmR)	(Karimova *et al*, 1998)
pSC137	pBAD18 $SufI_{SP}$-Ssp2	This study
pSC138	pBAD18 $OmpA_{SP}$-Ssp2	(English *et al*, 2012)
pSC934	pBAD18 SMDB11_2246	This study
pSC942	pBAD18 $OmpA_{SP}$-SMDB11_2246	This study
pSC944	pBAD18 $OmpA_{SP}$-VgrG1	This study
pSC1940	pBAD18 VgrG1 CTD (598-786aa)	This study
pSC1941	pBAD18 $OmpA_{SP}$-VgrG1 CTD (598-786aa)	This study
pSC1943	pBAD18 VgrG1	This study
pSC1944	pBAD18 $OmpA_{SP}$-VgrG1 + SMDB11_2245	This study
pSC1945	pBAD18 $OmpA_{SP}$-VgrG1 CTD (598-786aa) + SMDB11_2245	This study
pSC1951	pBAD18 $SufI_{SP}$-VgrG1 CTD (598-786aa)	This study
pSC1914	pSUPROM *SMDB11_2245*	This study
pSC1915	pSUPROM *SMDB11_2246*	This study
pSC1916	pSUPROM *tvpAB* (*SMDB11_2247*)	This study
pSC1917	pSUPROM *tvpB* (*SMDB11_2248*)	This study
pSC1918	pSUPROM *tvpC* (*SMDB11_2249*)	This study
pSC1919	pSUPROM *paar1* (*SMDB11_2250*)	This study
pSC601	pKNG101-derived allelic exchange plasmid for the generation of chromosomal in-frame Δ*vgrG2* (Δ*SMDB11_2276*) deletion	(Cianfanelli *et al*, 2016)
pSC602	pKNG101-derived allelic exchange plasmid for the generation of chromosomal in-frame Δ*vgrG1* (Δ*SMDB11_2244*) deletion	(Cianfanelli *et al*, 2016)
pSC921	pKNG101-derived allelic exchange plasmid for the generation of chromosomal in-frame Δ*SMDB11_2245* deletion	This study
pSC937	pKNG101-derived allelic exchange plasmid for the generation of chromosomal in-frame Δ*SMDB11_2246* deletion	This study
pSC577	pKNG101-derived allelic exchange plasmid for the generation of chromosomal in-frame Δ*SMDB11_2245-2246* deletion	This study
pSC902	pKNG101-derived allelic exchange plasmid for the generation of chromosomal in-frame Δ*tvpAB* deletion (Δ*SMDB11_2247*)	This study
pSC945	pKNG101-derived allelic exchange plasmid for the generation of chromosomal in-frame Δ*tvpB* deletion (Δ*SMDB11_2248*)	This study
pSC923	pKNG101-derived allelic exchange plasmid for the generation of chromosomal in-frame Δ*tvpC* deletion (Δ*SMDB11_2249*)	This study

pSC3031	pKNG101-derived allelic exchange plasmid for chromosomal replacement of native *vai1* with gene encoding Vai1 with 3xFLAG (C-terminal tag)	This study
pSC2026	pKNG101-derived allelic exchange plasmid for chromosomal replacement of native *vgrG1* gene with gene encoding His-VgrG1 (N-terminal tag)	This study
pSC3027	pKNG101-derived allelic exchange plasmid for chromosomal replacement of native *tvpAB* gene with gene encoding TvpAB-HA (C-terminal tag)	This study
pSC3088	pKNG101-derived allelic exchange plasmid for chromosomal replacement of native *tvpC* gene with gene encoding HA-TvpC (N-terminal tag)	This study
pSC4407	pKNG101-derived allelic exchange plasmid for chromosomal replacement of native *tvpC* gene with gene encoding HA-TvpC with substitutions F138E L152E I159E	This study
pSC4408	pKNG101-derived allelic exchange plasmid for chromosomal replacement of native *tvpC* gene with gene encoding HA-TvpC with substitutions D204R D199R E206R	This study
pSC4409	pKNG101-derived allelic exchange plasmid for chromosomal replacement of native *tvpC* gene with gene encoding HA-TvpC with substitution V210Q	This study
pSC048	pUT18 *tssK* (*SMDB11_2253*)	(Cianfanelli *et al*, 2016)
pSC1962	pUT18 *tvpC* (*SMDB11_2249*)	This study
pSC1958	pUT18 *vgrG1* (*SMDB11_2244*)	This study
pSC1959	pUT18 *vgrG2* (*SMDB11_2276*)	This study
pSC053	pT25 *tssK* (*SMDB11_2253*)	(Cianfanelli *et al*, 2016)
pSC076	pT25 *tssE* (*SMDB11_2271*)	This study
pSC1967	pT25 *tvpAB* (*SMDB11_2247*)	This study
pSC1968	pT25 *tvpB* (*SMDB11_2248*)	This study
pSC1969	pT25 *tvpC* (*SMDB11_2249*)	This study
pSC1970	pT25 *paar1* (*SMDB11_2250*)	This study
pSC1965	pT25 *vgrG1* (*SMDB11_2244*)	This study
pSC1966	pT25 *vgrG2* (*SMDB11_2276*)	This study
pSC2003	pT25 *tssF* (*SMDB11_2272*)	This study
pSC2005	pT25 *tssG* (*SMDB11_2273*)	This study
pSC2006	pT25 *tssF-tssG* (*SMDB11_2272-73*)	This study

Appendix Table S2. List of plasmids used in this study.

Appendix Table S3

Plasmid	Sequence of relevant primers (5'-3')[a,b]	Description
pSC137	TATAGAGCTCAGAGGACGTTGATATGTCACTCAGTCGACGTCAG	Forward primer to clone SufI (SMDB11_3496) signal peptide and ribosome binding site (RBS) from Ssp2 into pBAD18-Kn (SacI)
	TATATCTAGACGTTCCGCTCGCTTCAG	Reverse primer to clone SufI (SMDB11_3496) signal peptide into pBAD18-Kn (XbaI)
	TATATCTAGAAGTCGCCCTTCATTCCAACAAG	Forward primer to clone Ssp2 without ATG into pBAD18-Kn (XbaI)
	TAGAGCATGCGGATTTTATTTTAGTAACCATATAGATGCCTCG	Reverse primer to clone Ssp2 into pBAD18-Kn (SphI)
pSC934	TATATCTAGACACCTTGCATAAGGAAGTGTCACG	Forward primer to clone SMDB11_2246 including RBS into pBAD18-Kn (XbaI)
	TATAGCATGCTCAAGAGGACAGTGCGTTGTC	Reverse primer to clone SMDB11_2246 into pBAD18-Kn (SphI)
pSC942	TATATCTAGAGGCGCGTTCATTGCCTTTTC	Forward primer to clone SMDB11_2246 into pBAD18-OmpA$_{SP}$-Kn by replacement of Ssp2 in pSC138 (XbaI)
	TATAGCATGCTCAAGAGGACAGTGCGTTGTC	Reverse primer to clone SMDB11_2246 into pBAD18-Kn (SphI)
pSC944	TATATCTAGAGATCGTCTTATCATTGCGCACAC	Forward primer to clone VgrG1 into pBAD18-OmpA$_{SP}$-Kn by replacement of Ssp2 (XbaI)
	TATAGCATGCTTACAAGAAGATCGCCAGCG	Reverse primer to clone VgrG1 into pBAD18-Kn (SphI)
pSC1940	TATAGAATTCCTAAAGCGGGGAATAGT*ATG*GCGCAGACGCAAAAC ATTAC	Forward primer to clone VgrG1 CTD (598-786aa) including VgrG1 RBS and ATG into pBAD18-Kn (EcoRI)
	TATAGCATGCTTACAAGAAGATCGCCAGCG	Reverse primer to clone VgrG1 CTD (598-786aa) into pBAD18-Kn (SphI)
pSC1941	TATATCTAGAGCGCAGACGCAAAACATTAC	Forward primer to clone VgrG1 CTD (598-786aa) into pBAD18-OmpA$_{SP}$-Kn by replacement of Ssp2 (XbaI)
	TATAGCATGCTTACAAGAAGATCGCCAGCG	Reverse primer to clone VgrG1 CTD (598-786aa) into pBAD18-Kn (SphI)
pSC1943	TATAGAATTCCTAAAGCGGGGAATAGTATGGATCG	Forward primer to clone VgrG1 into pBAD18-Kn (EcoRI)
	TATAGCATGCTTACAAGAAGATCGCCAGCG	Reverse primer to clone VgrG1 into pBAD18-Kn (SphI)
pSC1944	TATATCTAGAGATCGTCTTATCATTGCGCACAC	Forward primer to clone VgrG1 into pBAD18-OmpA$_{SP}$-Kn by replacement of Ssp2 (XbaI)
	TATACTGCAGTCAAAGCCGGGAAAGCTCTTC	Reverse primer to clone VgrG1 with SMDB11_2245 into pBAD18-OmpA$_{SP}$-Kn by replacement of Ssp2 (PstI)
pSC1945	TATATCTAGAGCGCAGACGCAAAACATTAC	Forward primer to clone VgrG1 CTD (598-786aa) with SMDB11_2245 into pBAD18-OmpA$_{SP}$-Kn by replacement of Ssp2 (XbaI)
	TATACTGCAGTCAAAGCCGGGAAAGCTCTTC	Reverse primer to clone VgrG1 CTD (598-786aa) with SMDB11_2245 into pBAD18-OmpA$_{SP}$-Kn by replacement of Ssp2 (PstI)
pSC1951	TATATCTAGAGCGCAGACGCAAAACATTAC	Forward primer to clone VgrG1 CTD (598-786aa) into pBAD18-SufI$_{SP}$-Kn by replacement of Ssp2-Rap2ab in pSC143(XbaI)
	TATAGCATGCTTACAAGAAGATCGCCAGCG	Reverse primer to clone VgrG1 CTD (598-786aa) into pBAD18-Kn (SphI)
pSC1914	TATAGGATCCATGTCGAAGGCCAAAAATGTTTTCA	Forward primer to clone SMDB11_2245 into pSUPROM (BamHI)
	TATATCTAGATCAAAGCCGGGAAAGCTCTTCGATC	Reverse primer to clone SMDB11_2245 into pSUPROM (XbaI)
pSC1915	TATAGGATCCATGGGCGCGTTCATTGCCTTTTCGA	Forward primer to clone SMDB11_2246 into pSUPROM (BamHI)
	TATATCTAGATCAAGAGGACAGTGCGTTGTCCTTC	Reverse primer to clone SMDB11_2246 into pSUPROM (XbaI)

Appendix Table S3

pSC1916	TATAGGATCCATGAAAGTCGTAAAACCTCTGCGCC	Forward primer to clone *tvpAB* (SMDB11_2247) into pSUPROM (BamHI)
	TATATCTAGATCATACTTCCTTCCCGTTGTGGCGA	Reverse primer to clone *tvpAB* (SMDB11_2247) in pSUPROM (XbaI)
pSC1917	TATATCTAGAATGACCTCAAAACCAGAGCAGATAC	Forward primer to clone *tvpB* (SMDB11_2248) in pSUPROM **(**XbaI**)**
	TATAAAGCTTTCAAATTCGGCTCTGGCGCTGTGCG	Reverse primer to clone *tvpB* (SMDB11_2248) in pSUPROM (HindIII)
pSC1918	TATAGGATCCATGAGCGTTGCCAACACAACTCGCG	Forward primer to clone *tvpC* (SMDB11_2249) in pSUPROM (BamHI)
	TATATCTAGATTATCCGACGTGGATTTGTTCTGAA	Reverse primer to clone *tvpC* (SMDB11_2249) in pSUPROM (XbaI)
pSC1919	TATAGGATCCATGTTTGCCAACAGCCAAATGATCG	Forward primer to clone *paar1* (SMDB11_2250) in pSUPROM (BamHI)
	TATATCTAGATCAGGGCGCAAGCAGCAACACTTTT	Reverse primer to clone *paar1* (SMDB11_2250) in pSUPROM (XbaI)
pSC1962	TATAAAGCTTGTAAAGAGGAGGAGTA**ATGAGCGTTGCCAACACAAC**	Forward primer to clone *tvpC* (SMDB11_2249) in pUT18 (HindIII)
	TATATCTAGAGA**TCCGACGTGGATTTGTTCTG**	Reverse primer to clone *tvpC* (SMDB11_2249) without stop in pUT18 (XbaI)
pSC1958	TATACTGCAGGTAAAGAGGAGGAGTA**ATGGATCGTCTTATCATTGC**	Forward primer to clone *vgrG1* (SMDB11_2244) in pUT18 (PstI)
	TATATCTAGAGA**CAAGAAGATCGCCAGCGCCT**	Reverse primer to clone *vgrG1* (SMDB11_2244) without stop in pUT18 (XbaI)
pSC1959	TATAAAGCTTGTAAAGAGGAGGAGTA**ATGTTGGACCGCATTATTGC**	Forward primer to clone *vgrG2* (SMDB11_2276) in pUT18 (HindIII)
	TATATCTAGAGA**GGATTTCGGGAACAGCTGAT**	Reverse primer to clone *vgrG2* (SMDB11_2276) without stop in pUT18 (XbaI)
pSC076	TATAGGATCCA**ATGAACGATAAACGCCCACC**	Forward primer to clone *tssE* (SMDB11_2271) in pT25 (BamHI)
	TATAGGTACC**TTACCCTATGTCTTTCAGATCGAAGTG**	Reverse primer to clone *tssE* (SMDB11_2271) in pT25 (KpnI)
pSC1967	TATAGGATCCG**AAAGTCGTAAAACCTCTGCG**	Forward primer to clone *tvpAB* (SMDB11_2247) without start in pT25 (BamHI)
	TATACCCGGG**TCATACTTCCTTCCCGTTGT**	Reverse primer to clone *tvpAB* (SMDB11_2247) in pT25 (SmaI)
pSC1968	TATACTGCAGGA**ACCTCAAAACCAGAGCAGAT**	Forward primer to clone *tvpB* (SMDB11_2248) without start in pT25 (PstI)
	TATAGGTACC**TCAAATTCGGCTCTGGCGCT**	Reverse primer to clone *tvpB* (SMDB11_2248) in pT25 (KpnI)
pSC1969	TATAGGATCCG**AGCGTTGCCAACACAACTCG**	Forward primer to clone *tvpC* (SMDB11_2249) without start in pT25 (BamHI)
	TATACCCGGG**TTATCCGACGTGGATTTGTT**	Reverse primer to clone *tvpC* (SMDB11_2249) in pT25 (SmaI)
pSC1970	TATAGGATCCG**TTTGCCAACAGCCAAATGAT**	Forward primer to clone *paar1* (SMDB11_2250) without start in pT25 (BamHI)
	TATACCCGGG**TCAGGGCGCAAGCAGCAACA**	Reverse primer to clone *paar1* (SMDB11_2250) in pT25 (SmaI)
pSC1965	TATACTGCAGGA**GATCGTCTTATCATTGCGCA**	Forward primer to clone *vgrG1* (SMDB11_2244) without start in pT25 (PstI)
	TATAGGTACC**TTACAAGAAGATCGCCAGCG**	Reverse primer to clone *vgrG1* (SMDB11_2244) in pT25 (KpnI)
pSC1966	TATAAGATCTG**TTGGACCGCATTATTGCGC**	Forward primer to clone *vgrG2* (SMDB11_2276) without start in pT25 (BglII)
	TATAGATATC**TCAGGATTTCGGGAACAGCT**	Reverse primer to clone *vgrG2* (SMDB11_2276) in pT25 (EcoRV)

Appendix Table S3

pSC2003	TATAGGATCCG**GACAGTAAACTGTTAGATTA**	Forward primer to clone *tssF* (SMDB11_2272) without start in pT25 (BamHI)
	TATACCCGGG**TCATATCAGCGTCCTTTTGC**	Reverse primer to clone *tssF* (SMDB11_2272) in pT25 (SmaI)
pSC2005	TATAGGATCCG**AACGCGGAGATGATCGCCAC**	Forward primer to clone *tssG* (SMDB11_2273) without start in pT25 (BamHI)
	TATACCCGGG**TCAGAACGGGTTTTCCAGCG**	Reverse primer to clone *tssG* (SMDB11_2273) in pT25 (SmaI)
pSC2006	TATAGGATCCG**GACAGTAAACTGTTAGATTA**	Forward primer to clone *tssF-tssG* (SMDB11_2272-73) without start for *tssF* in pT25 (BamHI)
	TATACCCGGG**TCAGAACGGGTTTTCCAGCG**	Reverse primer to clone *tssF-tssG* (SMDB11_2272-73) in pT25 (SmaI)
pSC921	TATATCTAGAATCAGTCTTTCAAGCTTGACGG	Forward primer to clone upstream region of SMDB11_2245 for allelic exchange (XbaI)
	TATAACTAGTCGACATGGGAGTCCCTTTACAAG	Reverse primer to clone upstream region of SMDB11_2245 for allelic exchange (SpeI)
	TATAACTAGTGAGCTTTCCCGGCTTTGAG	Forward primer to clone downstream region of SMDB11_2245 for allelic exchange (SpeI)
	TATAGGGCCCACAGTGCGTTGTCCTTCCC	Reverse primer to clone downstream region of SMDB11_2245 for allelic exchange (ApaI)
pSC937	TATATCTAGACCAATTTCAAGCTCAGTAATGATCC	Forward primer to clone upstream region of SMDB11_2246 for allelic exchange (XbaI)
	TATAACTAGTGCCCATCGTGACACTTCC	Reverse primer to clone upstream region of SMDB11_2246 for allelic exchange (SpeI)
	TATAACTAGTAACGCACTGTCCTCTTGATCATCC	Forward primer to clone downstream region of SMDB11_2246 for allelic exchange (SpeI)
	TATAGGGCCCGGATTATCGGCGAACTGG	Reverse primer to clone downstream region of SMDB11_2246 for allelic exchange (ApaI)
pSC577	TATATCTAGAATCAGTCTTTCAAGCTTGACGG	Forward primer to clone upstream region of SMDB11_2245 for allelic exchange (XbaI)
	TATAACTAGTCGACATGGGAGTCCCTTTACAAG	Reverse primer to clone upstream region of SMDB11_2245 for allelic exchange (SpeI)
	TATAACTAGTAACGCACTGTCCTCTTGATCATCC	Forward primer to clone downstream region of SMDB11_2246 for allelic exchange (SpeI)
	TATAGGGCCCGGATTATCGGCGAACTGG	Reverse primer to clone downstream region of SMDB11_2246 for allelic exchange (ApaI)
pSC902	TGTATCTAGACTGTTTACCGTCTTTGGCATCC	Forward primer to clone SMDB11_2247 upstream region for allelic exchange (XbaI)
	TGTAGGATCCGACTTTCATTGTGTGTGTAAAGCTTCC	Reverse primer to clone SMDB11_2247 upstream region for allelic exchange (BamHI)
	TATAGGATCCACATTGCCTCGCCACAACG	Forward primer to clone SMDB11_2247 downstream region for allelic exchange (BamHI)
	TATAGGGCCCAATTGGTTATCGGTAAACTGGC	Reverse primer to clone SMDB11_2247 downstream region for allelic exchange (ApaI)
pSC945	TATATCTAGACACGCTGGCCAACATTACC	Forward primer to clone upstream region of SMDB11_2248 for allelic exchange (XbaI)
	TATAACTAGTTGAGGTCATACTTCCTTCCCG	Reverse primer to clone upstream region of SMDB11_2248 for allelic exchange (SpeI)
	TATAACTAGTAGCCGAATTTGAATGCATTGAC	Forward primer to clone downstream region of SMDB11_2248 for allelic exchange (SpeI)
	TATAGGGCCCGTAATCTTCCGCCTGATAATCC	Reverse primer to clone downstream region of SMA2248 for allelic exchange (ApaI)
pSC923	TGTATCTAGAAGTTTGAAGGCGTGAACTTCG	Forward primer to clone upstream region of SMDB11_2249 for allelic exchange (XbaI)
	TATAACTAGTAACGCTCATGGTGATCCCCTC	Reverse primer to clone upstream region of SMDB11_2249 for allelic exchange (SpeI)

Appendix Table S3

	TATAACTAGTATCCACGTCGGATAACCTGC	Forward primer to clone downstream region of SMB11_2249 for allelic exchange (SpeI)
	TATAGGGCCCGTTGACTTCACTGCTGATG	Reverse primer to clone downstream region of SMDB11_2249 for allelic exchange (ApaI)
pSC2026	TATAAAGCTT**CATCATCATCATCATCAC**GATCGTCTTATCATTGCGCAC	Fwd primer to incorporate Nter **His Tag** on VgrG1 (SMDB11_2244) by allelic exchange (HindIII)
	TATAGTCGACGTCAGAAACGCTCCAGCTATTG	Reverse primer within VgrG1 coding region to incorporate Nter His Tag on VgrG1 (SMDB11_2244) by allelic exchange (SalI)
pSC3088	gggcccggtacccagctt	Forward primer for pBluescript to integrate HA-SMDB11_2249 encoding sequence at the *ApaI* site
	tctagagcggccgccacc	Reverse primer for pBluescript to integrate HA-SMDB11_2249 encoding sequence at the *XbaI* site
	gcggtggcggccgctctagaCAGTTTGAAGGCGTGAAC	Forward primer for upstream region of SMDB11_2249. Overlap with pBluescript shown as lower case.
	**TGCATAATCAGGAACATCATAAGGATA**CATGGTGATCCCCTCGTG	Reverse primer for upstream region of SMDB11_2249. HA tag encoding sequence shown in bold.
	**TATCCTTATGATGTTCCTGATTATGCA**AGCGTTGCCAACACAACTC	Forward primer for coding region of SMDB11_2249. HA tag encoding sequence shown in bold.
	aaaagctgggtaccgggcccGGTGATGTCTTTTGAGGTAATGAG	Reverse primer for coding region of SMDB11_2249. Overlap with pBluescript shown as lower case.
Primer CE75	T*T*CGAAACCGTTGGTCAAACGCACACGGCAAACTTTACGCAGTGCGGAGTTTGGTTTGTTCGGGGTAGTGGTATATACGCGGGTGCATA*C*G	Amplifies the SNP conferring streptomycin resistance from Db11 to introduce into target strains. Stars denote phosphothiorate bonds to increase stability and resistance to nucleases

**Plasmid**	**Details of synthetic insert**
pSC3031	Synthetic insert of DNA sequence encoding a C-terminal 3xFLAG tag (shown in **bold**) on Vai1 (SMDB11_2245) to be introduced into the native genomic locus by allelic exchange (containing *XbaI*/*ApaI* sites (underlined) for sub-cloning into pKNG101). Produced by GeneArt (ThermoFisher):<br>TCTAGACACACGATGCTGGATATCTCGCATAACCCGCTGTCGATCAGCAAAACCAATTTCAAGCTCAGTAATGATCCCATGACGTTCACTCAGCTTGGCG<br>CGCTGGTCACCAACGGTGCCTTACGATTGAGCACCAAGGCGCTGGCGATCTTCTTGTAAAGGGACTCCCATGTCGAAGGCCAAAAATGTTTTCACCGTT<br>ATCGGGTTGATTTTATTGGCTCCCATCCTCAGTCTGGTGGGTTTTTTCAAATTCATCACGGTCGATATCTGGGGTTATTTCAAACATATCGGTTCTTCCCT<br>CAGCGTGATGGAGAACGGTATTTCCGCTTCCGGGCAAATCGTCAGCATTCGCCAGACCAACCTGTGGGACGGAAATCGGCCGGTCTGCGAAGTCGAA<br>GTCAAGTACATCGCCCAAGACGGAAAAAACCATACGGCCGTGGCGAAAGGCCCGATCAGCGTCGTGGATCTGCCGCGTTATCAGCCGGGCCATTTCAC<br>CGCGATAAAGTACGATCCGAAGAACCCCAAAAAAGCGGTGATCGAAGAGCTTTCCCGGCTT**GACTACAAAGACCATGACGGTGATTATAAAGATCAT**<br>**GATATCGATTACAAGGATGACGATGACAAG**TGAGCGAAAAGGATCCCTTCAATTACGCGATCCGATTTCAACGTTCTGCAAACGGCTTATCGTTCATC<br>AGTTCGCTAAATCACCTTGCATAAGGAAGTGTCACGATGGGCGCGTTCATTGCCTTTTCGACGCAGGACAGCCTCTGGGGACAGATCCTGTTTACCGTC<br>TTTGGCATCCTGGTCGGCTGCGTATTGCTCGCTGCGATCGGCAAAACCTTTTTGTCGGCAAGCCGAGCGGCCGCCTTACCCATGCGGTTGCTGATCTTT<br>GTCGCGCTGATGCTGGCGCTCCTGAAATACAATCAGCAGGTGATGTCTGCGGTGAATGCTCCCGCAGAGCTGATCGGGTTCCTGATTTTGCCCGGTTTT<br>TTGATGCGTTACCTCGGTGCGGCGACACCCGGCAGACTGACGCTGGCGATAGAGTTGCCCTGTTTTGCGGCGCTCGTCGCGCTGCTGGTTCTGAGCAC<br>CGCCGGCGTCGGATTTTTTGCGCCCTATCACCTGGAGAGCACCGGCCAATTTACCGCCAAGGCGATCGCCGCGTCATTGTCCGTCGCGGGCCC
pSC3027	Synthetic insert of DNA sequence encoding a C-terminal HA tag (shown in **bold**) on TvpAB (SMDB11_2247) to be introduced into the native genomic locus by allelic exchange (containing *XbaI*/*ApaI* sites (underlined) for sub-cloning into pKNG101). Produced by GeneArt (ThermoFisher):<br>TCTAGAGACATTCAGCCACGCCCGGCTCGACAAAGTCAGCTTCGTCAAATGCCGGTTGCTGGCGGTGAACTTCAGTCAGGCGCGGCTGGAAAGTTGCG<br>CCTGGGTTGACACCGAGACGCAAAGCCTCAGCTTCCACGCCGCACGTCTCACGGCCTGCGCCTTTGCAGCGAAGACACTGTTGCCGCAGGCGGATTTC<br>AGCGACGCGACGCTAAACCAGTGCAATTTACGCCAGATGCCGTTGCAGCGGGCCAATTTTAGCCGGGCGCGTCTCAATAATTGCGATCTGTCGGAAAC<br>CCGATTGAACGAGGCCGATTTCCGCCAGGCCAACGGCAGTGGCAGCCTGTTCATCCGCAGCGATTTGTCTCGCGCCAGCCTGCGCGATGCCAATTTCAT<br>TGCGGCTATCCTGCAAAAATGCGTGCTGTCAGGCGCCGACCTGCAGGGCGCCAATCTGTTCCGGGCGGACCTGTCGCAGTCGCAAGTGGATCAGGCA<br>ACCCGGCTCGAGAGCGCCTATACCGCCAGGGTGAAAACATTGCCTCGCCACAACGGGAAGGAAGTA**TATCCTTATGATGTTCCTGATTATGCA**TGACA<br>ACGGGAAGGAAGTATGACCTCAAAACCAGAGCAGATACGCCAGCGCGTAAAACGCGGCGAGCCGATCGCCGGAGAGGACTTGCACGGCCTGTCATT<br>CGCCGGTCTGGATTTGGCCGGCGGCATGTTCAACGAACTGAACCTGAACGGCGTGAACTTCTCTGACTGCGATCTGCGCGACAGCGTTTTCAGCGATT<br>GCCAGCTCGAACACGCACAATTTACGCGGGCGGATCTGAAGCAAACGGCCTTCAATCAGTGCGCCATGTCGGCCAGCCGCTTCAGCGAGAGCCATATC<br>GAACTGACGATGTTCAACGATTGCCGGCTTGAACAGAGCGACTTCAGCCGGCTGTCGCTCAACCAAAGTCACTGGATGTCGTGCCAATTGGCCGGCGC<br>GAATTTTTCCGCCACTCAGCACGATCGCACTACCTTTTACGAAAGCCCGCTCGACGGCGCCATGTTGAATCAGGCGCGCTTATGCCTTGTCACGTTCTTT<br>CGATTAAATCTCTGTAAAACGCAGTTTGAAGGCGTGAACTTCGACCGCGTCACGTTTTGGGCCC
pSC4407	Synthetic insert of DNA sequence encoding an N-terminal HA tag (shown in **bold**) on TvpC with substitutions F138E L152E I159E (in red) to be introduced into the native genomic locus by allelic exchange (replacing a ΔtvpC allele) (contains *BglII-SpeI* sites (underlined) for sub-cloning into pKNG101). Produced by GenScript :<br>AGATCTCAGTTTGAAGGCGTGAACTTCGACCGCGTCACGTTTTTCGAATGTGACCACCGCGGCAAAAGTTACGCCGGGCAGAGCCTGATCGCCTGCCA<br>GTTTACCGATAACCAATTGGACGACGTCGACTTCAGCCAGGCGACGCTGCGCCAGAGCAATTTCAAGGGCGCTTCGCTGCGGCGCGCCAACCTGACCA<br>GCGTACAGGCGCAGCAGTCGCTGTGGCTGGAGGCCAATCTGACTCAGGCGCAGTGTCGCGGCGGGCAGTTCGATCAGGCGATCTTCAGCGAAGCGAC<br>GTTGGATGCCGCCAACTTCAGCCAGGCGCGCCTGTATCAATGCGTATTTCAGCACAGCCGATCGGCGCGCTGTGATTACAGCGACAGCGACCTGACCT<br>ACGCCGACTTCTGTTATGCCGACATTGGCGCCGCCGATTTCCGGCGCGCGCGCTTTATGCGCACCCGTATGCATCGCGCCCACCAGCAGCAAACGCGCT

	GGAGCGATCGCAGCGGGATCCTCGAGAGCGACGAAGAGCTGTACGCCGCCGAAACCTGGAGCGCACAGCGCCAGAGCCGAATTTGAATGCATTGAC<br>CACTTCACGAGGGGATCACCATG**TATCCTTATGATGTTCCTGATTATGCA**AGCGTTGCCAACACAACTCGCGCCGTCAACATCGCACCGCCCCAACAGG<br>CCGCTGGCCAGGTCGTCAACCTGTTGCCGGACGGCAGTCTGGTGGTGGAATGTGAAGGTCGTGGCTGGCATTGTCGCCGGGCGGCGAGCTGCCTGCT<br>GACGCCGGCCCTCGGCGATAACGTGCTGGTCGCCGGCTGCGGCCACCAGCTGTGGGCGATCGCCGTCCTCGAGCGGGCAGAGCCGCAAAGCACGGC<br>GCGGCTCTGCGTCGCAGGCGATCTGCAGATTGAAACGCCGAACGGCTCGCTGTCGTTGCACAGCGCGCAGGCGCTGAAACTGAGCGGCGACGCCATG<br>ACGCTGCAGGCGAACAGCGGCGACTGCCACGTCGACAAGATGAAATACAGCGGCGAAGAGCTGTCGGCCGAAGTCAGCATCAGCCGTTTGGTCGGTA<br>AACGCTGCGAGTCGGAATGGCATTCGGTCAGCCAGGAAAGCCATTCGCTGTTCCGCAAAGTGCGCCAAACCGAGCACGTGCGCGCCGGGCAGCTGGA<br>TTATCAGGCGGAAGATTACGCCCGCATTCATGCCCGCAACACGCTCATTACCTCAAAAGACATCACCAAGCTGGATTCAGAACAAATCCACGTCGGATA<br>ACCTGCGGAGGCCTTTTATGTTTGCCAACAGCCAAATGATCGGCGTGGACCTGGCGTTTCCCGACGTCTGTCTGACGCCAAGCCCGGCGCCGGTGCCC<br>GTACCCTACCCGGATATCGCCCTCGCGCCGACCGCCATCCCGAACGCGTTCAACATTCTGTTCGTCGGTACCCCGGCGCACAACATGGCGACGGTAACG<br>CCCCTGACCAACGGCGACAACCCCGGCGTAGCCACCGGTGTCGCTTCCGGGACGGTGATGGGGCCATCCCGCCACCTGACCGGCGCCTTTACCGTGTT<br>GCTCAAAGGCACTCCGGCCACCCGGCTGACCAGCCTCAGCCTGCAGAATTCCACCAACGCGCTCGGCATGCGCATCGTGCCGAGCCAATTAAAAGTGT<br>TGCTGCTTGCGCCCTGATGACGGCGCCTTATTACACAGCCATTCAATCAAGAAAAAGAGGACATTGTGAAAACACGGATTACCCTGACGCTGCTGACCG<br>CCCTGACGCTTGCCGGTTGTTCCACCCCGCCGCCACCGCCGCCGGCGCTCAATAACGACGCCCTCATCAGCAGTGAAGTCAACGGCGTCACCCTGCAGC<br>ACCGCGCCGCACTAGT
pSC4408	Synthetic insert of DNA sequence encoding an N-terminal HA tag (shown in **bold**) on TvpC with substitutions D204R D199R E206R (in red) to be introduced into the native genomic locus by allelic exchange (replacing a ΔtvpC allele) (contains *BglII-SpeI* sites (underlined) for sub-cloning into pKNG101). Produced by GenScript :<br>AGATCTCAGTTTGAAGGCGTGAACTTCGACCGCGTCACGTTTTTCGAATGTGACCACCGCGGCAAAAGTTACGCCGGGCAGAGCCTGATCGCCTGCCA<br>GTTTACCGATAACCAATTGGACGACGTCGACTTCAGCCAGGCGACGCTGCGCCAGAGCAATTTCAAGGGCGCTTCGCTGCGGCGCGCCAACCTGACCA<br>GCGTACAGGCGCAGCAGTCGCTGTGGCTGGAGGCCAATCTGACTCAGGCGCAGTGTCGCGGCGGGCAGTTCGATCAGGCGATCTTCAGCGAAGCGAC<br>GTTGGATGCCGCCAACTTCAGCCAGGCGCGCCTGTATCAATGCGTATTTCAGCACAGCCGATCGGCGCGCTGTGATTACAGCGACAGCGACCTGACCT<br>ACGCCGACTTCTGTTATGCCGACATTGGCGCCGCCGATTTCCGGCGCGCGCGCTTTATGCGCACCCGTATGCATCGCGCCCACCAGCAGCAAACGCGCT<br>GGAGCGATCGCAGCGGGATCCTCGAGAGCGACGAAGAGCTGTACGCCGCCGAAACCTGGAGCGCACAGCGCCAGAGCCGAATTTGAATGCATTGAC<br>CACTTCACGAGGGGATCACCATG**TATCCTTATGATGTTCCTGATTATGCA**AGCGTTGCCAACACAACTCGCGCCGTCAACATCGCACCGCCCCAACAGG<br>CCGCTGGCCAGGTCGTCAACCTGTTGCCGGACGGCAGTCTGGTGGTGGAATGTGAAGGTCGTGGCTGGCATTGTCGCCGGGCGGCGAGCTGCCTGCT<br>GACGCCGGCCCTCGGCGATAACGTGCTGGTCGCCGGCTGCGGCCACCAGCTGTGGGCGATCGCCGTCCTCGAGCGGGCAGAGCCGCAAAGCACGGC<br>GCGGCTCTGCGTCGCAGGCGATCTGCAGATTGAAACGCCGAACGGCTCGCTGTCGTTGCACAGCGCGCAGGCGCTGAAACTGAGCGGCGACGCCATG<br>ACGCTGCAGGCGAACAGCGGCGACTGCCACGTCGACAAGATGAAATACAGCGGCGAAGAGCTGTCGGCCTTCGTCAGCATCAGCCGTTTGGTCGGTA<br>AACGCTGCGAGTCGCTGTGGCATTCGGTCAGCCAGATCAGCCATTCGCTGTTCCGCAAAGTGCGCCAAACCGAGCACGTGCGCGCCGGGCAGCTGGA<br>TTATCAGGCGGAAGATTACGCCCGCATTCATGCCCGCAACACGCTCATTACCTCAAAACGTATCACCAAGCTGCGTTCACGTCAAATCCACGTCGGATA<br>ACCTGCGGAGGCCTTTTATGTTTGCCAACAGCCAAATGATCGGCGTGGACCTGGCGTTTCCCGACGTCTGTCTGACGCCAAGCCCGGCGCCGGTGCCC<br>GTACCCTACCCGGATATCGCCCTCGCGCCGACCGCCATCCCGAACGCGTTCAACATTCTGTTCGTCGGTACCCCGGCGCACAACATGGCGACGGTAACG<br>CCCCTGACCAACGGCGACAACCCCGGCGTAGCCACCGGTGTCGCTTCCGGGACGGTGATGGGGCCATCCCGCCACCTGACCGGCGCCTTTACCGTGTT<br>GCTCAAAGGCACTCCGGCCACCCGGCTGACCAGCCTCAGCCTGCAGAATTCCACCAACGCGCTCGGCATGCGCATCGTGCCGAGCCAATTAAAAGTGT<br>TGCTGCTTGCGCCCTGATGACGGCGCCTTATTACACAGCCATTCAATCAAGAAAAAGAGGACATTGTGAAAACACGGATTACCCTGACGCTGCTGACCG<br>CCCTGACGCTTGCCGGTTGTTCCACCCCGCCGCCACCGCCGCCGGCGCTCAATAACGACGCCCTCATCAGCAGTGAAGTCAACGGCGTCACCCTGCAGC<br>ACCGCGCCGCACTAGT
pSC4409	Synthetic insert of DNA sequence encoding an N-terminal HA tag (shown in **bold**) on TvpC with substitution V210Q (in red) to be introduced into the native genomic locus by allelic exchange (replacing a ΔtvpC allele) (contains *BglII-SpeI* sites (underlined) for sub-cloning into pKNG101). Produced by GenScript :<br>AGATCTCAGTTTGAAGGCGTGAACTTCGACCGCGTCACGTTTTTCGAATGTGACCACCGCGGCAAAAGTTACGCCGGGCAGAGCCTGATCGCCTGCCA<br>GTTTACCGATAACCAATTGGACGACGTCGACTTCAGCCAGGCGACGCTGCGCCAGAGCAATTTCAAGGGCGCTTCGCTGCGGCGCGCCAACCTGACCA<br>GCGTACAGGCGCAGCAGTCGCTGTGGCTGGAGGCCAATCTGACTCAGGCGCAGTGTCGCGGCGGGCAGTTCGATCAGGCGATCTTCAGCGAAGCGAC<br>GTTGGATGCCGCCAACTTCAGCCAGGCGCGCCTGTATCAATGCGTATTTCAGCACAGCCGATCGGCGCGCTGTGATTACAGCGACAGCGACCTGACCT<br>ACGCCGACTTCTGTTATGCCGACATTGGCGCCGCCGATTTCCGGCGCGCGCGCTTTATGCGCACCCGTATGCATCGCGCCCACCAGCAGCAAACGCGCT<br>GGAGCGATCGCAGCGGGATCCTCGAGAGCGACGAAGAGCTGTACGCCGCCGAAACCTGGAGCGCACAGCGCCAGAGCCGAATTTGAATGCATTGAC<br>CACTTCACGAGGGGATCACCATG**TATCCTTATGATGTTCCTGATTATGCA**AGCGTTGCCAACACAACTCGCGCCGTCAACATCGCACCGCCCCAACAGG<br>CCGCTGGCCAGGTCGTCAACCTGTTGCCGGACGGCAGTCTGGTGGTGGAATGTGAAGGTCGTGGCTGGCATTGTCGCCGGGCGGCGAGCTGCCTGCT<br>GACGCCGGCCCTCGGCGATAACGTGCTGGTCGCCGGCTGCGGCCACCAGCTGTGGGCGATCGCCGTCCTCGAGCGGGCAGAGCCGCAAAGCACGGC<br>GCGGCTCTGCGTCGCAGGCGATCTGCAGATTGAAACGCCGAACGGCTCGCTGTCGTTGCACAGCGCGCAGGCGCTGAAACTGAGCGGCGACGCCATG<br>ACGCTGCAGGCGAACAGCGGCGACTGCCACGTCGACAAGATGAAATACAGCGGCGAAGAGCTGTCGGCCTTCGTCAGCATCAGCCGTTTGGTCGGTA<br>AACGCTGCGAGTCGCTGTGGCATTCGGTCAGCCAGATCAGCCATTCGCTGTTCCGCAAAGTGCGCCAAACCGAGCACGTGCGCGCCGGGCAGCTGGA<br>TTATCAGGCGGAAGATTACGCCCGCATTCATGCCCGCAACACGCTCATTACCTCAAAAGACATCACCAAGCTGGATTCAGAACAAATCCACCAAGGATA<br>ACCTGCGGAGGCCTTTTATGTTTGCCAACAGCCAAATGATCGGCGTGGACCTGGCGTTTCCCGACGTCTGTCTGACGCCAAGCCCGGCGCCGGTGCCC<br>GTACCCTACCCGGATATCGCCCTCGCGCCGACCGCCATCCCGAACGCGTTCAACATTCTGTTCGTCGGTACCCCGGCGCACAACATGGCGACGGTAACG<br>CCCCTGACCAACGGCGACAACCCCGGCGTAGCCACCGGTGTCGCTTCCGGGACGGTGATGGGGCCATCCCGCCACCTGACCGGCGCCTTTACCGTGTT<br>GCTCAAAGGCACTCCGGCCACCCGGCTGACCAGCCTCAGCCTGCAGAATTCCACCAACGCGCTCGGCATGCGCATCGTGCCGAGCCAATTAAAAGTGT<br>TGCTGCTTGCGCCCTGATGACGGCGCCTTATTACACAGCCATTCAATCAAGAAAAAGAGGACATTGTGAAAACACGGATTACCCTGACGCTGCTGACCG<br>CCCTGACGCTTGCCGGTTGTTCCACCCCGCCGCCACCGCCGCCGGCGCTCAATAACGACGCCCTCATCAGCAGTGAAGTCAACGGCGTCACCCTGCAGC<br>ACCGCGCCGCACTAGT

[a] Incorporated restriction sites for cloning into the respective vector are underlined.
[b] Sequences encoding protein epitope/affinity tags are in bold.

Appendix Table S3. Oligonucleotide primers and synthetic gene fragments used for plasmid construction.

Appendix Table S4

Protein/Complex	pLDDT	pTM	ipTM
***Serratia marcescens* Db10**			
VgrG1	88	n.a.	n.a.
VgrG2	91.3	n.a.	n.a.
TvpC	93.1	n.a.	n.a.
TvpAB	92.8	n.a.	n.a.
Db10 VgrG1-PAAR	86.5	0.792	0.776
Db10 pre-complex (TvpAB-TvpB-TvpC-PAAR)	92.3	0.898	0.903
***Agrobacterium tumefaciens* C58**			
VgrG2 (Atu3642)	92.1	n.a.	n.a.
Tap2	93.1	n.a.	n.a.
VgrG2 full complex (VgrG2-Tap2-PAAR Tde2)	87.9	0.796	0.778
***Pseudomonas aeruginosa* PAO1**			
VgrG1b (PA0095)	93.4	n.a.	n.a.
PA0096	77	n.a.	n.a.
PA0097	89.8	n.a.	n.a.
PAO1 pre-complex (PA0096-PA0097-PA0098-PAAR Tse7)	84.3	0.789	0.753

Appendix Table S4: Summary of the confidence scores of the Alphafold predictions presented in Figures 4 and 5. Overall confidence scores of each AlphaFold prediction (pLDDT) and specific confidence scores for complexes (pTM and ipTM) are indicated. The PAAR domain of Tde2 corresponds to amino acids 1-172, and the PAAR domain of Tse7 corresponds to amino acids 1-140.

Appendix Table S5

Protein	β prism length (Å)	pLDDT
***Serratia marcescens* Db10**		
VgrG1	186	88
VgrG2	81	91.3
***Agrobacterium tumefaciens* C58**		
VgrG2 (Atu3642)	96	92.1
VgrG1 (Atu4348)	118	84.9
***Pseudomonas aeruginosa* PAO1**		
VgrG1b (PA0095)	154	93.4
VgrG1a (PA0091)	76	89.6
VgrG1c (PA2685)	117	86.1
VgrG2a (PA1511)	110	83.7
VgrG2b (PA0262)	116	80.1
VgrG3 (PA2373)	117	88.2
VgrG4a (PA3294)	80	82.1
VgrG4b (PA3486)	80	76.8
VgrG5 (PA5090)	80	78.9
VgrG6 (PA5266)	80	82.5
***Burkholderia pseudomallei* K96243**		
VgrG3 (BPSS0181)	95	80.2
VgrG4a (BPSS0523)	X	71.6
VgrG4b (BPSS0524)	X	71.5
VgrG5 (BPSS1503)	165	65.6
VgrG (BPSS2056)	148	86.1
VgrG (BPSS0958)	89	86.9
VgrG2 (BPSS0105)	95	73
VgrG6 (BPSS2093)	152	87.6
***Burkholderia thailandensis* E264**		
VgrG4a (BTH_II1894)	X	72.6
VgrG4b (BTH_II1893)	X	70.3
VgrG5 (BTH_II0863)	163	66.3
VgrG (BTH_II1436)	89	86.9
VgrG (BTH_I2705)	105	75.6
VgrG2 (BTH_II0129)	95	75.6
VgrG6 (BTH_II0265)	151	87.8
VgrG (BTH_II1531)	105	77.1
VgrG (BTH_I2693)	109	71.9
VgrG (BTH_I2697)	105	76.4
***Pantoea ananatis* PA13**		
VgrG (PAGR_g1676)	110	80.6
VgrG (PAGR_g1684)	75	83.6
***Cronobacter sakazakii* ES15**		
VgrG (ES15_3822)	159	82.1
VgrG (ES15_3808)	76	87.1
VgrG (ES15_3826)	99	86
VgrG (ES15_2015)	109	86.5
VgrG (ES15_2809)	100	76.4
***Proteus mirabilis* HI4320**		

Appendix Table S5

VgrG1 (PMI0751)	85	80.3
VgrG2 (PMI0208)	80	83.5
VgrG3 (PMI1118)	76	81.2
VgrG4 (PMI1331)	80	83.8
VgrG5 (PMI2991)	99	82.5
***Vibrio parahaemolyticus* RIMD 2210633**		
VgrG1 (VP1394)	125	88.9
VgrG (VPA1026)	n.d.	n.d.

Appendix Table S5: Summary Table of VgrG β-prism length measurements. Structural models of all the VgrG proteins in the set of eight representative bacteria (*S. marcescens* Db10, *A. tumefaciens* C58, *P. ananatis* PA13, *C. sakazakii* ES15, *P. mirabilis* HI4320, *P. aeruginosa* PAO1, *B. pseudomallei* K96243 and *B. thailandensis* E264) and in *V. parahamolyticus* RIMD 2210633 were generated using AlphaFold3. Each model was visualized with PyMol to measure the length of the β-prism when possible. These measurements were used to generate Figure 8C. Confidence scores (pLDDT) for each AlphaFold prediction are also indicated. n.d : not determined.

References for Appendix

References

Alcoforado Diniz J & Coulthurst SJ (2015) Intraspecies Competition in Serratia marcescens Is Mediated by Type VI-Secreted Rhs Effectors and a Conserved Effector-Associated Accessory Protein. *J Bacteriol* 197: 2350–2360

Blattner FR, Plunkett G, Bloch CA, Perna NT, Burland V, Riley M, Collado-Vides J, Glasner JD, Rode CK, Mayhew GF, *et al* (1997) The complete genome sequence of Escherichia coli K-12. *Science* 277: 1453–1462

Blum M, Andreeva A, Florentino LC, Chuguransky SR, Grego T, Hobbs E, Pinto BL, Orr A, Paysan-Lafosse T, Ponamareva I, *et al* (2025) InterPro: the protein sequence classification resource in 2025. *Nucleic Acids Research* 53: D444–D456

Cianfanelli FR, Alcoforado Diniz J, Guo M, De Cesare V, Trost M & Coulthurst SJ (2016) VgrG and PAAR Proteins Define Distinct Versions of a Functional Type VI Secretion System. *PLoS Pathog* 12: e1005735

Dereeper A, Guignon V, Blanc G, Audic S, Buffet S, Chevenet F, Dufayard J-F, Guindon S, Lefort V, Lescot M, *et al* (2008) Phylogeny.fr: robust phylogenetic analysis for the non-specialist. *Nucleic Acids Res* 36: W465-469

Edgar RC (2004) MUSCLE: multiple sequence alignment with high accuracy and high throughput. *Nucleic Acids Research* 32: 1792–1797

English G, Trunk K, Rao VA, Srikannathasan V, Hunter WN & Coulthurst SJ (2012) New secreted toxins and immunity proteins encoded within the T ype VI secretion system gene cluster of *S erratia marcescens* . *Molecular Microbiology* 86: 921–936

Flyg C, Kenne K & Boman HG (1980) Insect Pathogenic Properties of Serratia marcescens: Phage-resistant Mutants with a Decreased Resistance to Cecropia Immunity and a Decreased Virulence to Drosophila. *Microbiology* 120: 173–181

Guzman LM, Belin D, Carson MJ & Beckwith J (1995) Tight regulation, modulation, and high-level expression by vectors containing the arabinose PBAD promoter. *J Bacteriol* 177: 4121–4130

Herrero M, De Lorenzo V & Timmis KN (1990) Transposon vectors containing non-antibiotic resistance selection markers for cloning and stable chromosomal insertion of foreign genes in gram-negative bacteria. *J Bacteriol* 172: 6557–6567

Iguchi A, Nagaya Y, Pradel E, Ooka T, Ogura Y, Katsura K, Kurokawa K, Oshima K, Hattori M, Parkhill J, *et al* (2014) Genome Evolution and Plasticity of Serratia marcescens, an Important Multidrug-Resistant Nosocomial Pathogen. *Genome Biology and Evolution* 6: 2096–2110

Jack RL, Buchanan G, Dubini A, Hatzixanthis K, Palmer T & Sargent F (2004) Coordinating assembly and export of complex bacterial proteins. *EMBO J* 23: 3962–3972

Kaniga K, Delor I & Cornelis GR (1991) A wide-host-range suicide vector for improving reverse genetics in Gram-negative bacteria: inactivation of the blaA gene of Yersinia enterocolitica. *Gene* 109: 137–141

References for Appendix

Karimova G, Pidoux J, Ullmann A & Ladant D (1998) A bacterial two-hybrid system based on a reconstituted signal transduction pathway. *Proc Natl Acad Sci U S A* 95: 5752–5756

Karimova G, Ullmann A & Ladant D (2000) A bacterial two-hybrid system that exploits a cAMP signaling cascade in Escherichia coli. *Methods Enzymol* 328: 59–73

Meng EC, Goddard TD, Pettersen EF, Couch GS, Pearson ZJ, Morris JH & Ferrin TE (2023) UCSF ChimeraX : Tools for structure building and analysis. *Protein Science* 32: e4792

Mistry J, Chuguransky S, Williams L, Qureshi M, Salazar GA, Sonnhammer ELL, Tosatto SCE, Paladin L, Raj S, Richardson LJ, *et al* (2021) Pfam: The protein families database in 2021. *Nucleic Acids Research* 49: D412–D419

Murdoch SL, Trunk K, English G, Fritsch MJ, Pourkarimi E & Coulthurst SJ (2011) The opportunistic pathogen Serratia marcescens utilizes type VI secretion to target bacterial competitors. *J Bacteriol* 193: 6057–6069

Reglinski M, Hurst QW, Williams DJ, Gierlinski M, Şahin AT, Mathers K, Ostrowski A, Bergkessel M, Zachariae U, Pitt SJ, *et al* (2025) A widely-occurring family of pore-forming effectors broadens the impact of the Serratia Type VI secretion system. *EMBO J* 44: 6892–6918

Sachar K, Kanarek K, Colautti J, Kim Y, Bosis E, Prehna G, Salomon D & Whitney JC (2025) A conserved chaperone protein is required for the formation of a noncanonical type VI secretion system spike tip complex. *J Biol Chem* 301: 108242

Thompson J (1997) The CLUSTAL_X windows interface: flexible strategies for multiple sequence alignment aided by quality analysis tools. *Nucleic Acids Research* 25: 4876–4882
