# Supplementary material for: Tvp complexes support formation of the VgrG-PAAR spike during Type VI secretion system assembly
Source: EMBO J. 2026 May 29;45(14):5101–29. doi: 10.1038/s44318-026-00820-1 (PMC13373197; doi:10.1038/s44318-026-00820-1)
Supplement: Supplementary file 1 [file 44318_2026_820_MOESM12_ESM.pdf]

Expanded View Figures

Figure EV1. Vai1 is a new immunity protein conferring protection against the effector delivered by the VgrG1 pathway of *Serratia marcescens* Db10.

(**A**) Recovery of *S. marcescens* Db10 Δ*2245–2246* Δ*tssE* target cells following co-culture with wild type (WT) and mutant strains of *S. marcescens* Db10. (**B**) Recovery of Db10 Δ*2245* Δ*tssE* target cells following co-culture with WT and mutant strains of Db10. (**C**) Recovery of Db10 Δ*2246* Δ*tssE* target cells following co-culture with WT and mutant strains of Db10. (**D**) Recovery of the Db10 Δ*2245–2246* Δ*tssE* target complemented with *2245*-FLAG expressed from plasmid pSUPROM, following co-culture with WT and mutant strains of Db10. In (**A–D**), co-cultures were performed with an initial ratio of 1:1. (**E**) Left, Vai1 transmembrane helices prediction using TMHMM 2.0. The potential transmembrane helix in Vai1 (amino acids 7–29) is highlighted in purple. Right, Ribbon representation of the Vai1 structure predicted by AlphaFold2. The protein is coloured based on the reported confidence of the AlphaFold modelling, from orange (pLDDT <50, very low confidence) to dark blue (pLDDT >90, very high confidence). Vai1 insertion in the inner membrane is depicted based on the TMHMM prediction. (**A–D**) Data sets are displayed as mean ± SEM ($n = 4$ biological replicates in (**A–C**) and $n = 3$ biological replicates in (**D**)) with individual data points overlaid. ****$P < 0.0001$, ns not significant; one-way ANOVA with Tukey's test; for clarity, only selected comparisons are displayed. P values from left to right: (**A**) $P < 0.0001$, $P = 0.9977$, $P < 0.0001$, $P < 0.0001$, $P < 0.0001$, $P = 0.0376$, $P = 0.9592$, $P = 0.9864$ and $P < 0.0001$. (**B**) $P = 0.1750$, $P = 0.9850$, $P < 0.0001$, $P = 0.2922$, $P < 0.0001$ and $P < 0.0001$. (**C**) $P = 0.2949$, $P = 0.9139$, $P = 0.8451$, $P = 0.1090$, $P = 0.3699$ and $P = 0.9982$. (**D**) $P = 0.9893$, $P = 0.6671$, $P = 0.8060$, $P = 0.8286$, $P > 0.9999$ and $P = 0.9934$. Source data are available online for this figure.

© The Author(s)

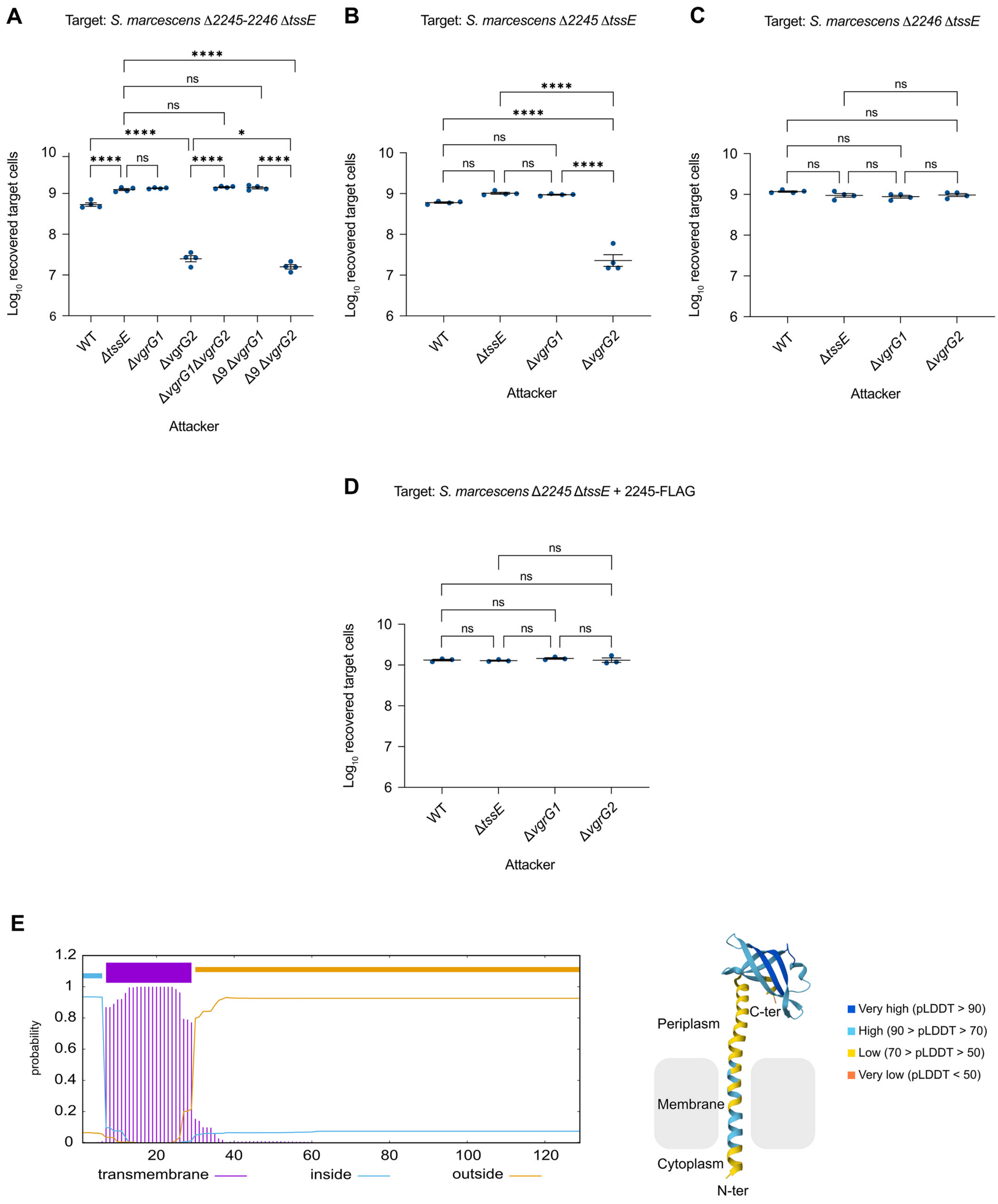
A
Target: S. marcescens Δ2245-2246 ΔtssE
B
Target: S. marcescens Δ2245 ΔtssE
C
Target: S. marcescens Δ2246 ΔtssE
Log10 recovered target cells
****
ns
*
WT
ΔtssE
ΔvgrG1
ΔvgrG2
ΔvgrG1ΔvgrG2
Δ9 ΔvgrG1
Δ9 ΔvgrG2
Attacker
D
Target: S. marcescens Δ2245 ΔtssE + 2245-FLAG
E
probability
transmembrane
inside
outside
Periplasm
Membrane
Cytoplasm
C-ter
N-ter
Very high (pLDDT > 90)
High (90 > pLDDT > 70)
Low (70 > pLDDT > 50)
Very low (pLDDT < 50)

 © The Author(s)

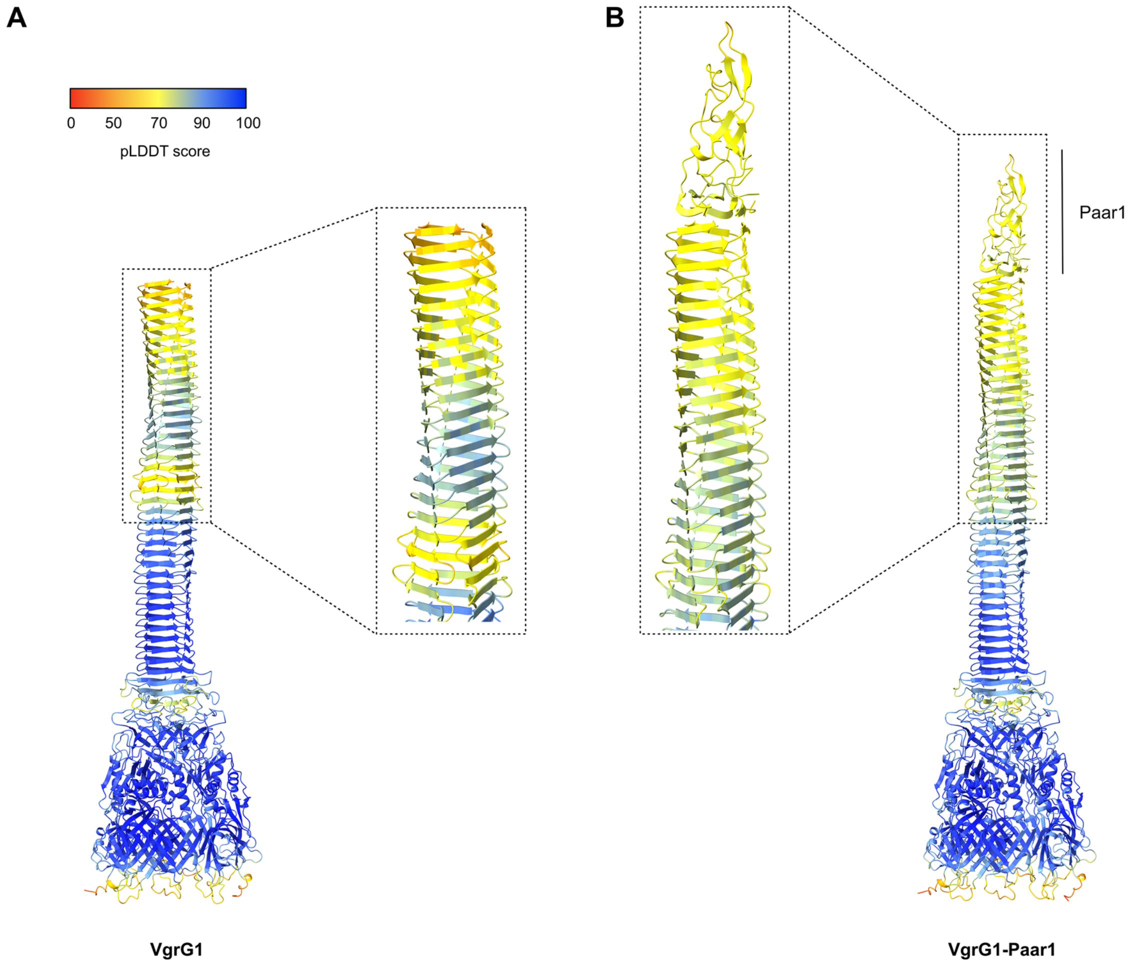

Figure EV2. AlphaFold predictions of VgrG1 alone or in complex with Paar1.

Ribbon representation of predicted structures of VgrG1 from *S. marcescens* Db10 (**A**) alone (as in Fig. 4) and (**B**) in complex with Paar1. A zoom on the VgrG1 tip is provided to show how it could be stabilised by Paar1 protein. Predicted structures were generated by AlphaFold2 and coloured according to the pLDDT score, from red (pLDDT <50, lowest confidence) to blue (pLDDT >90, highest confidence).

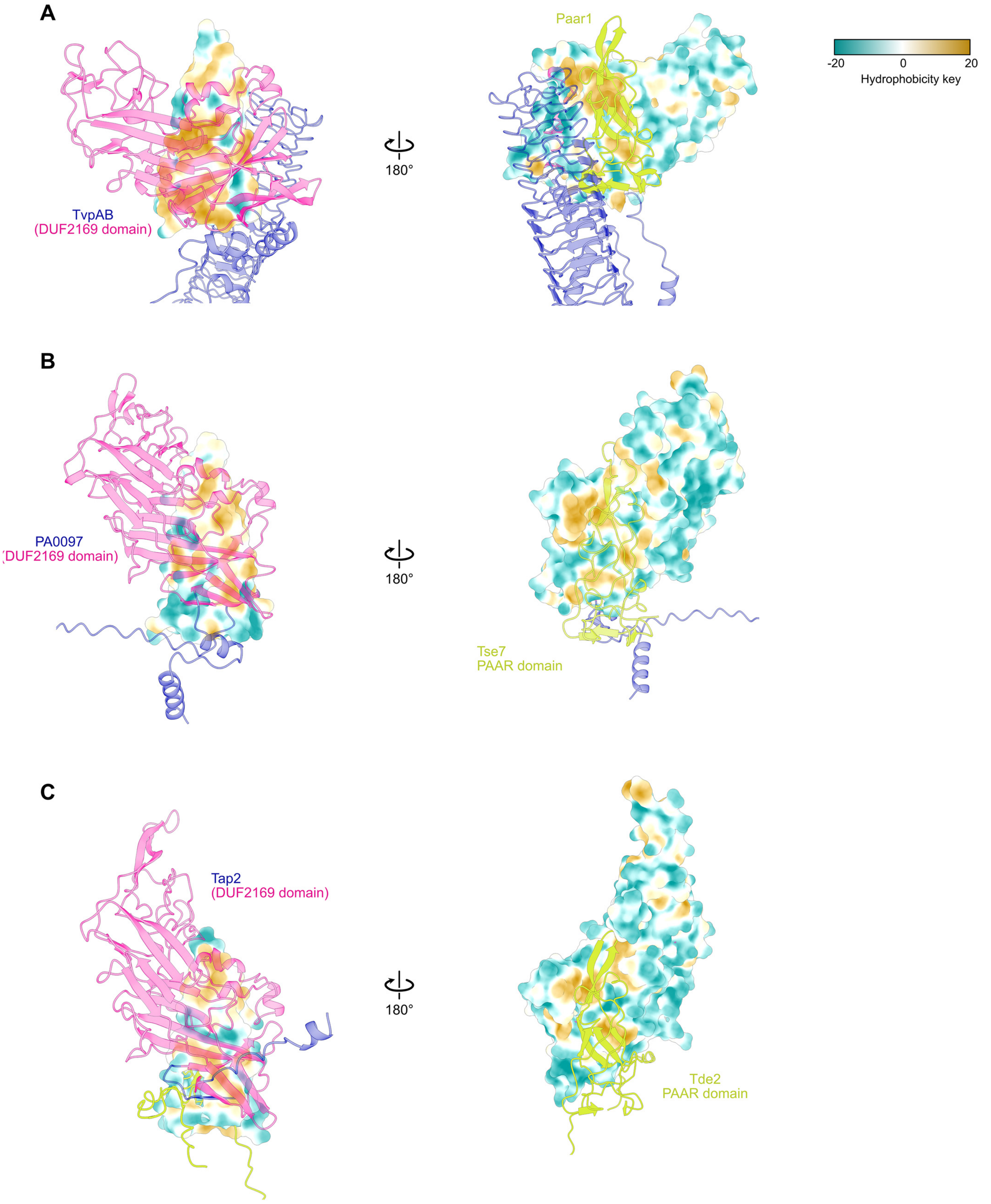
A
Paar1
-20
0
20
Hydrophobicity key
180°
TvpAB
(DUF2169 domain)
B
PA0097
(DUF2169 domain)
180°
Tse7
PAAR domain
C
Tap2
(DUF2169 domain)
180°
Tde2
PAAR domain

© The Author(s)

◀ **Figure EV3. The interaction between the DUF2169 domain of TvpA/TvpAB proteins and the PAAR domain involves hydrophobic patches on both proteins.**

(**A**) Interaction between TvpAB and Paar1 from *S. marcescens* Db10. (**B**) Interaction between PA0097 and the PAAR domain of Tse7 from *P. aeruginosa* PA01. (**C**) Interaction between Tap2 and the PAAR domain of Tde2 from *A. tumefaciens* C58. (**A–C**) Left: The hydrophobic surface of PAAR domains is highlighted and TvpA/TvpAB proteins are represented as a ribbon with their DUF2169 domain coloured in deep pink. Right: The hydrophobic surface of the DUF2169 domains of TvpA/TvpAB is highlighted, whereas the PAAR domains are represented as a ribbon and coloured in light green. Hydrophobic surfaces are coloured from dark cyan (most hydrophilic) to white to dark golden (most lipophilic) as in the key.

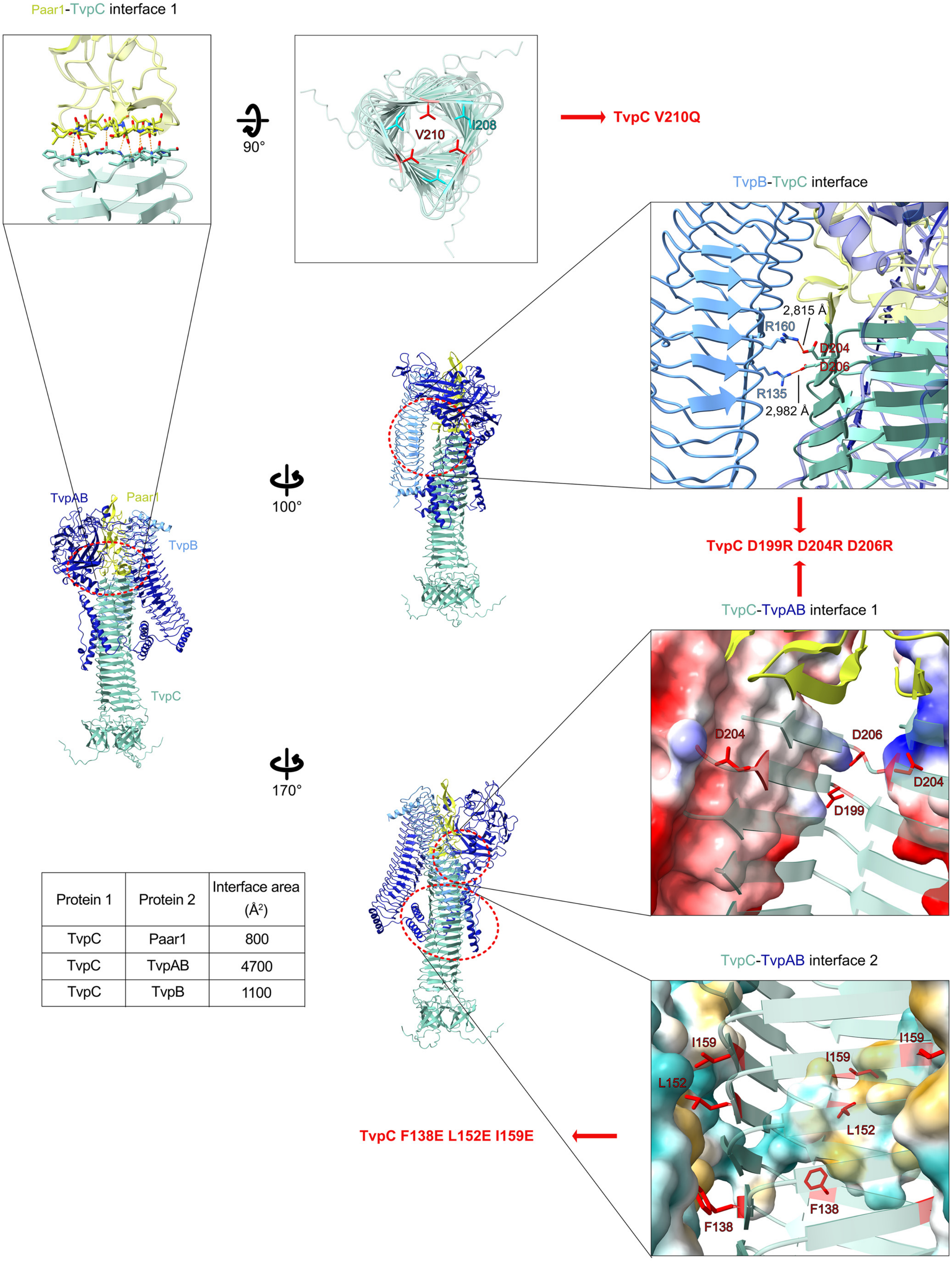

Protein 1	Protein 2	Interface area (Å²)
TvpC	Paar1	800
TvpC	TvpAB	4700
TvpC	TvpB	1100

 © The Author(s)

Figure EV4. Structural view of TvpC amino acids selected for point mutations.

Ribbon representation of *S. marcescens* pre-complex in different orientations with enlarged views of TvpC interfaces with TvpAB, TvpB and Paar1 within the pre-complex. Important residues at the interfaces are represented as sticks, and amino acids selected for substitution are coloured red. TvpAB electrostatic surfaces and hydrophobic surfaces are depicted for TvpC-TvpAB interfaces 1 and 2, respectively. A table summarising the interface areas between TvpC and its partner is provided.

© The Author(s)
