# Supplementary material for: Tvp complexes support formation of the VgrG-PAAR spike during Type VI secretion system assembly
Source: EMBO J. 2026 May 29;45(14):5101–29. doi: 10.1038/s44318-026-00820-1 (PMC13373197; doi:10.1038/s44318-026-00820-1)
Supplement: Supplementary file 3 [file 44318_2026_820_MOESM2_ESM.pdf]

THE EMBO JOURNAL

Tvp complexes support formation of the VgrG-PAAR spike during Type VI secretion system assembly

Laura Monlezun, Christopher Earl, and Sarah Coulthurst

Corresponding author(s): Sarah Coulthurst (s.j.coulthurst@dundee.ac.uk)

Review Timeline:

Submission Date:	25th Aug 25
Editorial Decision:	24th Sep 25
Revision Received:	6th Apr 26
Editorial Decision:	27th Apr 26
Revision Received:	5th May 26
Accepted:	11th May 26

Editor: Ieva Gailite

Transaction Report:

(Note: With the exception of the correction of typographical or spelling errors that could be a source of ambiguity, letters and reports are not edited. Depending on transfer agreements, referee reports obtained elsewhere may or may not be included in this compilation. Referee reports are anonymous unless the Referee chooses to sign their reports.)

1st Editorial Decision 24th Sep 2025

Dear Sarah,

Thank you for submitting your manuscript for consideration by the EMBO Journal. We have now received comments from three reviewers, which are included below for your information.

As you will see, all reviewers find the study of interest, while also indicating several overlapping concerns that would need to be addressed before they can support publication here. These aspects can be summarised as:
1) clarification of the proposed toxin function of VgrG1,
2) validation of the AlphaFold models with mutational analysis in case of difficulties with direct structural validation,
3) validation of the two-step Tvp assembly model, e.g., via in vitro reconstitution experiments.
Based on the interest expressed in the reviewers' reports, I invite you to submit a revised manuscript in response to the comments by all reviewers.

I would be happy to discuss the revision in more detail via email or phone/videoconferencing.

We generally allow up to six months as standard revision time. Should you foresee a problem in meeting this deadline, please let us know in advance to discuss an extension.

As a matter of policy, competing manuscripts published during this period will not negatively impact on our assessment of the conceptual advance presented by your study. However, please contact me as soon as possible upon publication of any related work to discuss the appropriate course of action.

When preparing your letter of response to the referees' comments, please bear in mind that this will form part of the Review Process File and will therefore be available online to the community. For more details on our Transparent Editorial Process, please visit our website: https://www.embopress.org/page/journal/14602075/authorguide#transparentprocess. Please also see the attached instructions for further guidelines on preparation of the revised manuscript.

Please feel free to contact me if you have any further questions regarding the revision. Thank you for the opportunity to consider your work for publication. I look forward to discussing your revision.

With best wishes,

Ieva

Ieva Gailite, PhD
Senior Scientific Editor
The EMBO Journal
Meyerhofstrasse 1
D-69117 Heidelberg
Tel: +4962218891309
i.gailite@embojournal.org

Instructions for preparing your revised manuscript:

Please make sure you upload a letter of response to the referees' comments together with the revised manuscript.

Please also check that the title and abstract of the manuscript are brief, yet explicit, even to non-specialists.

When assembling figures, please refer to our figure preparation guideline in order to ensure proper formatting and readability in print as well as on screen:
https://bit.ly/EMBOPressFigurePreparationGuideline
See also guidelines for figure legends: https://www.embopress.org/page/journal/14602075/authorguide#figureformat

At EMBO Press we ask authors to provide source data for the main manuscript figures. You will receive a separate email with instructions for providing source data with your revised manuscript, including how to upload and organize the files.

IMPORTANT: When you send the revision we will require
- a point-by-point response to the referees' comments, with a detailed description of the changes made (as a word file).
- a word file of the manuscript text.

- individual production quality figure files (one file per figure)
- a complete author checklist, which you can download from our author guidelines (https://www.embopress.org/page/journal/14602075/authorguide).
- Expanded View files (replacing Supplementary Information)
Please see out instructions to authors
https://www.embopress.org/page/journal/14602075/authorguide#expandedview
- a Reagents and Tools Table as part of the Methods section, which can be downloaded from our author guidelines (https://www.embopress.org/page/journal/14602075/authorguide#structuredmethods)

Please remember: Digital image enhancement is acceptable practice, as long as it accurately represents the original data and conforms to community standards. If a figure has been subjected to significant electronic manipulation, this must be noted in the figure legend or in the 'Materials and Methods' section. The editors reserve the right to request original versions of figures and the original images that were used to assemble the figure.

Further information is available in our Guide For Authors: https://www.embopress.org/page/journal/14602075/authorguide

We realize that it is difficult to revise to a specific deadline. In the interest of protecting the conceptual advance provided by the work, we recommend a revision within 3 months (23rd Dec 2025). Please discuss the revision progress ahead of this time with the editor if you require more time to complete the revisions. Use the link below to submit your revision:

https://emboj.msubmit.net/cgi-bin/main.plex

Referee #1:

The type VI secretion system (T6SS) in Gram-negative bacteria mostly operates as an antibacterial weapon. The T6SS assembly starts with the formation of a bell-shaped membrane complex. The so-called baseplate is then docked onto this complex, and baseplate formation is triggered by the assembly of a puncturing device (PAAR-VgrG). From the base of VgrG, assembly of a sheath circling around an Hcp tube is then initiated, until the sheath contracts and expels through the membrane complex the PAAR-VgrG-Hcp molecules. The T6SS-loading of the antibacterial toxins occurs by way of interaction with either of the PAAR-VgrG-Hcp proteins. Very little is known though on how the loading process occurs, the level of specificity and the requirement for additional helper proteins or chaperones.
In the present study the authors present remarkable findings in which a series of accessory proteins, TvpAB, TvpA and TvpC are associated with the PAAR1-VgrG1 tip of the Serratia marcescens T6SS. Interestingly they also show the requirement for a precomplex in which TvpC acts as a VgrG homologue and thus carries the TvpAB/TvpA/PAAR1 complex, which will subsequently be transferred to VgrG1 for completing the loading 9in the T6SS. The authors present ample details on the predicted structure of these complexes using Alphafold, as well as describing homologues in other bacteria which may slightly vary in terms of composition and have been grouped in 3 distinct classes.
Overall, a very novel concept, which in places is based on very logical hypotheses or predictions but would benefit from further experimental validations. Some suggestions are listed here below.

Specific comments
- The authors propose that VgrG1 delivers a toxin in target cells but have not been able to identify it. They have identified the putative cognate immunity (Vai1) and suggest that most likely the toxin is part of the VgrG1 protein itself. Yet they have no direct evidence to support this conclusion except that the immunity interacts with VgrG1. (fig. 1f). Truncation of VgrG1 may come with a loss of toxicity but that could also be due to a fact of failure of T6SS assembly. Instead, the authors suggest that the last 26 residues of VgrG1 are like hydrophobic peptides which acts as antimicrobial peptides inducing membrane depolarization. Could the peptide be synthesized and tested for this function? Could the interaction with Vai1 narrowed down to this region of VgrG1 (e.g. HDX experiment)?
- The authors compared the VgrG1-PAAR-Tvp assembly to previously described T6SS system in Proteus mirabilis (Alteri et al., PLoS Path, 2013) and Pseudomonas aeruginosa (Pissaridou et al., PNAS, 2018). In this case the toxin is connected to a PAAR domain. Have the authors considered that the PAAR1 protein could also be connected to a toxin, for example by performing pull down using PAAR1 as a bait.
- TvpAB and TvpB are proposed to be part of the tip complex but their putative role as toxin not been tested. Is there a specific reason for excluding this possibility.
- VgrG1 and PAAR1 are likely secreted by the T6SS but will TvpAB and TvpB also secreted or are they released from the complex at soon as the firing starts and therefore not found outside of the cell.
- Using Alphafold, he authors present very compelling 3D prediction of the complexes formed on the VgrG1 tip but have no experimental validation of their models. One possibility would be to design a couple of mutations at the interface between interacting proteins and assess whether functionality of the T6SS is then disrupted.
- A recently published work presents the characterization of a TvpAB-DUF2169 homolog in Vibrio parahaemolyticus (Sachar et

al., JBC, 2025). In this study the authors indicate that DUF2169 interacts with PAAR-like protein which possesses a non-canonical PIPY domain instead. Does PAAR1 has a PIPY domain?
- The preassembly on TvpC instead of VgrG1 is a novel and intriguing concept. The idea that the TvpAB-TvpB-PAAR1 complex then moves from TvpC onto VgrG1, or that VgrG1 displaces TvpC, is not obvious. Would the authors have hypotheses on this exchange might happen without disrupting the structure of the preformed complex.

Minor comments
- The authors suggest that TvAB mediates interference between VgrG1 and VgrG2 pathways (lanes 191-192). Could that be indirect. If TvpAB needed for proper VgrG1 tip assembly, then no VgrG1 activity will result in increased VgrG2 activity, as suggested by the authors in previous sections.
- Lane 102 tssK should be all with italics; lane 175 tss with italics
- The authors indicate that the genes downstream vai1 does not encode an effector (Lane 420), likely based on the use genetic mutants and competition experiments (Fig. 1). Has the gene been expressed directly expressed, for example in Escherichia coli, to assess toxicity?

Referee #2:

In this study, the authors characterize a set of Type VI secretion system accessory proteins, termed Tvp complexes, which are required for the assembly of the VgrG1-PAAR1 spike in Serratia marcescens. Although the DUF2169 domain proteins are known to be important for effector delivery and conserved among mang T6SS organisms, this study provided some additional information by proposing that TvpAB, TvpB, and TvpC formed a pre-complex with PAAR1, TvpC was later replaced by VgrG1, forming VgrG1-effector-PAAR1 complex for delivery. Using antibacterial competition assays, immunoprecipitation, mass spectrometry, structural predictions, and phylogenetic analyses, the authors demonstrate that Tvp complexes are evolutionarily conserved.
Although the authors present an interesting model for Tvp protein function, they do not sufficiently support it with experimental evidence. The precise mechanism of VgrG1-associated toxicity and structural validation of the Tvp complexes also remain unclear. Additionally, the introduction should cite more primary references related to key T6SS discoveries, such as the identification of structural components, adaptors, and chaperones.
Major issues:
1. The bacterial competition assays demonstrate that wild-type Serratia marcescens Db10 can efficiently kill the Δ2245-2246 ΔtssE strain, and the authors identify SMDB11_2245 as the cognate immunity protein for the VgrG1 effector. However, the manuscript reports successful construction of a Δ2245 mutant in the wild-type background. Given that the T6SS is active under these conditions, one would expect that the Δ2245 mutant should be self-intoxicated by VgrG1 and therefore non-viable. The authors should clarify how this strain was obtained and maintained. In addition, the competition assays presented do not include data on the survival of the attacker ("killer") strains. Since differences in growth fitness among the various mutants could confound interpretation of competition outcomes, it is important to provide killer survival data and/or growth curves to confirm that the observed phenotypes are not attributable to differences in bacterial growth.
2. The manuscript does not establish whether the observed VgrG1-associated toxicity is due to VgrG1 itself or to an unidentified VgrG1-dependent effector. To clarify this, the authors should test whether VgrG1 expression in strains lacking SMDB11_2245 and with an inactive T6SS, or in a heterologous host such as E. coli, results in toxicity. Complementation with SMDB11_2245 under these conditions could further confirm its role as the cognate immunity protein. If no intrinsic toxicity of VgrG1 is observed, it would be important to re-examine the existing VgrG1 pull-down mass spectrometry data, as well as the previously published secretome datasets from this group, to identify potential unannotated effectors that may account for the observed phenotype.
3. In Figure 3, all co-immunoprecipitation experiments are performed in the native strain background, which does not provide direct evidence for protein-protein interactions. To strengthen the proposed model, in vitro assays such as pull-down experiments with purified proteins, size-exclusion chromatography, or native PAGE should be conducted. Since the authors suggest that TvpAB, TvpB, and TvpC form a pre-complex with PAAR1 that is subsequently replaced by VgrG1, it would be particularly informative to test whether adding VgrG1 to a pre-assembled TvpAB-TvpB-TvpC-PAAR1 complex can displace TvpC in vitro. Furthermore, when analyzing the co-immunoprecipitated proteins by mass spectrometry, it would be useful to clarify whether other conserved T6SS components were enriched or differentially detected.
4. A substantial portion of the manuscript is devoted to structural predictions of the proposed complexes, but no experimental validation is provided. If obtaining the structure of the entire complex or individual proteins is technically challenging, an alternative approach would be to introduce mutations at the predicted protein-protein interfaces and assess their impact on complex formation and T6SS activity. Such mutational analyses would provide important functional validation of the structural models and significantly strengthen the conclusions.
Minor comments:
1. The expression levels of the complementation plasmids should be verified.
2. In Figure 2c, a cytoplasmic protein control should be included to serve as a loading control and to verify equal cell lysis across samples.

Referee #3:

In this article Monlezun, Earl and Coulthurst describe T6SS spike assemblies involving the additional components called Tvp, in addition to structural VgrG and PAAR components that form a canonical spike complex. They provide experimental evidence that Serratia marcescens assembles multiple complexes involving the PAAR, Tvp proteins and VgrG1. The later seems to be mutually exclusive with TvpC protein which is predicted to mimic the OB-Fold-gp5 moiety of VgrG. They demonstrate that previously uncharacterized Tvp components are necessary for functionality of the final VgrG1 spike assembly. Authors also further predict a range of spike architectures involving different sets of Tvp partners across wide range of bacterial species. The manuscript is novel and interesting, it is written with great precision, but it is not easy to follow. In particular, the second part, where structures are modelled from different organisms and multiple versions of assemblies appear. The only thing that is universal but modular across these systems are protein domains, and for the most part I would consider referring to them by domains rather than mixing it up with names.
The experimental part of the work is solid and brings a lot of new information carefully demonstrated in native settings (i.e. in Serratia, in inter-bacterial competition, experimented with multiple bacterial species). However, the second part based almost entirely on AlphaFold modelling is highly speculative and less synthesized. In general, authors suggest existence of two complexes with PAAR enclosed by DUF2169 and PPR domains. One sits on shorter mimicry version of VgrG (TvpC) and other on true VgrG. What is not clear is how this transition occurs, and what is the benefit or necessity of forming the first complex overall. Can authors really claim it is two-step assembly? The only argument is that without the TvpC, the second (functional?) complex is not assembled, but what is truly missing to support these claims is the evidence that the first complex can transition to the second one.
Importantly, we have to consider that extreme rearrangement would have to be made to extract the TvpC and replace it by VgrG. This to me sounds like a very complicated step to imagine, especially in the context of complexes including PPR domains that seems to have extensive interactions with TvpC in the "pre-complex".

Other major concerns:

Figure 1 b to e - it should be indicated if the observed differences of measurements are statistically significant. For example, this is important for the claims regarding the differences between WT and delta tssE attacker conditions. Figure 2 a-b, d-g - statistical significance of observed differences should also be provided.

Figure 1 d - How can authors explain that in the background of target strain complemented with 2246 there is no more visible difference between the WT and delta tssE attacker conditions? It also seems that complementation with 2245 still presents slight difference between WT vs delta tssE or delta VgrG2 Could this be the difference driven by the missing 2246? What do we know about 2246?

Page 7, line 188 - I find this activating effect of TvpAB confusing. It could be relevant to test the double TvpAB/VgrG2 mutant against these other strains as well. It would be expected, that this mutant should behave like in competition with Pseudomonas fluorescence. This would maybe clarify if it has indeed something to do with the VgrG2 pathway?

Regarding the prediction of multimers and complexes by AlphaFold, the important score to provide is iPTM. Are we sure that these models were predicted using the correct stoichiometry? How was this chosen?

Line 315 - I think this is highly speculative to talk about re-arrangements to a level of providing rmsd values, just by comparing multiple AlphaFold multimer models.

Minor remarks:
In line with previous publications and since it is an abbreviation coming from amino acid motif, it might be better to maintain the entire name PAAR in capital letters,

Page 2, lane 7 - "anchoring of the membrane complex" - the term "anchoring" for membrane proteins usually means a class of membrane proteins that are not integral but anchored by lipidic anchors or via other proteins. In this nomenclature the T6SS membrane complex comprises integral membrane proteins, hence this expression can be misleading.

Page 3, lane 69 - it is not very clear what authors mean by "requirement to occupy the cavity around the spike in the baseplate" - this likely refers to requirement for assembly or function of T6SS which is elaborated in discussion? But here it should be better explained and referenced.

Lane 77 - at first mention of delta tssE background, the phenotype and the rationale of this background should be provided.

Lane 143 - for consistency and clarity it would be better if Db10 would be called "WT" or "Db10 WT"

Page 6, Lane 176 - I think the reference 49 is called before many other references. Please review the order of references accordingly.

Page 7, line 178 - what was the difference with "initial co-culture assays" provided in Supplementary Figure 3, as compared to those presented in Figure 2b? without proper explanation this brings in some confusion.

Page 9, line 264 - it might be overstatement to call these VgrG's non-essential. I see what the authors imply, since they do not appear alone, but it might be more cautious to use term "alternative"?

Supplementary Figure 10 could also be annotated on the figure, not only in legend to simplify the interpretation.

Line 308 - what do authors exactly mean by "it was unable to generate a prediction"?

Line 372 - are there sufficient evidence to claim about the evolution towards increased complexity? This implies the directionality of the evolution, although these classes might have a common ancestor and evolve separately?

Response to Reviewers' Comments (EMBOJ-2025-122266)

We would like to thank the Reviewers for taking the time to positively and constructively review our manuscript. The manuscript has been revised in response to their comments and suggestions and has been significantly improved as a result. The Reviewers' comments have been reproduced (in black text) and our responses are included below each point (in blue text).

Referee #1:

The type VI secretion system (T6SS) in Gram-negative bacteria mostly operates as an antibacterial weapon. The T6SS assembly starts with the formation of a bell-shaped membrane complex. The so-called baseplate is then docked onto this complex, and baseplate formation is triggered by the assembly of a puncturing device (PAAR-VgrG). From the base of VgrG, assembly of a sheath circling around an Hcp tube is then initiated, until the sheath contracts and expels through the membrane complex the PAAR-VgrG-Hcp molecules. The T6SS-loading of the antibacterial toxins occurs by way of interaction with either of the PAAR-VgrG-Hcp proteins. Very little is known though on how the loading process occurs, the level of specificity and the requirement for additional helper proteins or chaperones.

In the present study the authors present remarkable findings in which a series of accessory proteins, TvpAB, TvpA and TvpC are associated with the PAAR1-VgrG1 tip of the Serratia marcescens T6SS. Interestingly they also show the requirement for a precomplex in which TvpC acts as a VgrG homologue and thus carries the TvpAB/TvpA/PAAR1 complex, which will subsequently be transferred to VgrG1 for completing the loading 9in the T6SS. The authors present ample details on the predicted structure of these complexes using Alphafold, as well as describing homologues in other bacteria which may slightly vary in terms of composition and have been grouped in 3 distinct classes.

Overall, a very novel concept, which in places is based on very logical hypotheses or predictions but would benefit from further experimental validations. Some suggestions are listed here below.

Specific comments

- The authors propose that VgrG1 delivers a toxin in target cells but have not been able to identify it. They have identified the putative cognate immunity (Vai1) and suggest that most likely the toxin is part of the VgrG1 protein itself. Yet they have no direct evidence to support this conclusion except that the immunity interacts with VgrG1. (fig. 1f). Truncation of VgrG1 may come with a loss of toxicity but that could also be due to a fact of failure of T6SS assembly. Instead, the authors suggest that the last 26 residues of VgrG1 are like hydrophobic peptides which acts as antimicrobial peptides inducing membrane depolarization. Could the peptide be synthesized and tested for this function? Could the interaction with Vai1 narrowed down to this region of VgrG1 (e.g. HDX experiment)?

Following the Reviewer's thoughtful suggestions, we obtained a synthetic peptide corresponding to the last 26 residues of VgrG. We then attempted to demonstrate that this peptide has the ability to induce depolarisation when applied externally to bacterial cells, by

measuring DiBAC$_4$(3) fluorescence in order to mirror our findings upon delivery of full-length VgrG1. Since the T6SS would deliver any peptide within the end of VgrG1 past the outer membrane and into the periplasm, we considered the likely scenario that an isolated peptide would not be able to cross the outer membrane by itself and included the approach of applying the peptide in combination with polymyxin B nonapeptide to permeabilise the outer membrane in our experiments. Unfortunately, we were unable to overcome technical obstacles in order to achieve reproducible and robust data to include in the revised manuscript within the time available. The hydrophobic peptide is hard to handle and sticks to plasticware and, more importantly, our VgrG1-derived and other antimicrobial peptides also bind to DiBAC$_4$(3), causing it to fluoresce non-specifically, particularly at the higher concentrations required to achieve what looked like a potential biological effect on the cells. It proved impossible to find a suitable concentration of peptide and dye at which a specific effect on the cells, although potentially present, could be reproducibly distinguished from background signal of peptide binding dye.

Unfortunately we were not able to perform an HDX experiment since it is an *in vitro* assay requiring purified proteins. We have not been able to successfully purify VgrG1 and note that since Vai1 is a membrane protein, it is likely to be hard to purify and then detergents from the purification might mask an interaction with a VgrG1-derived peptide.

- The authors compared the VgrG1-PAAR-Tvp assembly to previously described T6SS system in Proteus mirabilis (Alteri et al., PLoS Path, 2013) and Pseudomonas aeruginosa (Pissaridou et al., PNAS, 2018). In this case the toxin is connected to a PAAR domain. Have the authors considered that the PAAR1 protein could also be connected to a toxin, for example by performing pull down using PAAR1 as a bait.

Yes, we hoped to do pull-down assays using Paar1 as bait. We have tested a C-terminal VSV-G-tag, C-terminal HA tag and N-terminal FLAG tag to Paar1, but in all cases adding the tag caused a loss of functionality (not unexpected because both the C- and the N-terminus form the base of the conical structure that interacts with the end of VgrG1). We also tried an internal HA tag but that did not work either.

However we would like to note that if Paar1 did interact with a separate effector protein, we would expect to see that protein coming down in all the other complexes that contained Paar1, and we would still expect to see that effector coming down in the Vai1 (immunity protein) co-immunoprecipitation, but we did not.

- TvpAB and TvpB are proposed to be part of the tip complex but their putative role as toxin not been tested. Is there a specific reason for excluding this possibility.

Neither TvpAB or TvpB have ever been observed in the T6SS-dependent secretome, as would be required for a secreted toxin/effector, despite both proteins being readily detectable by mass spectrometry (this observation has been added to the to the text, as noted below).

In agreement with this strong indication that they are not the effector/toxin, in a preliminary experiment shown below, we did not observe any toxicity when TvpAB was expressed in *E. coli* or non-immune *S. marcescens*, either in the cytoplasm or directed to the periplasm by fusion with an N-terminal SufI (Tat) signal peptide. We did not test TvpB because the protein only

contains a pentapeptide repeat domain shared with TvpAB and so is unlikely to be toxic if TvpAB is not.

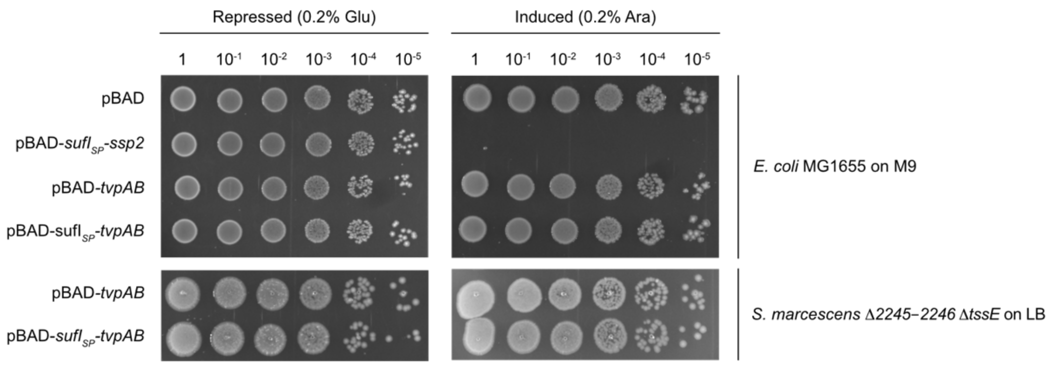

- VgrG1 and PAAR1 are likely secreted by the T6SS but will TvpAB and TvpB also secreted or are they released from the complex at soon as the firing starts and therefore not found outside of the cell.

Neither is observed in the T6SS secretome in multiple studies, despite the proteins being readily detectable by mass spec. Therefore we believe that they are released prior to secretion. We have added a comment to note this in the Discussion.

- Using Alphafold, the authors present very compelling 3D prediction of the complexes formed on the VgrG1 tip but have no experimental validation of their models. One possibility would be to design a couple of mutations at the interface between interacting proteins and assess whether functionality of the T6SS is then disrupted.

In order to validate our AlphaFold models, we carefully designed three variants of TvpC, with up to three mutations at different interfaces with TvpAB, TvpB and Paar1. These mutations, based on the models, would be predicted to disrupt the relevant interactions and impair complex formation. We chose to make mutations in TvpC due to its central position within the pre-complex and also due to its trimeric nature which means that mutating one residue can affect three different faces within the complex. This should thus maximise our chance to observe an impact on T6SS function with the fewest possible amino acid substitutions, to minimise potential impact on protein stability.

We were able to show that the substitutions introduced into our three TvpC variants do indeed result in a loss of VgrG1/Tvp-dependent T6SS activity, and that, for two of them, the variant protein is present at levels comparable to (or higher than) the wild type protein. Therefore these findings provide experimental validation of our AlphaFold model and are included in the revised manuscript as new Figures 7 and EV4. We thank the Reviewer for making this suggestion.

- A recently published work presents the characterization of a TvpAB-DUF2169 homolog in Vibrio parahaemolyticus (Sachar et al., JBC, 2025). In this study the authors indicate that DUF2169 interacts with PAAR-like protein which possesses a non-canonical PIPY domain instead. Does PAAR1 has a PIPY domain?

Sachar et al. refer to DUF4150 domains as PIPY domains (and a separate study has defined DUF4150-containing proteins as one of eight families of PAAR/PAAR-like proteins, PMID

34874775). Paar1 has a DUF4150 domain so yes, it contains a PIPY domain according to the Sachar et al., nomenclature. However, the situation is slightly more nuanced. The interaction between the DUF2169 domain of TvpA/TvpAB and the PAAR-like protein (DUF4150-containing protein) is predominantly through hydrophobic interfaces, but those regions differ between *S. marcescens* and *V. parahaemolyticus*, as highlighted in Appendix Figure S14 and discussed in the text. The β-strand containing the PIPY motif in *V. parahaemolyticus* is VV**PIPY** (with V**PIPY** found also in *P. aeruginosa* and *A. tumefaciens*) and in *S. marcescens* it is PV**P**V**PY**.

We have added a note in the text that these PAAR-like domains are referred to as PIPY domains in the Sachar et al. study.

- The preassembly on TvpC instead of VgrG1 is a novel and intriguing concept. The idea that the TvpAB-TvpB-PAAR1 complex then moves from TvpC onto VgrG1, or that VgrG1 displaces TvpC, is not obvious. Would the authors have hypotheses on this exchange might happen without disrupting the structure of the preformed complex.

We agree with the Reviewer that preassembly on TvpC is a novel and intriguing concept, and that the mechanism behind the transition from the pre-complex to the full complex is not obvious. We hope that future structural and biochemical studies will reveal the details of this transition. Please see our response to Reviewer 3, who also asked for our thoughts about how the exchange might occur, as well as the possible benefit of having a pre-complex. We have revised the relevant section of the discussion to further explain and clarify our proposed model.

Minor comments

- The authors suggest that TvpAB mediates interference between VgrG1 and VgrG2 pathways (lanes 191-192). Could that be indirect. If TvpAB needed for proper VgrG1 tip assembly, then no VgrG1 activity will result in increased VgrG2 activity, as suggested by the authors in previous sections.

If the increased activity observed in the absence of TvpAB was only due to the loss of VgrG1 tip assembly, then the loss of VgrG1 itself (or any other of the Tvp components) would give the same phenotype as loss of TvpAB. However the Δ*tvpAB* mutant shows significantly increased killing activity above the little or no increase in the Δ*vgrG1* mutant (Figure 2E-G), showing that the increased activity cannot simply be down to a loss of competition between VgrG1 and VgrG2. This is noted in the manuscript several lines earlier.

- Lane 102 tssK should be all with italics; lane 175 tss with italics

tssE and *tss* are now all with italics.

- The authors indicate that the genes downstream vai1 does not encode an effector (Lane 420), likely based on the use genetic mutants and competition experiments (Fig. 1). Has the gene been expressed directly expressed, for example in Escherichia coli, to assess toxicity?

Indeed, the conclusion that SMDB11_2246 is not the cognate effector for Vai1 was based on competition assays with deletion mutants, already shown in Figure 1, but also on heterologous expression experiments that we did not show in the initial submission as they were only negative results. However, we have now included toxicity assays using direct expression of

SMDB11_2246 in both *E. coli* MG1655 and the *S. marcescens* susceptible *Δ2245-2246 ΔtssE* strain in Appendix Figure S1. These assays confirm that SMDB11_2246 does not show any toxic activity when expressed in the cytoplasm or the periplasm.

Referee #2:

In this study, the authors characterize a set of Type VI secretion system accessory proteins, termed Tvp complexes, which are required for the assembly of the VgrG1-PAAR1 spike in Serratia marcescens. Although the DUF2169 domain proteins are known to be important for effector delivery and conserved among mang T6SS organisms, this study provided some additional information by proposing that TvpAB, TvpB, and TvpC formed a pre-complex with PAAR1, TvpC was later replaced by VgrG1, forming VgrG1-effector-PAAR1 complex for delivery. Using antibacterial competition assays, immunoprecipitation, mass spectrometry, structural predictions, and phylogenetic analyses, the authors demonstrate that Tvp complexes are evolutionarily conserved.

Although the authors present an interesting model for Tvp protein function, they do not sufficiently support it with experimental evidence. The precise mechanism of VgrG1-associated toxicity and structural validation of the Tvp complexes also remain unclear. Additionally, the introduction should cite more primary references related to key T6SS discoveries, such as the identification of structural components, adaptors, and chaperones.

We have followed the EMBO author guidelines for the introduction: "to provide only the necessary background information, rather than comprise a comprehensive review of the field."

Major issues:

1. The bacterial competition assays demonstrate that wild-type Serratia marcescens Db10 can efficiently kill the Δ2245-2246 ΔtssE strain, and the authors identify SMDB11_2245 as the cognate immunity protein for the VgrG1 effector. However, the manuscript reports successful construction of a Δ2245 mutant in the wild-type background. Given that the T6SS is active under these conditions, one would expect that the Δ2245 mutant should be self-intoxicated by VgrG1 and therefore non-viable. The authors should clarify how this strain was obtained and maintained.

The VgrG1-dependent effector is a periplasmic-acting effector so it will not intoxicate the producing cell itself, because the effector will be incorporated into the T6SS machinery in the cytoplasm and never access the periplasm before or during secretion. It will only intoxicate genetically-identical sibling cells when delivered into their periplasm from the outside by the T6SS. This is why the immunity proteins for periplasmic-acting T6SS effectors are located or exposed in the periplasm (as for Vai1). Importantly, it also means that the Δ*vai1* (Δ2245) immunity mutant in a wild type background is not intrinsically non-viable, and it grows normally in liquid culture where T6SS-mediated delivery into sibling cells does not occur. On solid media, where T6SS delivery between cells can occur, the mutant does grow considerably slower, but it still grows. The VgrG2 delivery pathway is the most efficient pathway in *S. marcescens*, so the effect of the VgrG1-dependent effector on non-immune sibling cells is relatively modest. In competition assays, recovery of the intoxicated *Δ2245Δ2246 ΔtssE* target after 7 hr co-culture

with wild type Db10 is still higher than the starting inoculum, showing that the population can still grow to some degree whilst moderate levels of intoxication are occurring. (Note that the *Δ2245Δ2246 ΔtssE* target strain is perfectly healthy because no inter-sibling intoxication can occur). Therefore, the Δ2245 strain is viable and could be isolated using normal procedures (albeit as a slow-growing mutant) and was subsequently maintained in liquid culture.

The self-toxicity problem arises mainly in a *ΔvgrG2* background, since in this case there is a higher level of delivery of the VgrG1-dependent effector. In this background, the *Δ2245* mutant was very difficult to obtain and all the colonies were either very small/sick, or were healthy due to spontaneous loss of T6SS activity. Even from the very small colonies, only in one case could we identify a *Δ2245ΔvgrG2* mutant which appeared correct, with detectable T6SS activity and able to be restored to healthy colony size by complementation with a plasmid expressing SMDB11_2245. This is the strain that we used in Figure 1E in the original submission, where it was unable to intoxicate the *Δ2245Δ2246 ΔtssE* target. We ascribed this to a loss of competitive fitness or T6SS activity due to its extreme sickness. However, further checking showed that our glycerol stock now streaks out to give normal/healthy colonies rather than the very sick ones we had initially. Thus, it seems that suppressor mutants arise very frequently upon streaking or in the glycerol stock. For that reason, we have decided to remove the *ΔvgrG2 Δ2245* attacker strain from Figure 1E as we cannot be confident in its genotype during the assay and since it provided no useful information in any case, given its sickness.

In addition, the competition assays presented do not include data on the survival of the attacker ("killer") strains. Since differences in growth fitness among the various mutants could confound interpretation of competition outcomes, it is important to provide killer survival data and/or growth curves to confirm that the observed phenotypes are not attributable to differences in bacterial growth.

It is not routine in the field to provide viable counts for the attacker/killer in every co-culture assay (partly since it involves further genetic modification of the attackers). Some labs do, but generally it is because they want to report the competition outcome as a ratio between attacker and target cells at the end of the competition.

The T6SS is not essential and mutants lacking T6SS function do not show impaired growth (likely the opposite). We also note that growth curves are performed in liquid, so they do not represent conditions under which T6SS-mediated interactions occur and would not show any growth impacts due to within-population intoxication. In the current work, we have shown that *tvpAB, tvpB, tvpC, vgrG1* and *paar1* mutants kill *Pseudomonas fluorescens* indistinguishably from the wild type (Figure 2B). Therefore these mutants do not have any significant growth fitness phenotype which affects their ability to kill other cells using the T6SS.

As mentioned above, the *Δ2245* mutant (in a wild type background) does grow more slowly under competition conditions, and this likely explains a very small apparent decrease in activity against the *Δ2245Δ2246 ΔtssE* target, which we have noted in the manuscript (although it still shows a significant killing effect compared with the *ΔtssE* mutant and no statistically significant difference from the wild type). For this strain, and the other two attackers where we do not have evidence of a lack of fitness defect from competitions against *P. fluorescens*, namely *Δ2246* and

Δ2246ΔvgrG2, we have quantified their recovery after mono-culture on solid medium for 7.5 hours, reflecting competition conditions:

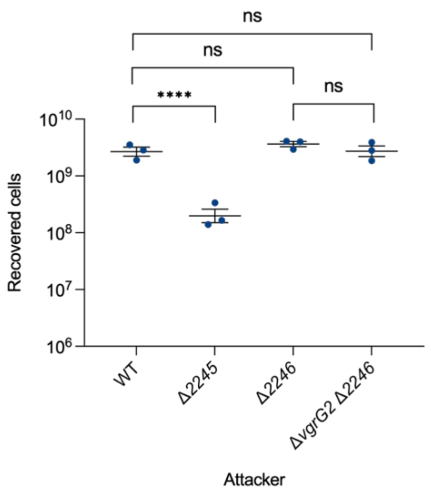

This CFU determination shows that the recovery of both *Δ2246* and *Δ2246ΔvgrG2* is indistinguishable from the wild type. Only the *Δ2245* strain shows a lower recovered CFU, as expected and consistent with reduced growth under intoxication-permissive conditions. We have not included this data in the manuscript because none of these mutants show a significant deficiency in activity which needs to be explained.

2. The manuscript does not establish whether the observed VgrG1-associated toxicity is due to VgrG1 itself or to an unidentified VgrG1-dependent effector. To clarify this, the authors should test whether VgrG1 expression in strains lacking SMDB11_2245 and with an inactive T6SS, or in a heterologous host such as E. coli, results in toxicity. Complementation with SMDB11_2245 under these conditions could further confirm its role as the cognate immunity protein. If no intrinsic toxicity of VgrG1 is observed, it would be important to re-examine the existing VgrG1 pull-down mass spectrometry data, as well as the previously published secretome datasets from this group, to identify potential unannotated effectors that may account for the observed phenotype.

We have done many experiments to look for VgrG1 toxicity upon heterologous expression (as noted in the original manuscript), all the things suggested above and more, but they all failed to show specific toxicity associated with VgrG1. They include VgrG1 full-length or just its C-terminal extension with various lengths, different expression organisms (*E. coli*, *S. marcescens*, *Salmonella*), different signal peptides to direct the protein to the periplasm, etc.

To show examples of these negative results, we have now included two sets of heterologous expression assays in the revised manuscript (new Appendix Fig. S2). We did observe toxicity when the VgrG1 C-terminal domain (VgrG1-CTD) was expressed in *E. coli* with $OmpA_{SP}$ to target it for export to the periplasm, but this was only an artefact due to a deleterious effect on the essential Sec export machinery. Indeed, as explained in the revised text, this same construct was not toxic when expressed in *S. marcescens* and we could avoid this toxicity in *E. coli* by replacing $OmpA_{SP}$ with $SufI_{SP}$ which is recognised by the Tat export machinery. Thus, unfortunately, this approach failed to validate VgrG1 toxicity, explaining why we used alternative

experiments (Fig. 1G). We believe that somehow delivery by the T6SS into the periplasm is essential for toxicity and artificial expression and periplasmic targeting by export machineries cannot recapitulate this.

Regarding potential unannotated genes which might encode a VgrG1-dependent effector cognate to the Vai1 immunity protein: To the best of our knowledge and current literature, immunity proteins are always encoded next to the effector gene. We have checked the locus around *vgrG1, vai1* and *SMDB11_2246* genes very carefully for possible unannotated effectors and not found a candidate. Indeed the start codon for *vai1/SMDB11_2245* is only a few bases downstream of the stop codon of *vgrG1*, an arrangement very typical for cognate effector-immunity genes. We have also re-searched the VgrG1 pull-down mass spectrometry data against a custom all-ORFs database and retrieved no additional hits. We have included an extra comment in the manuscript regarding the lack of candidate unannotated ORFs.

3. In Figure 3, all co-immunoprecipitation experiments are performed in the native strain background, which does not provide direct evidence for protein-protein interactions. To strengthen the proposed model, in vitro assays such as pull-down experiments with purified proteins, size-exclusion chromatography, or native PAGE should be conducted. Since the authors suggest that TvpAB, TvpB, and TvpC form a pre-complex with PAAR1 that is subsequently replaced by VgrG1, it would be particularly informative to test whether adding VgrG1 to a pre-assembled TvpAB-TvpB-TvpC-PAAR1 complex can displace TvpC in vitro.

We disagree with the comment that the way we have performed our co-IP/pull-down experiments does not provide direct evidence for protein-protein interactions. If we had done the IP with one protein and then detected the second by western blotting, then, indeed, we could not conclude that the second directly interacts with the first. In contrast, we have used the bait protein to isolate the entire – native – complex, and then detected all components of that complex by mass spectrometry. The outcome was very clear: all proteins enriched are reported in the figures, there are no 'hidden' proteins, and the members of our complexes are detected much more strongly than other, false-positive proteins. Thus there must be direct protein-protein interactions within these complexes (which only contain four proteins).

We do agree that we have not shown individual pair-wise interactions between pairs of proteins, but note that this would not necessarily work *in vitro* either if there are mutual stabilisation requirements among the proteins (*i.e.* if two proteins are only stable in a complex and that complex interacts with a third protein, you can't conclude either of the first two is directly interacting with the third). Indeed, we have indications that this is the case, which is of course consistent with direct protein-protein interactions. We already note in the manuscript that we observe reduced levels of TvpAB in the absence of TvpC, and have separate data showing that levels of TvpAB become undetectable in the absence of TvpB.

Yes, we always intended to validate our IP results and models *in vitro*. We have spent many years trying to purify these proteins, singly and in combinations, with many different tags, conditions and approaches, with little success. Proteins are insoluble and/or prone to degradation, cannot be refolded, etc. The table below summarises all these efforts we made to find a way to express these proteins, alone or in complex, in a soluble manner:

	Plasmid name & combination	Plasmid 1		Plasmid 2		Strains and Induction temperature												Main conclusion
						BL21 DE3			BL21 AI		C41/C43 DE3			Shuffle			Arctic	
		MCS1	MCS2	MCS1	MCS2	37°C	30°C	16°C	30°C	16°C	37°C	30°C	16°C	37°C	30°C	16°C	13°C	
Single proteins	pSC2033	His-VgrG1						✓ (orange)										50 kDa degradation product soluble, full-length insoluble
	pSC2021	His-VgrG1 Cter domain						✓ (orange)		✓						✓	✓	insoluble
	pSC1933	His-TvpAB					✓		✓	✓ (orange)			✓			✓	✓ (orange)	insoluble ++
	pSC1976	GST-TvpAB						✓										
	pSC2020	His-TvpAB DUF2169 domai						✓		✓						✓	✓	
	pSC1930	His-TvpB				✓ (orange)	✓		✓	✓			✓ (orange)			✓		insoluble +++, refractory to refolding
	pSC1949	MBP-TvpB						✓					✓				✓	
	pSC1931	His-TvpC					✓	✓ (orange)	✓	✓			✓			✓ (orange)	✓	partially soluble but precipitation
	pSC1950	MBP-TvpC						✓ (orange)										
	pSC1975	GST-TvpC					✓	✓				✓	✓					
	pSC1932	His-Paar1					✓	✓ (orange)	✓	✓			✓			✓	✓ (orange)	insoluble
	pSC1971	MBP-Paar1					✓ (orange)											
	pSC1972	GST-Paar1					✓	✓				✓						
Two proteins	pSC2009	His-TvpC	Paar1					✓ (orange)										no improvement compared to single proteins
	pSC1937	His-TvpAB	VgrG1					✓										
	pSC2014	His-TvpAB	TvpB					✓		✓						✓	✓	
Full Complex	pSC1937 + pSC1934	His-TvpAB	VgrG1	His-TvpB	Paar1	✓	✓	✓			✓	✓	✓	✓	✓	✓		
Pre Complex	pSC2014 + pSC2009	His-TvpAB	TvpB	His-TvpC	Paar1	✓	✓	✓			✓	✓	✓	✓	✓	✓		

(orange) conditions where purification was attempted

Furthermore, when analyzing the co-immunoprecipitated proteins by mass spectrometry, it would be useful to clarify whether other conserved T6SS components were enriched or differentially detected.

This information is already present in Figure 3, especially if cross-referenced to Figure 1A. Among the limited number of other proteins enriched in the IP fractions, no other T6SS components were detected. We have now also added an explicit statement to the text to make this point clearer for the reader.

4. A substantial portion of the manuscript is devoted to structural predictions of the proposed complexes, but no experimental validation is provided. If obtaining the structure of the entire complex or individual proteins is technically challenging, an alternative approach would be to introduce mutations at the predicted protein-protein interfaces and assess their impact on complex formation and T6SS activity. Such mutational analyses would provide important functional validation of the structural models and significantly strengthen the conclusions.

This suggestion was also proposed by Reviewer 1 (please see above). We have introduced several mutations in TvpC at different predicted interfaces with other components of the pre-complex and shown their impact on Tvp-dependent T6SS activity (new Fig. 7 and Fig. EV4), thereby functionally validating the structural models. We thank the reviewer for their suggestion as the results of this additional experiment strengthen our conclusions.

Minor comments:

1. The expression levels of the complementation plasmids should be verified.

It is not clear to us what the Reviewer is requesting here. The same plasmid, promoter and ribosome binding site was used for each complementation construct, so the plasmid copy number and level of gene expression will be the same across all of them. We don't have antibodies to detect the level of each protein compared with the chromosomal expression level. But complementation was successful in all cases, allowing us to rule out the possibility of polar effects or secondary mutations as intended, so it is unclear why the Reviewer feels that checks on the plasmids (or protein levels) are needed.

2. In Figure 2c, a cytoplasmic protein control should be included to serve as a loading control and to verify equal cell lysis across samples.

Figure 2C now contains new blots where they cytoplasmic control protein, EF-Tu, was also detected in addition to Hcp.

Referee #3:

In this article Monlezun, Earl and Coulthurst describe T6SS spike assemblies involving the additional components called Tvp, in addition to structural VgrG and PAAR components that form a canonical spike complex. They provide experimental evidence that Serratia marcescens assembles multiple complexes involving the PAAR, Tvp proteins and VgrG1. The later seems to be mutually exclusive with TvpC protein which is predicted to mimic the OB-Fold-gp5 moiety of

VgrG. They demonstrate that previously uncharacterized Tvp components are necessary for functionality of the final VgrG1 spike assembly. Authors also further predict a range of spike architectures involving different sets of Tvp partners across wide range of bacterial species. The manuscript is novel and interesting, it is written with great precision, but it is not easy to follow. In particular, the second part, where structures are modelled from different organisms and multiple versions of assemblies appear. The only thing that is universal but modular across these systems are protein domains, and for the most part I would consider referring to them by domains rather than mixing it up with names.

We understand that the multiple versions of T6SS/Tvp assemblies could be difficult to follow. However, we do not think that referring to protein domains would make the reading any easier because some proteins have multiple domains and the same domain can be in two different proteins. This is the case, for example, for TvpAB in *S. marcescens*. This is composed of the DUF2169 domain, which is abbreviated by 'A' in the names "TvpAB" or "TvpA" for proteins containing only this domain; and of the pentapeptide repeat (PPR) domain, which is abbreviated by 'B' in the names "TvpAB" or "TvpB", the protein in *S. marcescens* which contains only this PPR domain. So to sum-up our nomenclature, if the DUF2169 domain is present, the name contains the letter A and if the PPR is present, the name contains the letter B.

The experimental part of the work is solid and brings a lot of new information carefully demonstrated in native settings (i.e. in Serratia, in inter-bacterial competition, experimented with multiple bacterial species). However, the second part based almost entirely on AlphaFold modelling is highly speculative and less synthesized. In general, authors suggest existence of two complexes with PAAR enclosed by DUF2169 and PPR domains. One sits on shorter mimicry version of VgrG (TvpC) and other on true VgrG. What is not clear is how this transition occurs, and what is the benefit or necessity of forming the first complex overall. Can authors really claim it is two-step assembly? The only argument is that without the TvpC, the second (functional?) complex is not assembled, but what is truly missing to support these claims is the evidence that the first complex can transition to the second one.

Importantly, we have to consider that extreme rearrangement would have to be made to extract the TvpC and replace it by VgrG. This to me sounds like a very complicated step to imagine, especially in the context of complexes including PPR domains that seems to have extensive interactions with TvpC in the "pre-complex".

We agree with the Reviewer that the mechanism behind the transition from the pre-complex to the full complex, and why this step is needed or beneficial, is not yet clear.

We describe a model in the manuscript whereby the T6SS baseplate cannot assemble effectively around a VgrG protein containing a longer β-prism due to interference with the membrane complex, and therefore initially assembles on the pre-complex containing TvpC, with a shorter, classical β-prism. Whilst only a speculative model, it is consistent with our observation that the baseplate protein TssK interacts with VgrG2 (shorter β-prism) and TvpC, but not VgrG1 (longer β-prism), according to bacterial two-hybrid assay. Additionally, the fact that we cannot detect a VgrG1-TvpAB-TvpB-Paar1 complex in the absence of TvpC indicates that the TvpAB-TvpB-Paar1 'cap' cannot assemble *de novo* on VgrG1, perhaps again because of the

longer β-prism, but must first form on the shorter TvpC β-prism. Then this intact cap complex, likely within the baseplate, can accept VgrG1.

We believe that our data showing that: (1) TvpC-TvpAB-TvpB-Paar1 complexes can form in the absence of VgrG1; (2) VgrG1-TvpAB-TvpB-Paar1 complexes cannot form in the absence of TvpC, i.e. unless TvpC-TvpAB-TvpB-Paar1 complexes can also form; and (3) an assembly containing VgrG1and Paar1 is what is ultimately required for a fully-assembled, firing competent T6SS (PMID: 27352036); can only be explained by a 'two-step' process, where TvpC-TvpAB-TvpB-Paar1 forms first and is required for subsequent formation of VgrG1-TvpAB-TvpB-Paar1. However we agree that we have not directly demonstrated replacement of TvpC by VgrG1 in the same complex and have tempered several statements in the manuscript accordingly.

In terms of how an exchange from TvpC to VgrG1 might occur, we can hypothesize that once the pre-complex is fitted within the baseplate cavity, external proteins in this pre-complex, namely TvpAB-TvpB, will make new interactions with baseplate components which might rearrange the pre-complex, or at least loosen the interaction with TvpC so that the protein can exit the complex. The TvpC variants we designed showed that making relatively few changes (mutating one to three residues) in the interfaces between TvpC and TvpAB/B or Paar1 could be sufficient to prevent the interaction and cause a loss of Tvp functionality. This suggests it is not impossible to 'break' the interaction between TvpC and the other components, allowing for TvpC to leave and be replaced by VgrG1.

We have revised the relevant section of the discussion to further explain and clarify our proposed model as well as ensured that statements around the inferred replacement of TvpC with VgrG1 are suitably qualified, as above.

Other major concerns:

Figure 1 b to e - it should be indicated if the observed differences of measurements are statistically significant. For example, this is important for the claims regarding the differences between WT and delta tssE attacker conditions. Figure 2 a-b, d-g - statistical significance of observed differences should also be provided.

We have added statistics as requested (Figures 1, 2, 7, EV1).

Figure 1 d - How can authors explain that in the background of target strain complemented with 2246 there is no more visible difference between the WT and delta tssE attacker conditions? It also seems that complementation with 2245 still presents slight difference between WT vs delta tssE or delta VgrG2 Could this be the difference driven by the missing 2246? What do we know about 2246?

For the question about complementation with 2245, the Δ*tssE* mutant is apparently killing slightly 'better' than the wild type, not the other way round, so this would not indicate any failure of 2245 to complement/protect the target. This 'slight difference' is not, in any case, statistically significant.

In the case of the 2246 complementation experiment, we do not know why the relatively small effector-dependent killing window has disappeared for the wild type attacker. Plasmid complementation is not always perfect because overexpression of specific proteins sometimes

leads to artefactual effects or burden on the cell, which may well be the case here. However, it does not affect the main conclusion of the figure, as the Δ*vgrG2* attacker is still very efficient in killing the Δ*2245*Δ*2246* target when 2246 is expressed (in contrast with the complete loss of killing when 2245 is expressed in this target). Therefore 2246 is not the immunity protein protecting cells against the VgrG1-dependent effector (consistent with the Δ*2246* mutant showing no susceptibility to the effector, Figure EV1C)

SMDB11_2246 is a 166 amino acid integral membrane protein with four predicted transmembrane helices and no conserved domains identified. It is not always present in T6SS clusters with 2245 and we think it is likely to be an orphan immunity protein.

Page 7, line 188 - I find this activating effect of TvpAB confusing. It could be relevant to test the double TvpAB/VgrG2 mutant against these other strains as well. It would be expected, that this mutant should behave like in competition with Pseudomonas fluorescence. This would maybe clarify if it has indeed something to do with the VgrG2 pathway?

This will not be informative as the Δ*tvpAB*Δ*vgrG2* mutant has a non-functional T6SS.

Regarding the prediction of multimers and complexes by AlphaFold, the important score to provide is iPTM. Are we sure that these models were predicted using the correct stoichiometry? How was this chosen?

ipTM values were already provided in Supplementary Table 4 (now Appendix Table S4). We have now cited them in the text alongside pLDDT.

Multiple experimental structural studies have shown that VgrG proteins are trimers (including the β-prism being an intrinsically trimeric structure with the three subunits intertwined), and that they are topped by a monomeric PAAR/PAAR-like protein or domain. TvpC proteins are essentially short VgrG proteins, with the same OB-fold and β-prism domains, hence those should also be trimers. Therefore all our predictions were based on 3xVgrG1, 1xPAAR and 3xTvpC. For TvpAB andTvpB we did not have prior information before performing our AlphaFold predictions, but complexes containing one TvpAB and one TvpB gave good ipTM and pTM values and biologically 'sensible' structures upon visual inspection. Increasing the copy number of either protein results in structures that have substantially lower ipTM and pTM values and are not (or less) sensible. Subsequently, the experimental structure of a TvpA protein showed it to be a monomer, which is consistent with our model, and our new experimental validation data provides further confidence. Therefore we believe we believe our models represent the correct, or at least by far the most likely, stoichiometry.

Line 315 - I think this is highly speculative to talk about re-arrangements to a level of providing rmsd values, just by comparing multiple AlphaFold multimer models.

We have removed both the RMSD values and the comment about rearrangement in this section.

Minor remarks:

In line with previous publications and since it is an abbreviation coming from amino acid motif, it might be better to maintain the entire name PAAR in capital letters,

We do use PAAR, when talking about the domain or protein family. When talking about the specific protein SMDB11_2250, we use Paar1.

Page 2, lane 7 - "anchoring of the membrane complex" - the term "anchoring" for membrane proteins usually means a class of membrane proteins that are not integral but anchored by lipidic anchors or via other proteins. In this nomenclature the T6SS membrane complex comprises integral membrane proteins, hence this expression can be misleading.

We agree that using the word 'anchoring' for integral membrane proteins might be misleading, so it has been changed for 'insertion'.

Page 3, lane 69 - it is not very clear what authors mean by "requirement to occupy the cavity around the spike in the baseplate" - this likely refers to requirement for assembly or function of T6SS which is elaborated in discussion? But here it should be better explained and referenced.

This comment is within a summary sentence of our main conclusions at the end of the Introduction. As the reviewer correctly identifies, we are referring to a requirement for the cavity around the spike within the baseplate to be occupied in order for the T6SS to assemble and therefore function, which we propose is one of the roles fulfilled by the Tvp complex. As the reviewer notes, this is elaborated (and referenced) in the Discussion, which we feel is the appropriate place for the full explanation and references. However we thank the Reviewer for pointing out that the current wording is not very clear, so we have reworded the sentence to improve its clarity, particularly when read before the full explanation in the Discussion.

Lane 77 - at first mention of delta tssE background, the phenotype and the rationale of this background should be provided.

The phenotype and rationale have now been explained. ("We generated a mutant lacking both genes in a T6SS-inactive background (Δ*tssE*), strain Δ*2245-2246* Δ*tssE*. If either SMDB11_2245 or SMDB11_2246 represented immunity proteins, this strain would be susceptible to T6SS-dependent intoxication by the wild type. In the case that either was an immunity protein but the other was not the cognate effector, a Δ*2245-2246* mutant would be subject to self-intoxication between sibling cells, resulting in lethality or a severe fitness defect. If the hypothetical effector acted from within the periplasm of intoxicated cells, as appeared likely due to the predicted localisation of SMDB11_2245 (see below), then inactivation of the T6SS (Δ*tssE*) would prevent any self-intoxication since the effector would never access the periplasm.")

Lane 143 - for consistency and clarity it would be better if Db10 would be called "WT" or "Db10 WT"

This has been adjusted in the text.

Page 6, Lane 176 - I think the reference 49 is called before many other references. Please review the order of references accordingly.

References are now cited in text by author and year of publication and listed alphabetically in the References section as requested in EMBO Journal guidelines so the problem is solved.

Page 7, line 178 - what was the difference with "initial co-culture assays" provided in Supplementary Figure 3, as compared to those presented in Figure 2b? without proper explanation this brings in some confusion.

Formally there was no difference in the competition settings between Suppl. Fig. 3 and Fig. 2B, but they were two different experiments (performed five years apart). We agree that it might be confusing, so we have decided to remove the extra competition in Suppl. Fig. 3.

Page 9, line 264 - it might be overstatement to call these VgrG's non-essential. I see what the authors imply, since they do not appear alone, but it might be more cautious to use term "alternative"?

We agree that the word ‘essential’ was too strong, we have now softened the sentence using ‘alternative’ as suggested by the reviewer.

Supplementary Figure 10 could also be annotated on the figure, not only in legend to simplify the interpretation.

The annotation has been added onto the figure, which is now called ‘Appendix Figure S8’.

Line 308 - what do authors exactly mean by "it was unable to generate a prediction"?

The program (AlphaFold) crashed rather than being able to successfully complete a prediction. We have clarified the sentence

Line 372 - are there sufficient evidence to claim about the evolution towards increased complexity? This implies the directionality of the evolution, although these classes might have a common ancestor and evolve separately?

We have removed the sentence discussing evolutionary trend as the Reviewer is correct that we have no direct evidence for the evolutionary path to the different complexes. We thank them for highlighting this.

1st Revision - Editorial Decision **27th Apr 2026**

Dear Sarah,

Thank you for submitting a revised version of your manuscript to The EMBO journal. Your study has now been seen by two of the original referees, who find that their previous concerns have been sufficiently addressed. There now remain only a few formatting aspects points that need to be addressed before I can extend the official acceptance of the manuscript:

1. Please note that it is The EMBO Journal policy for the transcript of the editorial process (containing referee reports and your response letters) to be published as an online supplement to each paper. If you should prefer removal of any referee-only figures included in the point-by-point response(s), e.g. because they may still be used for future publication or because they have been reproduced from published work by others, please do let us know immediately via response email.
More information is available here: https://www.embopress.org/transparent-process#Review_Process
2. The headings and order of the sections in the manuscript text should be as follows: Abstract / Introduction / Results / Discussion / Methods / Data Availability / Acknowledgements / Disclosure and Competing Interests Statement / References / Figure Legends / (Main Tables with legends if applicable) / Expanded View Figure Legends.
3. Please check that the funding information is correct and identical both in the manuscript and our online system. Currently, Wellcome Technology Platform Award [097945/B/11/Z] is missing in the online system - please check if it should be included in the list of the funders that will be linked in Pubmed.
4. CRediT has replaced the traditional author contributions section because it offers a systematic, machine-readable author contributions format that allows for more effective research assessment. Please remove the Authors Contributions from the manuscript and use the free text boxes beneath each contributing author's name in our online submission system to add specific details on the author's contribution. More information is available in our guide to authors.
5. Please remove DOIs from the published articles in the reference lists for the main manuscript and Appendix, leaving it in only for preprints and datasets.
6. In the Appendix, please add page numbers in the Table of Contents and remove the author list on the title page.
7. Figure panel 8D is not mentioned in the figure legend.
8. Appendix Table S3 is not mentioned in the manuscript text, please add the corresponding callout.
9. Please correct the callout for Appendix Table S4 (currently Table S4).
10. During our routine image integrity checks, we noted that image panels in Appendix Figure S1A appear reused, despite representing different experimental conditions. Please check and correct.
11. Our data editors request inclusion of the exact p values in the following figure legends or figures themselves: 1B-E; 2A, B, D, E, F, G; 7A, B; EV1 A, B.
12. Papers published in The EMBO Journal are accompanied online by a 'Synopsis' to enhance discoverability of the manuscript. It consists of A) a short (1-2 sentences) summary of the findings and their significance, B) 3-4 bullet points highlighting key results (the highlights currently included in the manuscript can be used here) and C) a synopsis image that is 550 x 300 pixels large (width x height, jpeg or png format). You can either show a model or key data in the synopsis image. Please note that the image size is rather small and that text needs to be readable at the final size.

Please let me know if you have any questions regarding any of these points. You can use the link below to upload the revised files.

Thank you again for giving us the chance to consider your manuscript for The EMBO Journal. I look forward to receiving the final version!

With best wishes,

Ieva

Ieva Gailite, PhD
Senior Scientific Editor
The EMBO Journal
Meyerhofstrasse 1
D-69117 Heidelberg
Tel: +4962218891309
i.gailite@embojournal.org

We realize that it is difficult to revise to a specific deadline. In the interest of protecting the conceptual advance provided by the work, we recommend a revision within 3 months (26th Jul 2026). Please discuss the revision progress ahead of this time with the editor if you require more time to complete the revisions. Use the link below to submit your revision:

https://emboj.msubmit.net/cgi-bin/main.plex

Referee #1:

This is a paper establishing novel concepts on the assembly of the T6SS and which I have previously reviewed. The authors have been doing an excellent work to try addressing all my previous comments including performing technically challenging experiments. Even when the data were not obtained or conclusive, the authors discussed the outcome in the paper. In some cases, such as the validation of the AlphaFold prediction of the VgrG1 tip structure using mutagenesis approach, the data obtained are a clear added value.

Referee #3:

In the revised manuscript authors have responded the critiques of all reviewers. Of note, these critiques are very similar. While strongest critiques regarding structural validation of the model, and toxicity of the putative effector remain to be addressed, I acknowledge that experiments with membrane proteins and putative membrane targeting peptides are very challenging and it's an immense project on its own. Nevertheless, authors provided toxicity assays supporting lack of toxicity of other candidates that could potentially be considered toxins. Additionally, they have provided experimental data supporting the protein interaction model. And yet again, regarding further in vitro confirmation of interactions or structural analyses, it is extremely challenging. Authors provided the extensive list of efforts that have been done to express and purify proteins of these complexes.
I thank the authors for carefully addressing the remarks regarding the clarity of the text and interpretation of data. As authors seem to be confident with their model, I have no further remarks which could be technically feasible at this stage.

All editorial and formatting issues were resolved by the authors.

Dear Sarah,

Thank you for incorporating the final formatting requests in the manuscript. I am now pleased to inform you that your manuscript has been accepted for publication. Congratulations with a great study!

Before we forward your manuscript to our publishers, we would like to propose some edits in the synopsis text that you kindly provided. I have also selected a short blurb that will accompany the title of your manuscript in our online table of contents. Please take a look at the proposed text changes in the attached text file and let me know if any corrections are needed.

Your manuscript will be processed for publication by EMBO Press. It will be copy edited and you will receive page proofs prior to publication. Please note that you will be contacted by Springer Nature Author Services to complete licensing and payment information.

You may qualify for financial assistance for your publication charges - either via a Springer Nature fully open access agreement or an EMBO initiative. Check your eligibility: https://link.springer.com/journal/44318/how-to-publish-with-us

Should you be planning a Press Release on your article, please get in contact with embo_production@springernature.com as early as possible in order to coordinate publication and release dates.

If you have any questions, please do not hesitate to contact the Editorial Office or me directly. Thank you for this interesting contribution to The EMBO Journal!

Best wishes,

Ieva

Ieva Gailite, PhD
Senior Scientific Editor
The EMBO Journal
Meyerhofstrasse 1
D-69117 Heidelberg
Tel: +4962218891309
i.gailite@embojournal.org

** Click here to be directed to your login page: https://emboj.msubmit.net

EMBO Press Author Checklist

Corresponding Author Name: Sarah Coulthurst
Journal Submitted to: The EMBO Journal
Manuscript Number: EMBOJ-2025-122266

USEFUL LINKS FOR COMPLETING THIS FORM
The EMBO Journal - Author Guidelines
EMBO Reports - Author Guidelines
Molecular Systems Biology - Author Guidelines
EMBO Molecular Medicine - Author Guidelines

Reporting Checklist for Life Science Articles (updated January
This checklist is adapted from Materials Design Analysis Reporting (MDAR) Checklist for Authors. MDAR establishes a minimum set of requirements in transparent reporting in the life sciences (see Statement of Task: 10.31222/osf.io/9sm4x). Please follow the journal's guidelines in preparing your
Please note that a copy of this checklist will be published alongside your article.

Abridged guidelines for figures
1. Data
The data shown in figures should satisfy the following conditions:
- the data were obtained and processed according to the field's best practice and are presented to reflect the results of the experiments in an accurate and unbiased manner.
- ideally, figure panels should include only measurements that are directly comparable to each other and obtained with the same assay.
- plots include clearly labeled error bars for independent experiments and sample sizes. Unless justified, error bars should not be shown for technical
- if n<5, the individual data points from each experiment should be plotted. Any statistical test employed should be justified.
- Source Data should be included to report the data underlying figures according to the guidelines set out in the authorship guidelines on Data

2. Captions
Each figure caption should contain the following information, for each panel where they are relevant:
- a specification of the experimental system investigated (eg cell line, species name).
- the assay(s) and method(s) used to carry out the reported observations and measurements.
- an explicit mention of the biological and chemical entity(ies) that are being measured.
- an explicit mention of the biological and chemical entity(ies) that are altered/varied/perturbed in a controlled manner.
- the exact sample size (n) for each experimental group/condition, given as a number, not a range;
- a description of the sample collection allowing the reader to understand whether the samples represent technical or biological replicates (including how many animals, litters, cultures, etc.).
- a statement of how many times the experiment shown was independently replicated in the laboratory.
- definitions of statistical methods and measures:
  - common tests, such as t-test (please specify whether paired vs. unpaired), simple χ2 tests, Wilcoxon and Mann-Whitney tests, can be unambiguously identified by name only, but more complex techniques should be described in the methods section;
  - are tests one-sided or two-sided?
  - are there adjustments for multiple comparisons?
  - exact statistical test results, e.g., P values = x but not P values < x;
  - definition of 'center values' as median or average;
  - definition of error bars as s.d. or s.e.m.

Please complete ALL of the questions below.
Select "Not Applicable" only when the requested information is not relevant for your study.

Materials

Newly Created Materials	Information included in the manuscript?	In which section is the information available? (Reagents and Tools Table, Materials and Methods, Figures, Data Availability Section)
New materials and reagents need to be available; do any restrictions apply?	Yes	Materials and Methods, Appendix tables

Antibodies	Information included in the manuscript?	In which section is the information available? (Reagents and Tools Table, Materials and Methods, Figures, Data Availability Section)
For **antibodies** provide the following information:<br>- Commercial antibodies: RRID (if possible) or supplier name, catalogue number and or/clone number<br>- Non-commercial: RRID or citation	Yes	Reagents and Tools Table, Materials and Methods

DNA and RNA sequences	Information included in the manuscript?	In which section is the information available? (Reagents and Tools Table, Materials and Methods, Figures, Data Availability Section)
**Short novel DNA or RNA including primers, probes**: provide the sequences.	Yes	Appendix Table S3

Cell materials	Information included in the manuscript?	In which section is the information available? (Reagents and Tools Table, Materials and Methods, Figures, Data Availability Section)
**Cell lines:** Provide species information, strain. Provide accession number in repository **OR** supplier name, catalog number, clone number, and/**OR** RRID.	Not Applicable	
**Primary cultures:** Provide species, strain, sex of origin, genetic modification status.	Not Applicable	
Report if the cell lines were recently **authenticated** (e.g., by STR profiling) and tested for mycoplasma contamination.	Not Applicable	

Experimental animals	Information included in the manuscript?	In which section is the information available? (Reagents and Tools Table, Materials and Methods, Figures, Data Availability Section)
**Laboratory animals or Model organisms:** Provide species, strain, sex, age, genetic modification status. Provide accession number in repository **OR** supplier name, catalog number, clone number, **OR** RRID.	Not Applicable	
**Animal observed in or captured from the field:** Provide species, sex, and age where possible.	Not Applicable	
Please detail **housing and husbandry conditions**.	Not Applicable	

Plants and microbes	Information included in the manuscript?	In which section is the information available? (Reagents and Tools Table, Materials and Methods, Figures, Data Availability Section)
**Plants:** provide species and strain, ecotype and cultivar where relevant, unique accession number if available, and source (including location for collected wild specimens).	Not Applicable	
**Microbes:** provide species and strain, unique accession number if available, and source.	Yes	Appendix Table S1

Human research participants	Information included in the manuscript?	In which section is the information available? (Reagents and Tools Table, Materials and Methods, Figures, Data Availability Section)
If collected and within the bounds of privacy constraints report on age, sex and gender or ethnicity for all study participants.	Not Applicable	

Core facilities	Information included in the manuscript?	In which section is the information available? (Reagents and Tools Table, Materials and Methods, Figures, Data Availability Section)
If your work benefited from core facilities, was their service mentioned in the acknowledgments section?	Yes	Acknowledgments Section

Design

Study protocol	Information included in the manuscript?	In which section is the information available? (Reagents and Tools Table, Materials and Methods, Figures, Data Availability Section)
If study protocol has been **pre-registered, provide DOI in the manuscript**. For clinical trials, provide the trial registration number **OR** cite DOI.	Not Applicable	
Report the **clinical trial registration number** (at ClinicalTrials.gov or equivalent), where applicable.	Not Applicable	

Laboratory protocol	Information included in the manuscript?	In which section is the information available? (Reagents and Tools Table, Materials and Methods, Figures, Data Availability Section)
Provide DOI OR other citation details if **external detailed step-by-step protocols** are available.	Not Applicable	

Experimental study design and statistics	Information included in the manuscript?	In which section is the information available? (Reagents and Tools Table, Materials and Methods, Figures, Data Availability Section)
Include a statement about **sample size** estimate even if no statistical methods were used.	Yes	Materials and Methods
Were any steps taken to minimize the effects of subjective bias when allocating animals/samples to treatment (e.g. **randomization procedure**)? If yes, have they been described?	Not Applicable	
Include a statement about **blinding** even if no blinding was done.	Yes	Materials and Methods
Describe **inclusion/exclusion criteria** if samples or animals were excluded from the analysis. Were the criteria pre-established?<br><br>If sample or data points were omitted from analysis, report if this was due to attrition or intentional exclusion and provide justification.	Not Applicable	
For every figure, are **statistical tests** justified as appropriate? Do the data meet the assumptions of the tests (e.g., normal distribution)? Describe any methods used to assess it. Is there an estimate of variation within each group of data? Is the variance similar between the groups that are being statistically compared?	Yes	Materials and Methods

Sample definition and in-laboratory replication	Information included in the manuscript?	In which section is the information available? (Reagents and Tools Table, Materials and Methods, Figures, Data Availability Section)
In the figure legends: state number of times the experiment was **replicated** in laboratory.	Yes	Figure legends
In the figure legends: define whether data describe **technical or biological replicates**.	Yes	Figure legends

Ethics

Ethics	Information included in the manuscript?	In which section is the information available? (Reagents and Tools Table, Materials and Methods, Figures, Data Availability Section)
Studies involving **human participants**: State details of **authority granting ethics approval** (IRB or equivalent committee(s), provide reference number for approval.	Not Applicable	
Studies involving **human participants**: Include a statement confirming that **informed consent** was obtained from all subjects and that the experiments conformed to the principles set out in the WMA Declaration of Helsinki and the Department of Health and Human Services Belmont Report.	Not Applicable	
Studies involving **human participants:** For publication of **patient photos**, include a statement confirming that consent to publish was obtained.	Not Applicable	
Studies involving experimental **animals**: State details of **authority granting ethics approval** (IRB or equivalent committee(s), provide reference number for approval. Include a statement of compliance with ethical regulations.	Not Applicable	
Studies involving **specimen and field samples:** State if relevant **permits** obtained, provide details of authority approving study; if none were required, explain why.	Not Applicable	

Dual Use Research of Concern (DURC)	Information included in the manuscript?	In which section is the information available? (Reagents and Tools Table, Materials and Methods, Figures, Data Availability Section)
Could your study fall under dual use research restrictions? Please check biosecurity documents and list of **select agents and toxins** (CDC): https://www.selectagents.gov/sat/list.htm	Not Applicable	
If you used a select agent, is the security level of the lab appropriate and reported in the manuscript?	Not Applicable	
If a study is subject to dual use research of concern regulations, is the name of the **authority granting approval and reference number** for the regulatory approval provided in the manuscript?	Not Applicable	

Reporting

The MDAR framework recommends adoption of discipline-specific guidelines, established and endorsed through community initiatives. Journals have their own policy about requiring specific guidelines and recommendations to complement MDAR.

Adherence to community standards	Information included in the manuscript?	In which section is the information available? (Reagents and Tools Table, Materials and Methods, Figures, Data Availability Section)
State if relevant guidelines or checklists (e.g., **ICMJE, MIBBI, ARRIVE, PRISMA**) have been followed or provided.	Not Applicable	
For **tumor marker prognostic studies,** we recommend that you follow the **REMARK** reporting guidelines (see link list at top right). See author guidelines, under 'Reporting Guidelines'. Please confirm you have followed these guidelines.	Not Applicable	
For **phase II and III randomized controlled trials**, please refer to the **CONSORT** flow diagram (see link list at top right) and submit the CONSORT checklist (see link list at top right) with your submission. See author guidelines, under 'Reporting Guidelines'. Please confirm you have submitted this list.	Not Applicable	

Data Availability

Data availability	Information included in the manuscript?	In which section is the information available? (Reagents and Tools Table, Materials and Methods, Figures, Data Availability Section)
Have **primary datasets** been deposited according to the journal's guidelines (see 'Data Deposition' section) and the respective accession numbers provided in the Data Availability Section?	Yes	Data availability section
Were **human clinical and genomic datasets** deposited in a public access-controlled repository in accordance to ethical obligations to the patients and to the applicable consent agreement?	Not Applicable	
Are **computational models** that are central and integral to a study available without restrictions in a machine-readable form? Were the relevant accession numbers or links provided?	Not Applicable	
If publicly available data were reused, provide the respective **data citations in the reference list**.	Not Applicable	
